# A new oxidatively stable ligand for the chiral functionalization of amino acids in Ni(II)–Schiff base complexes

**DOI:** 10.3762/bjoc.19.41

**Published:** 2023-04-27

**Authors:** Alena V Dmitrieva, Oleg A Levitskiy, Yuri K Grishin, Tatiana V Magdesieva

**Affiliations:** 1 Lomonosov Moscow State University, Dept. of Chemistry, Leninskie Gory 1/3, Moscow 119991, Russia

**Keywords:** asymmetric synthesis, chiral auxiliaries, cysteine derivatives, Ni–Schiff base complexes, voltammetry testing

## Abstract

A new oxidatively stable (*S*)-*N*-benzylproline-derived ligand ((*S*)-*N*-(2-benzoyl-5-*tert*-butylphenyl)-1-benzylpyrrolidine-2-carboxamide) and its Ni(II)–Schiff base complexes formed of glycine, serine, and dehydroalanine are reported. A bulky *tert*-butyl substituent in the phenylene fragment precludes unwanted oxidative dimerization of the Schiff base complex, making it suitable for targeted electrochemically induced oxidative modification of the amino acid side chain. Experimental and DFT studies showed that the additional *tert*-butyl group increases the dispersion interactions in the Ni coordination environment making the complexes more conformationally rigid and provides a higher level of thermodynamically controlled stereoselectivity as compared to the parent Belokon complex. Additionally, functionalization with the *tert*-butyl group significantly enhances the reactivity of the deprotonated glycine complex towards electrophiles as compared to the anionic species formed from the original Belokon complex. Solubility of the *t*-Bu-containing ligand and its Schiff base complexes is increased, facilitating scaling-up the reaction procedure and isolation of the functionalized amino acid.

## Introduction

**Scheme 1:**
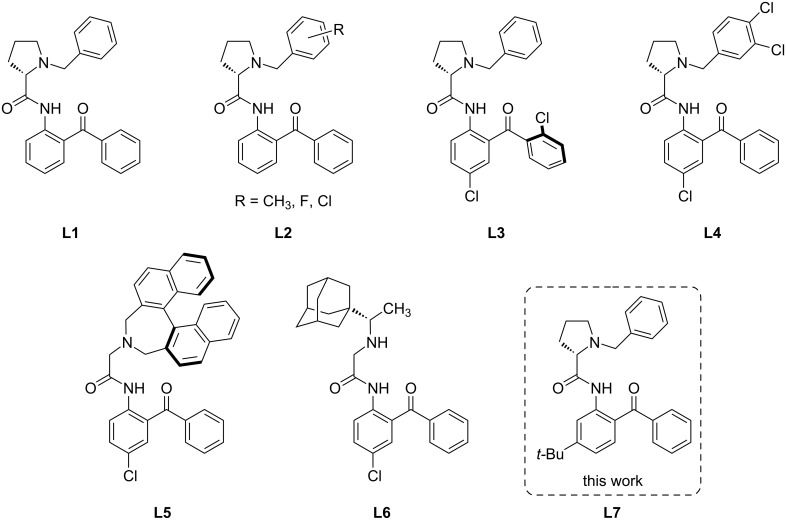
Selected examples of the chiral ligands used for synthesis of the Ni(II)–Schiff base complexes.

Asymmetric synthesis of functionalized amino acids is a subject of intense research because these compounds are of great demand for pharmaceutical industry, health care, and food production [1–3]. Various approaches to enantiomerically enriched amino acids have been developed employing chiral auxiliaries [4–5] and asymmetric phase-transfer catalysis [6–7]. The former approach is commonly based on the application of chiral derivatives of glycine containing structurally diverse chiral auxiliaries, both cyclic [8–11] and acyclic [12–13]. Transition-metal complexes derived from glycine Schiff bases containing a source of chirality is the most convenient and widely used template for modification of the glycine fragment under mild conditions. The template can be easily obtained via self-assembly of the starting components in the presence of metal ions (commonly Ni(II)) and includes a chiral auxiliary, an amino acid, and a bifunctional linker capable to arrange the components in the Schiff base complex. Such templates provide a significant C–H acidity at the α-amino acid carbon and a possibility for recycling of the chiral auxiliaries (for reviews see [5,14–18]). In early works, the chiral tridentate ligand based on (*S*)-*N*-benzylproline (**L1**) was used [19–20]. Though the original Belokon complex derived from *N*-benzylproline and *o*-aminobenzophenone showed sufficiently high efficiency and is still the most widely used template, considerable efforts on the modification of the chiral auxiliaries as well as of the other fragments of the tridentate ligand have been made to improve stereocontrolling efficiency and to modify physicochemical properties of the template (such as solubility, lipophilicity, etc). Thus, various substituents (4,5-di-CH_3_ [21], 2-Cl [21], 3,4-di-Cl [21–22], 2/3/4-F [23]) were inserted in the *N*-benzyl moiety as well as in the aromatic rings of the benzophenone fragment [24–26] (selected examples are given in Scheme 1).

Insertion of halogen atoms increased enantioselectivity, e.g., in alkylation reactions [27–28]. Functionalization of the phenyl ring in the benzophenone gives rise to an additional axial chirality (**L3**), thus improving stereoselectivity observed at the removed stereocenter [24–25]. Replacing *N*-benzylproline for 2,7-dihydro-1*H*-azepine (**L5**) allowed obtaining a new tridentate ligand with chemically stable axial chirality which exhibits no racemization under action of strong bases [29]. The chiral N–H-containing rimantadine-based ligand (**L6**) is highly lipophilic and it was successfully used for the kinetic resolution of unprotected racemic amino acids [30].

The examples mentioned above show that the metal–Schiff base chiral template is a fairly versatile “tool” that can be adapted to a specific task. A relatively new approach to functionalization of amino acids is a combination of a stereoselective synthesis in a metal-coordination environment with electrochemical activation [31]. It increases the reactivity and the reaction scope, making possible new types of transformations [32–35]. On the other hand, it puts additional requirements on the design of the chiral ligand. Another important structurally tunable parameter comprises the oxidation or reduction potential of the complex. Additionally, as it has been shown in our previous reports [31,36], the protection of the phenylene fragment with substituents is required, to allow oxidative transformations of the amino acid fragment. Otherwise, the oxidative coupling of two phenylene fragments via the *para*-positions may be the dominant reaction path yielding the bimetallic Ni(II) complex [36]. Thus, **L4** in Scheme 1 was shown to be suitable for the oxidative functionalization of the amino acid fragment [37].

A bulky *tert*-butyl group can be also proposed as a protecting moiety. One could expect that the *t*-Bu group will prevent fast oxidative dimerization of the Schiff base complex and the radical cation formed under one-electron electrochemical oxidation will be sufficiently stable, opening a route to further oxidative modification of the amino acid side chain under appropriate conditions. Additionally, this bulky group may significantly alter the whole steric construction of a molecule and give rise to additional interactions which would increase the stereocontrolling properties. This idea efficiently works in the enantioselective extraction of the unprotected amino acids [38–39]. In the case of the Ni–Schiff base complexes, the *t*-Bu group may give rise to additional dispersion interactions with the phenyl ring in the proline auxiliary, making the Schiff base complexes more conformationally rigid, thus increasing the stereochemical outcome of the functionalized amino acids as compared to the parent ligand **L1**.

In the present paper, the new structurally advantageous (*S*)-*N*-benzylproline-derived ligand containing the bulky *tert*-butyl substituent in the phenylene fragment (**L7**) is reported. Three Ni(II)–Schiff base derivatives of the new ligand (containing glycine, serine, and dehydroalanine) were obtained. Complexes of this type are most often used as the starting platform for the modification of an amino acid moiety by nucleophilic substitution and addition reactions, including electrochemically induced processes [5,16–18,31]. The electrochemical behavior of the new complexes was investigated to get an experimental support for their oxidative stability. The reactivity of the glycine and dehydroalanine complexes in the nucleophilic functionalization of amino acids was compared to the corresponding derivatives of the parent ligand **L1**. The results clearly demonstrated the advantages of the new complexes as stereocontrolling templates.

## Results and Discussion

### Synthesis

**Scheme 2:**
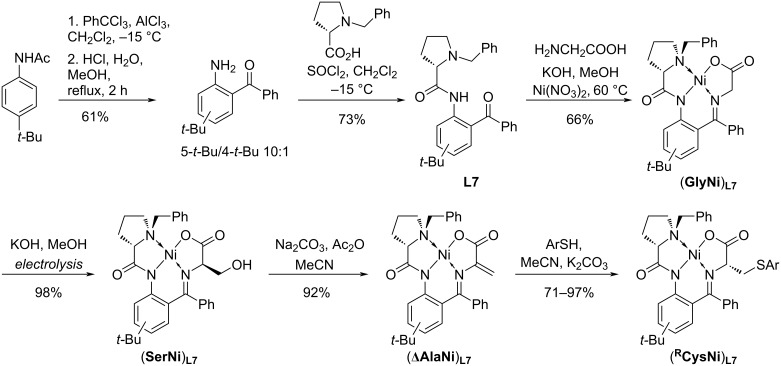
Synthesis of the chiral ligand **L7** and its Ni(II) complexes with glycine, serine, dehydroalanine, and cysteine derivatives.

The synthetic approach to the chiral ligand **L7** as well as to its Ni–Schiff base derivatives containing glycine, serine, dehydroalanine, and cysteine is given in Scheme 2. Commercially available (*S*)-proline was used as the starting material.

To obtain the starting *t*-Bu-substituted *o*-aminobenzophenone, the synthetic procedure reported in [40] was applied. For acylation of the *tert*-butylacetanilide, PhCCl_3_ in the presence of AlCl_3_ was used. In the reported [40] reaction conditions, a complicated mixture of products was obtained. Lowering the temperature to −15 °C (in contrast to rt in [40]) allowed obtaining the targeted compound; however, acylation was accompanied with the migration of the *t*-Bu group yielding 2-benzoyl-4-*tert*-butylaniline and the corresponding 5-butylated isomer in a 10:1 ratio. Our attempts to separate the isomers were unsuccessful. However, as it will be shown below, this is not necessary since the isomeric Schiff base template proved efficient in stereoselective modification of amino acids. The products of the oxidative dimerization via the phenylene ring were not detected for both isomeric templates.

The subsequent reaction with (*S*)-benzylproline lead to (*S*)-*N*-(2-benzoyl-5-*tert*-butylphenyl)-1-benzylpyrrolidine-2-carboxamide (**L7**) in 73% yield with 96% ee (see Supporting Information File 1). The self-assembly of the three components **L7**, Ni(NO_3_)_2_, and glycine gave the corresponding (**GlyNi**)**_L7_** complex which was isolated in 66% yield and fully characterized using spectral methods (for HRMS, ^1^H, ^13^C NMR, including 2D techniques, see Supporting Information File 1).

To obtain the serine derivative (**SerNi**)**_L7_**, the recently developed electrochemical approach for the stereoselective hydroxyalkylation [32] was used. The reaction protocol is operationally simple and suitable for gram-scale loadings. Galvanostatic electrolysis of the glycine complex (**GlyNi**)**_L7_** was performed in a one-compartment electrochemical cell in a methanol solution (CH_3_OH serves as a reactant and a solvent simultaneously) in the presence of KOH. By this route the (**SerNi**)**_L7_** complex (with ʟ-configuration at the α-stereocenter) was obtained in quantitative yield with high diastereselectivity (83% de). Previously, the same protocol was applied to the parent Belokon complex, affording the corresponding serine derivative in 97% yield with 81% de [32]. For spectral characterization of (**SerNi**)**_L7_**, see Supporting Information File 1.

The serine complex (**SerNi**)**_L7_** served as the precursor for (**ΔAlaNi**)**_L7_** which was obtained via dehydration using a commonly used procedure [41]. The new complex (**ΔAlaNi**)**_L7_** was isolated in 92% yield and fully characterized (HRMS, ^1^H, ^13^C NMR, including 2D techniques; for details, see Supporting Information File 1); [α]_D_^20^ = 2127 (MeOH).

To test the stereocontrolling efficiency of the new ligand **L7** and to obtain new cysteine Ni–Schiff base derivatives (**^R^CysNi**)**_L7_** which are of practical interest, complex (**ΔAlaNi**)**_L7_** was involved in a nucleophilic addition as Michael acceptor. A number of thiols was taken as the model compounds. The reaction was performed under thermodynamic conditions reported in [41], to compare the thermodynamically controlled stereoselectivity of **L7** and **L1**. The use of a 2 molar excess of K_2_CO_3_ induces the equilibrium between the isomers (established via the α-protonation/deprotonation in the intermediate α-carbanion in the amino acid moiety) and concomitant epimerization of the final product [41]. The results of the thiolation of complex (**ΔAlaNi**)**_L7_** are given in Table 1. The corresponding ʟ-cysteine derivatives (**^R^CysNi**)**_L7_** were isolated in high yields and with excellent stereoselectivity: only traces of the ᴅ-cysteine derivatives were determined, demonstrating the advantages of the *tert*-butyl-containing template (for the probable reasons, see below).

**Table 1:** The yields and the thermodynamically controlled stereoselectivity observed in the thiolation of (**ΔAlaNi**)**_L7_** complexes with RSH; and comparison with the data for (**ΔAlaNi**)**_L1_** reported previously [41] given in parentheses.^a^

R	Bn	Ph	*p*-CH_3_C_6_H_4_-	*o*-BrC_6_H_4_-	*p*-BrC_6_H_4_-

product	(**^Bn^CysNi**)**_L_**	(**^Ph^CysNi**)**_L_**	(**^pMe^CysNi**)**_L_**	(**^oBr^CysNi**)**_L_**	(**^pBr^CysNi**)**_L_**
yield	85% (97%)	73% (93%)	83%	71%	78%
dr (ʟ:ᴅ)^b^	>52:1 (19:1)	>44:1 (30:1)	>43:1	>29:1	32:1

^a^Conditions: 0.65 М **ΔAlaNi**, CH_3_CN/DMF, 2 equiv K_2_CO_3_, 1.05 equiv RSH, 50–55 °C. ^b^dr was determined as the ratio of the isolated diastereomers. For accuracy, less than 1 mg amounts of the minor isomer were rounded up to 1 mg.

**Figure 1:**
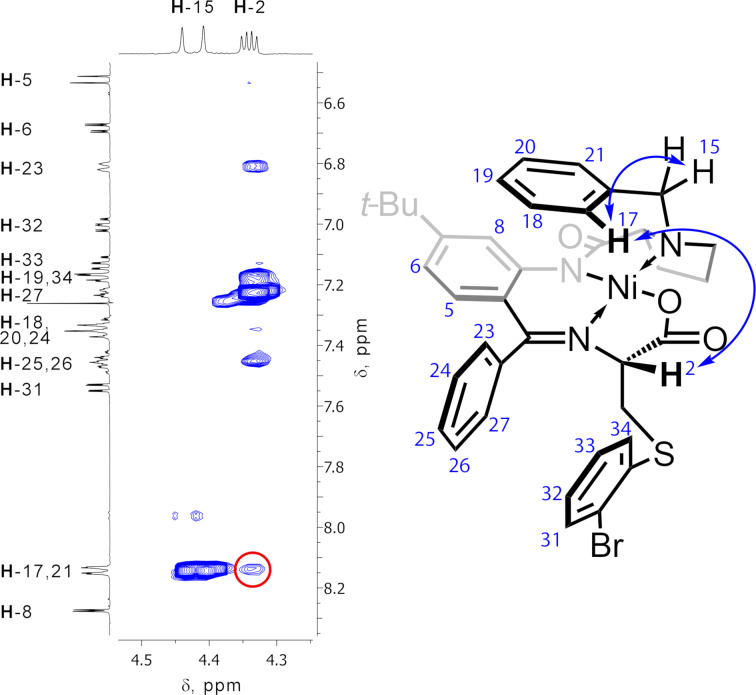
Fragment of the NOESY spectrum of the ʟ-(**^oBr^CysNi**)**_L7_** complex indicating the correlation between the H-2 and H-17,21 protons.

The relative ʟ-configuration of the amino acid α-stereocenter in the major diastereomers of (**^R^CysNi**)**_L7_** formed in the Michael addition reaction was confirmed using the NOESY spectrum. It is illustrated in Figure 1 for (**^oBr^CysNi**)**_L7_** complex as the representative example. A correlation between the signal of the H-2 proton at the α-amino acid stereocenter with the signal of the *ortho*-protons of the benzyl fragment was observed. This indicated that H-2 is located on the same side of the nickel coordination plane as the benzyl substituent at the proline nitrogen atom, leading to the ʟ-configuration of the α-amino acid stereocenter. Notably, the major stereoisomer of all thiolated compounds (**^R^CysNi**)**_L7_** has the same configuration (ʟ), which was additionally confirmed by the similarity of the H-8 proton chemical shifts for all these complexes (the value falls within the 8.27–8.30 ppm range). It should be emphasized that the H-8 chemical shift is very sensitive to the configuration of the α-stereocenter [32]; a significant downfield shift of this signal (up to 0.5 ppm) could be expected for the diastereomer with the ᴅ-α-stereocenter with respect to the ʟ-isomer.

To prove the recyclability of the *t*-Bu-containing (*S*)-*N*-benzylproline-derived ligand **L7**, the ʟ**-**(**^pMe^CysNi**)**_L7_** complex was decomposed by heating of its MeOH/HCl solution at 60 °C under conditions similar to the ones previously reported for (**^R^CysNi**)**_L4_** [42] (see Supporting Information File 1 for details). The corresponding amino acid was obtained, along with the **L7** ligand which was recovered in ca. 70% yield and then reused.

### DFT study

The level of stereoselectivity induction is important for the evaluation of the chiral templates efficiency. The estimation of the thermodynamically and kinetically controlled stereoselectivity is based on different approaches. The diastereomeric ratio of the products formed under kinetic control is related to the transition-state energy. However, the problem is that the transition state is rather specific and dependent on the particular reaction type. Thus, it hardly can be generalized. In contrast, the thermodynamically controlled stereoselectivity refers to the difference in the thermodynamic stability of the (*S*)- and (*R*)-diastereomers which is related to the conformational flexibility of the template. The rigidity of the chiral Ni–Schiff base complexes is mainly determined by the noncovalent interactions in the Ni coordination environment. Among them, the most important is the π-stacking between the *o*-phenylene fragment and the benzyl moiety in the proline. The stronger the π-stacking interactions are, the more rigid are the complexes and the higher is the difference in the relative energies of the amino acid derivatives with different configuration of the α-stereocenter. As it has been shown previously [31,37], this difference determines the stereochemical outcome of the reactions performed under thermodynamic control.

To determine the conformational changes in the Ni(II) coordination environment induced by the additional *tert*-butyl group in the *o*-phenylene moiety, DFT calculations were performed for the model diastereomeric α-ʟ- and α-ᴅ-alanine complexes derived from new ligand **L7**; the approach for visualization of the noncovalent interactions suggested in [43] was applied.

**Figure 2:**
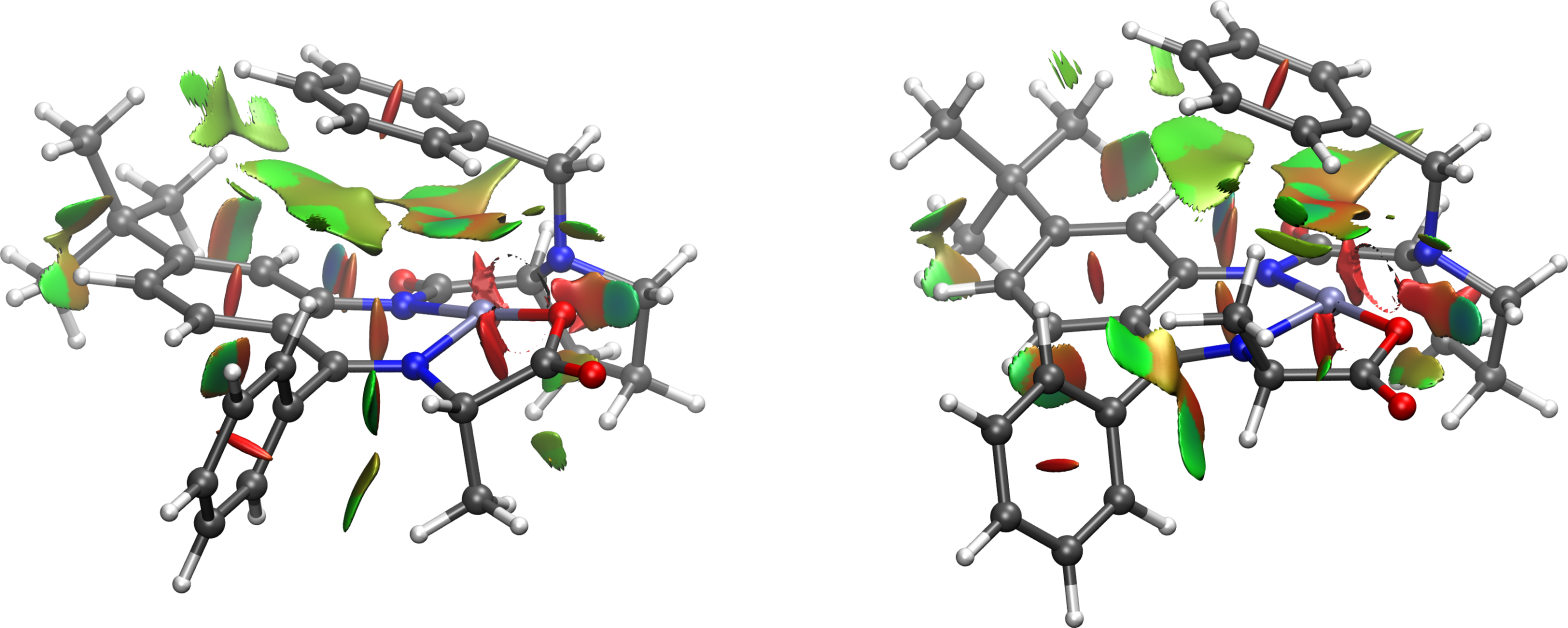
Low-gradient isosurfaces with low densities (blue color of the isosurface corresponds to the hydrogen bonding; the dispersion interactions (van der Waals interactions, the π-stacking) are marked in green color; red color indicates steric clashes) obtained for the ʟ- (left image) and ᴅ-alanine (right image) Ni–Schiff base derivatives with ligand **L7**.

The images obtained for the ʟ**-** and ᴅ**-**(**AlaNi**)**_L7_** complexes are given in Figure 2. The types of interactions are presented in different colors: the hydrogen bonding are labeled in blue color of the reduced density gradient isosurface; green color corresponds to the dispersion interactions (van der Waals interactions, the π-stacking); red color represents steric clashes. The interplay of these through-space interactions gives rise to the difference in the relative energies of the ʟ*-* and ᴅ-isomers. The most important are the π-stacking between the benzyl group in the proline fragment and the *o*-phenylene moiety which bring these two fragments closer to each other. These interactions are much stronger in the α-ʟ isomer (see Figure 2). The presence of the *tert*-butyl group induces an additional dispersion interaction between this group and the benzyl fragment in the ʟ**-**(**AlaNi**)**_L7_** complex, bringing the benzyl group and the *o*-phenylene moiety even closer to each other (the distance between the C20 and C8 atoms is 3.45 Å in ʟ**-**(**AlaNi**)**_L7_** and 3.49 Å in ʟ**-**(**AlaNi**)**_L1_**; for numbering of the atoms, see Figure 1). This makes the former complex more conformationally rigid. Indeed, the calculated (PBE-D4 /def2-SVP) energy difference between the ᴅ- and ʟ-diastereomers is more significant in (**AlaNi**)**_L7_** than in (**AlaNi**)**_L1_** (3.6 kcal/mol vs 3.3 kcal/mol, see Table 2) giving rise to a higher level of thermodynamically controlled stereoselectivity in the ligand **L7**-derived complexes.

**Table 2:** The calculated (PBE-D4 /def2-SVP) energy differences between the ᴅ*-* and ʟ-(**AlaNi**).

Complex	(**AlaNi**)**_L7_**	(**AlaNi**)**_L1_**	(**AlaNi**)**_L4_** [37]

Δ*E*, kcal/mol	3.6	3.3	3.7

However, the energy difference between the ᴅ- and ʟ-diastereomeric (**AlaNi**)**_L7_** is somewhat lower than that for (**AlaNi**)**_L4_** (3.6 kcal/mol vs 3.7 kcal/mol, see Table 2); consequently, a stereoinduction efficiency expected for ligand **L7** may be lower than that of **L4**. However, taking into account the simplicity of our procedure and the atom-economy principle, the ligand **L7** can be considered as a cheaper alternative to **L4**, still providing a significant stereoselectivity level.

**Figure 3:**
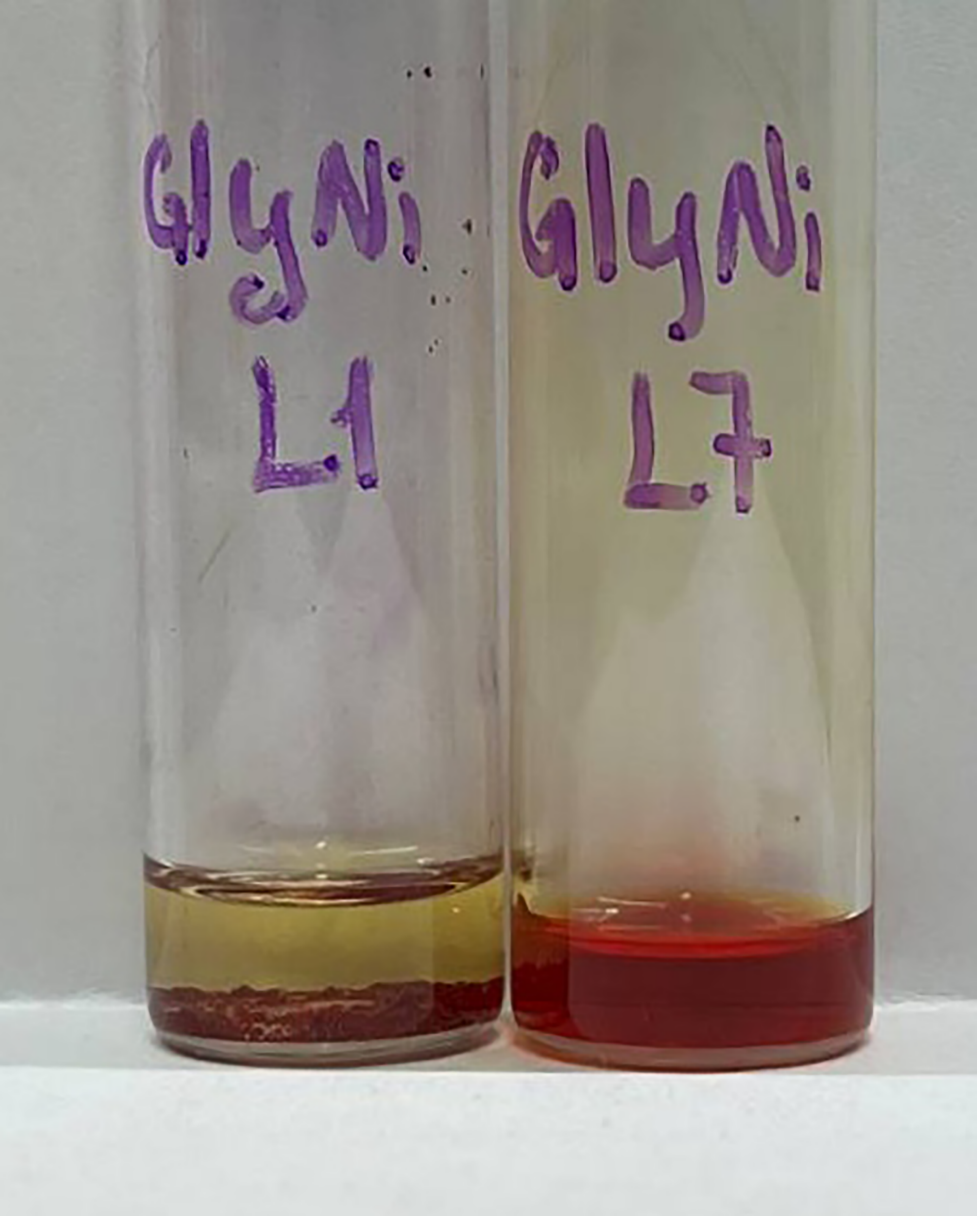
Saturated solutions of (**GlyNi**)**_L1_** (left) and (**GlyNi**)**_L7_** (right) in diethyl ether.

Additionally, Schiff base derivatives of **L7** have a considerably higher solubility in acetonitrile as compared to the **L4-**based complexes (see Table 3 and Supporting Information File 1); this makes it easier to scale-up the synthesis (e.g., for the S_N_2 alkylations or nucleophilic addition including the thiolation discussed above). Insertion of the *t*-Bu group also makes the (**GlyNi**)**_L7_** complex more soluble in diethyl ether as compared to the parent (**GlyNi**)**_L1_** complex (21.3 mg/1 mL vs <1 mg/1 mL in Et_2_O; see Figure 3). This is beneficial for electrosynthesis, simplifying the separation of the complexes from the supporting electrolyte (which is usually insoluble in Et_2_O).

**Table 3:** The solubility (mg/1 mL of CH_3_CN) of the complexes derived from ligands **L7** and **L4**.

Complex	(**ΔAlaNi**)**_L_**	(**^Bn^CysNi**)**_L_**	(**^pMe^CysNi**)**_L_**

**L4**	9	8	5
**L7**	>98	>536	560

### Electrophilicity of the deprotonated (**GlyNi**)**_L7_** complex

The α-deprotonated Ni–Schiff base derivatives of glycine (which can be considered as a chiral nucleophilic equivalent of glycine) can be involved in various types of chemical transformations. The oxidation potential value of the deprotonated glycine complex correlates with the reactivity of the α-carbanionic species towards electrophiles [37]. Therefore, the electrochemical approach is very convenient and informative for the estimation of the relative reactivity of the complexes derived from various chiral ligands. The oxidation potential can be determined from the voltammetry curve measured for the quantitatively deprotonated complex. The electrochemical deprotonation using an electro-generated base is the most convenient approach [37,44]. Comparison with the *E*^ox^ values determined previously for the parent Belokon complex (**GlyNi**)**_L1_** (−0.32 V vs Ag/AgCl/KCl_(sat.)_ [37]) and (**GlyNi**)**_L4_** (−0.22 V [37]) showed that the nucleophilic equivalent of (**GlyNi**)**_L7_** (*E*^ox^ = −0.41 V, see Table 4) should be much more reactive towards electrophiles than the deprotonated (**GlyNi**)**_L1_** and (**GlyNi**)**_L4_** complexes.

**Table 4:** Oxidation and reduction potential values for (**GlyNi**)**_L7_** and (**ΔAlaNi**)**_L7_** and comparison with previously reported data for (**GlyNi**)**_L1_**, (**ΔAlaNi**)**_L1_**, (**GlyNi**)**_L4_** and (**ΔAlaNi**)**_L4_** [36–37] (Pt, CH_3_CN, 0.1 M Bu_4_NBF_4_, vs Ag/AgCl/KCl_(sat.)_, 100 mV/s).

Complex	Ligand **L7**		Ligand **L1** [36–37]		Ligand **L4** [36–37]	
	Oxidation, V	Reduction, V	Oxidation, V	Reduction, V	Oxidation, V	Reduction, V

**ΔAlaNi**	*Е*_p_ = 1.32 *Е*_1/2_ = 1.285	*Е*_p_ = −1.34 *Е*_1/2_ = −1.31	*Е*_p_ = 1.34 *Е*_1/2_ = 1.255	*Е*_p_ = −1.30	*Е*_p_ = 1.4 *Е*_1/2_ = 1.37	*Е*_p_ = −1.32
**GlyNi**	*Е*_p_ = 1.31 *Е*_1/2_ = 1.27	*Е*_p_ = −1.59 *Е*_1/2_ = −1.53	*Е*_p_ = 1.32 *Е*_1/2_ = 1.24	*Е*_p_ = −1.57 *Е*_1/2_ = −1.51	*Е*_p_ = 1.4 *Е*_1/2_ = 1.36	*Е*_p_ = −1.53 *Е*_1/2_ = −1.43
**Gly_−H_Ni** (deprotonated)	*Е*_p_ = −0.41	–	*Е*_p_ = −0.32	–	*Е*_p_ = −0.22	–

### Electrochemical properties

For further applications of the (**GlyNi**)**_L7_** and (**ΔAlaNi**)**_L7_** complexes in electrochemically activated functionalization of amino acid fragments, a more detailed electrochemical study is an important precondition. Electrochemical properties were investigated using cyclic voltammetry (CV) in acetonitrile with 0.1 M Bu_4_NBF_4_ as a supporting electrolyte. A platinum disk was used as a working electrode. Formal potential values are given in Table 4. As could be expected, the potential values are rather close to that for the corresponding derivatives of the original ligand **L1** [37].

**Figure 4:**
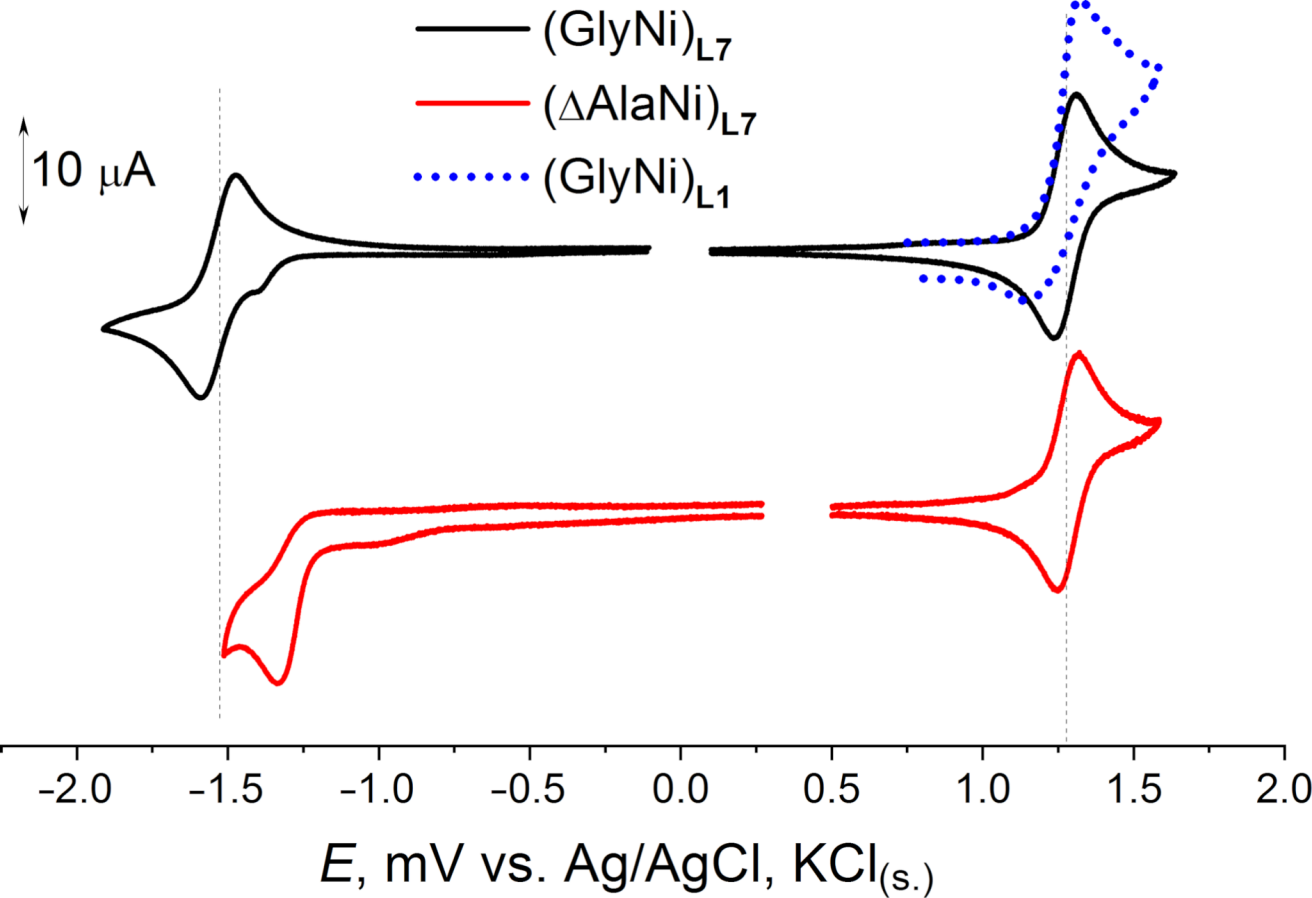
The CV curves observed for (**GlyNi**)**_L7_** and (**ΔAlaNi**)**_L7_** in the anodic and cathodic regions (Pt, CH_3_CN, 0.1 M Bu_4_NBF_4_, 100 mV/s, vs Ag/AgCl/KCl_(sat.)_). Oxidation of (**GlyNi**)**_L1_** is given for comparison.

Meanwhile, an important difference is the reversibility of the oxidation observed at low potential scan rates (100 mV/s) for the complexes (**GlyNi**)**_L7_** and (**ΔAlaNi**)**_L7_** (see Figure 4), in contrast to their *t*-Bu-free analogs. The similarity of the potential values for the complexes derived from **L1** and **L7** allows assuming that the HOMO/LUMO localization is not significantly influenced by the *tert*-butyl group. Indeed, the HOMO is a combination of the π-orbitals of the substituted phenylene fragment, the p-orbital of the amide nitrogen and the Ni d-orbitals (see Supporting Information File 1). Although the phenylene fragment in the oxidized (**GlyNi**)**_L7_** and (**ΔAlaNi**)**_L7_** complexes bears a certain spin density (as it has been previously shown for the *t*-Bu-free analogs [31]), the bulky *t*-Bu substituent does prevent dimerization of the radical cations formed in the electron-transfer step increasing their kinetic stability. This result is important; it indicates the possibility for further oxidative functionalization of the amino acid fragment using the Ni–Schiff base templates derived from the new ligand **L7**.

One-electron reduction of the complex (**GlyNi**)**_L7_** is mainly metal centered (with some impact of the π* orbital of the C=N bond), diffusion controlled (the slope of the ln *i*_pc_ vs ln *v* dependence is 0.48), and reversible; the direct and reverse peak separation value was 57 mV at 100 mV/s, and Ia/Ic peak current ratio was close to unity. This means that the radical anion of (**GlyNi**)**_L7_** is stable, at least in the cyclic voltammetry time scale. The spin density in the radical anion is mainly localized on the Ni d-orbitals and on the N and O atoms of its coordination environment (see Supporting Information File 1).

The reduction of the complex (**ΔAlaNi**)**_L7_** is irreversible at scan rates lower 2000 mV/s. Full reversibility can be reached at scan rates above 20 V/s allowing the *E*_1/2_ estimation. Similarly to previously studied complex (**ΔAlaNi**)**_L1_** [36], electron transfer to (**ΔAlaNi**)**_L7_** is mainly ligand centered. The DFT-estimated LUMO is formed as an overlap of the π antibonding orbitals of the dehydroalanine and imine fragments with much smaller impacts of the Ni orbitals and π orbitals of the aromatic moiety. The radical anion formed behaves like a nucleophilic С-radical, reacting with the C–C double bond of the starting **ΔAlaNi** complex yielding the dimeric binuclear complex.

## Conclusion

The new structurally advantageous (*S*)-*N*-benzylproline-derived ligand (*S*)-*N*-(2-benzoyl-5-*tert*-butylphenyl)-1-benzylpyrrolidine-2-carboxamide and its Ni(II)–Schiff base complexes formed of glycine, serine, and dehydroalanine are reported. The bulky *tert*-butyl substituent inserted in the phenylene fragment of the ligand allowed to solve the following problems. First of all, radical cations formed under a one-electron electrochemical oxidation of the glycine and dehydroalanine complexes become sufficiently stable. The fast side reaction of the oxidative dimerization of the Schiff base complex via the phenylene fragments (inherent to the original Belokon complexes) is prevented, thus opening a route to the targeted electrochemically induced oxidative modification of the amino acids side chain. Second, as follows from the comparison of the oxidation potential values, the reactivity of the deprotonated glycine complex towards electrophiles is significantly enhanced as compared to the anionic species formed from the original Belokon complex. Third, the dehydroalanine complex showed the superior performance in the stereoselective arylthiolation of the double bond yielding the cysteine derivatives as compared to the original complex derived from *N*-benzylproline. The quantum chemical study showed that the *t*-Bu group increases the dispersion interactions between the benzyl group in the proline fragment and the *o*-phenylene moiety making the Ni(II) complex more conformationally rigid and providing a higher level of thermodynamically controlled stereoselectivity. Finally, the solubility of the *t*-Bu-containing ligand and its Schiff base complexes is increased, facilitating scaling-up the reaction procedure and isolation of the functionalized amino acid.

We hope that the chiral templates derived from the easily available and inexpensive new ligand and amino acids (glycine and dehydroalanine) will find their rightful place in the stereoselective synthesis of tailor-made amino acids (including oxidative transformations).

## Supporting Information

File 1 Experimental details, characterization, and copies of spectra.
