# Supplementary material for: A new oxidatively stable ligand for the chiral functionalization of amino acids in Ni(II)–Schiff base complexes
Source: Beilstein J Org Chem. 2023 Apr 27;19:566–74. doi: 10.3762/bjoc.19.41 (PMC10155621; doi:10.3762/bjoc.19.41)
Supplement: File 1 — Experimental details, characterization, and copies of spectra. [file Beilstein_J_Org_Chem-19-566-s001.pdf]

BEILSTEIN JOURNAL OF ORGANIC CHEMISTRY

Supporting Information

for

A new oxidatively stable ligand for the chiral functionalization of amino acids in Ni(II)–Schiff base complexes

Alena V. Dmitrieva, Oleg A. Levitskiy, Yuri K. Grishin and Tatiana V. Magdesieva

Beilstein J. Org. Chem. **2023,** *19,* 566–574. doi:10.3762/bjoc.19.41

Experimental details, characterization, and copies of spectra

License and Terms: This is a supporting information file under the terms of the Creative Commons Attribution License (https://creativecommons.org/licenses/by/4.0). Please note that the reuse, redistribution and reproduction in particular requires that the author(s) and source are credited and that individual graphics may be subject to special legal provisions.
The license is subject to the *Beilstein Journal of Organic Chemistry* terms and conditions: (https://www.beilstein-journals.org/bjoc/terms)

Table of contents

1. General information S2
2. Synthesis and characterization data for all compounds S3
   2.1. 2-Benzoyl-5-*tert*-butylaniline S3
   2.2. (*S*)-2-[*N*-(*N'*-Benzylprolyl)amino]-4-*tert*-butylbenzophenone **(L7)** S4
   2.3. Complex **(GlyNi)$_{L7}$** S4
   2.4. Complex **(SerNi)$_{L7}$** S5
   2.5. Complex **(ΔAlaNi)$_{L7}$** S6
   2.6. General procedure for synthesis of complexes **(^{R}CysNi)$_{L7}$** S7
   2.7. Complex **(^{Bn}CysNi)$_{L7}$** S7
   2.8. Complex **(^{Ph}CysNi)$_{L7}$** S7
   2.9. Complex **(^{pMe}CysNi)$_{L7}$** S8
   2.10. Complex **(^{oBr}CysNi)$_{L7}$** S8
   2.11. Complex **(^{pBr}CysNi)$_{L7}$** S9
   2.12. L-*S*-tolylcysteine S9
3. NMR spectra for all compounds S10
   3.1. ^{1}H NMR spectrum of 2-benzoyl-5-*tert*-butylaniline S10
   3.2. ^{13}C NMR spectrum of 2-benzoyl-5-*tert*-butylaniline S11
   3.3. HSQC spectrum of 2-benzoyl-5-*tert*-butylaniline S12
   3.4. HMBC spectrum of 2-benzoyl-5-*tert*-butylaniline S12
   3.5. ^{1}H NMR spectrum of compound **L7** S13
   3.6. ^{13}C NMR spectrum of compound **L7** S14
   3.7. HSQC spectrum of compound **L7** S15
   3.8. HMBC spectrum of compound **L7** S16
   3.9. ^{1}H NMR spectrum of complex **(GlyNi)$_{L7}$** S17
   3.10. ^{13}C NMR spectrum of complex **(GlyNi)$_{L7}$** S18
   3.11. ^{1}H NMR spectrum of complex **(2*R*)-(SerNi)** S19
   3.12. ^{13}C NMR spectrum of complex **(2*R*)-(SerNi)** S21
   3.13. HSQC spectrum of complex **(2*R*)-(SerNi)** S21
   3.14. ^{1}H NMR spectrum of complex **(2*S*)-(SerNi)** S22
   3.15. ^{1}H NMR spectrum of complex **(ΔAlaNi)$_{L7}$** S23
   3.16. ^{13}C NMR spectrum of complex **(ΔAlaNi)$_{L7}$** S25
   3.17. HSQC spectrum of complex **(ΔAlaNi)$_{L7}$** S26
   3.18. HMBC spectrum of complex **(ΔAlaNi)$_{L7}$** S27
   3.19. ^{1}H NMR spectrum of complex **(^{Bn}CysNi)$_{L7}$** S28
   3.20. ^{13}C NMR spectrum of complex **(^{Bn}CysNi)$_{L7}$** S29
   3.21. HSQC spectrum of complex **(^{Bn}CysNi)$_{L7}$** S30
   3.22. HMBC spectrum of complex **(^{Bn}CysNi)$_{L7}$** S31
   3.23. ^{1}H NMR spectrum of complex **(^{Ph}CysNi)$_{L7}$** S33
   3.24. ^{13}C NMR spectrum of complex **(^{Ph}CysNi)$_{L7}$** S34
   3.25. ^{1}H NMR spectrum of complex **(^{pMe}CysNi)$_{L7}$** S35
   3.26. ^{13}C NMR spectrum of complex **(^{pMe}CysNi)$_{L7}$** S37
   3.27. ^{1}H NMR spectrum of complex **(^{oBr}CysNi)$_{L7}$** S38
   3.28. ^{13}C NMR spectrum of complex **(^{oBr}CysNi)$_{L7}$** S39
   3.29. HSQC spectrum of complex **(^{oBr}CysNi)$_{L7}$** S40
   3.30. HMBC spectrum of complex **(^{oBr}CysNi)$_{L7}$** S42
   3.31. COSY spectrum of complex **(^{oBr}CysNi)$_{L7}$** S43

3.32. NOESY spectrum of complex **(^{oBr}CysNi)$_{L7}$** S45
3.33. ^{1}H NMR spectrum of complex **(^{pBr}CysNi)$_{L7}$** S47
3.34. ^{13}C NMR spectrum of complex **(^{pBr}CysNi)$_{L7}$** S48
3.35. ^{1}H NMR spectrum of L-S-tolylcysteine S49
3.36. ^{13}C NMR spectrum of L-S-tolylcysteine S50
4. Mass spectra for all compounds S50
4.1. GC–MS data for 2-benzoyl-5-*tert*-butylaniline S50
4.2. ESI-HRMS data for compound **L7** S52
4.3. ESI-HRMS data for complex **(GlyNi)$_{L7}$** S52
4.4. ESI-HRMS data for complex **(2R)-(SerNi)$_{L7}$** S53
4.5. ESI-HRMS data for complex **(2S)-(SerNi)$_{L7}$** S53
4.6. ESI-HRMS data for complex **(ΔAlaNi)$_{L7}$** S54
4.7. ESI-HRMS data for complex **(^{Bn}CysNi)$_{L7}$** S54
4.8. ESI-HRMS data for complex **(^{Ph}CysNi)$_{L7}$** S55
4.9. ESI-HRMS data for complex **(^{pMe}CysNi)$_{L7}$** S55
4.10. ESI-HRMS data for complex **(^{oBr}CysNi)$_{L7}$** S56
4.11. ESI-HRMS data for complex **(^{pBr}CysNi)$_{L7}$** S57
5. IR spectra S58
5.1. IR spectrum of 2-benzoyl-5-*tert*-butylaniline S58
5.2. IR spectrum of compound **L7** S59
6. HPLC traces S59
7. Computational data (energies and Cartesian coordinates of the optimized structures) S62
8. Frontier orbitals of the (GlyNi)$_{L7}$ and its radical anion S71
9. References S72

1. General information

All reactants and solvents were commercially available from Aldrich and purified prior to the experiments. Silicagel 60M 0.04–0.063 mm was used for column chromatography.

All reactants and solvents were commercially available from Aldrich and purified prior to the experiments. Silicagel 60M 0.04–0.063 mm was used for column chromatography. Voltammetric experiments were performed with a Biologic BP-300 potentiostat, in an ALS Co. three-electrode cell of 2 mL with a platinum wire counter electrode (CE) and anhydrous Ag/0.01 M $AgNO_3$ (MeCN) reference electrode (RE). Ferrocene was used as internal standard in each experiment and all measured potentials were converted to the Ag/AgCl,KCl$_{(sat.)}$ reference electrode (in the latter scale, the potential for the $Fc^{+/0}$ redox couple is equal to 0.475 V in acetonitrile). A Pt disk electrode with an active surface area of 0.077 cm^2 was used as the working electrode (WE). The Pt electrode was polished with Al_2O_3 suspension SP-A 0.3 mm on a polishing pad (Metrohm, Germany), washed with sulfuric acid and rinsed with water and acetone. Hardware ohmic drop compensation was employed. All solutions were thoroughly deaerated by passing an argon flow through the solution prior to the CV experiments and above the solution during the measurements, the supporting electrolyte in all experiments was 0.1 M *n*-Bu_4NBF_4 (Aldrich, purity > 99%), which was recrystallized from water and dried by gentle heating under reduced pressure (0.05 Torr) prior to use. Acetonitrile (AN, Aldrich spectroscopic quality, < 0.02% water content) was distilled over P_2O_5 and stored under argon.

Electrosynthesis of SerNi was performed in an undivided cell of 250 mL volume with an AutoLab PGSTAT100N potentiostat. A glassy carbon plate (15 cm^2) was used as a working electrode and a platinum plate (10 cm^2) was used as a counter electrode.

Mass spectra were recorded with an AB Sciex TripleTOF 5600+ instrument using electrospray ionization (DuoSpray ESI). GC-MS analysis was performed on a system consisting of a gas chromatograph Agilent 8890 and a mass spectrometer 5977B Inert Plus MSD Turbo EI Bundle with an electron ionization source. Electron energy was 70 eV. ^1H (400.1 MHz) and ^{13}C (100.6 MHz) NMR spectra (including COSY, HMBC, HSQC, NOESY) were recorded using an Aglient 400-MR spectrometer in $CDCl_3$. Chemical shifts were referenced to the nondeuterated aliquot of the solvent. Specific rotations were measured with a Krüss P8000 polarimeter. Chiral HPLC analyses were performed using HPLC (Shimadzu) equipped with Daicel Chiralpak IA-3 column (4.6 × 150 mm), Daicel Chiralpak IB-3 column (4.6 × 150 mm), and a diode array detector. Computational details: Stationary-point structure searches were performed using the ORCA quantum chemistry package [1]. The gradient-corrected exchange-correlation Perdew, Burke, and Ernzerhof (PBE) functional [2], RI-J approximation, atom-pairwise dispersion correction to the DFT energy (D4) [3] and def2-SVP valence double-zeta basis set with polarization functions of the Karlsruhe group [4] along with def2/J [5] auxiliary basis set were applied. A threshold of 1×10^{-8} Hartree was used for SCF convergence; thresholds of 1×10^{-6} Hartree and 3×10^{-5} Hartree $Bohr^{-1}$ on energy and RMS gradient, respectively, were employed in optimization procedures. Kohn–Sham orbitals were used as input for noncovalent interaction (NCI) analysis. Reduced density gradient (for NCI visualization) was calculated in Multiwfn program [6]. IR spectra were recorded using a Nicolet iS5 (Thermo Scientific) Fourier transform spectrophotometer using an internal reflectance attachment with diamond optical element; attenuated total reflection (ATR, iD7) with 45° angle of incidence. Resolution was 4 cm^{-1}, the number of scans was 20.

The solubility of the complexes was measured by the preparation of saturated solutions (complexes were not dissolved completely), subsequent evaporation of the solution above the precipitate, and weighing the mass of the dissolved complex.

2. Synthesis and characterization data for all compounds

2.1. 2-Benzoyl-5-tert-butylaniline

In a manner similar to [7], to a cooled solution (0 °C) of anhydrous $AlCl_3$ (2.4 g, 18 mmol) in 5.2 mL of 1,2-dichloroethane, $PhCCl_3$ (0.8 mL, 5.6 mmol) was added dropwise in 15–20 min under efficient stirring. Afterwards, acetanilide (1 g, 5.2 mmol) was added at −40 °C. The mixture was warmed to −15 °C and stirred overnight at this temperature and then poured onto crushed ice and stirred at 40 °C for 0.5 h. Afterwards, the reaction mixture was diluted with water, extracted with CH_2Cl_2, washed with water and dried over Na_2SO_4. The obtained compound was used in the next step without further purification. To remove the acetyl group, the obtained compound was dissolved in a mixture of methanol and 6 M HCl (1:1) and refluxed for 2 h. Afterwards, the reaction mixture was cooled to room temperature, diluted with water, extracted with ethyl acetate, washed with water and dried over Na_2SO_4. The organic layer was evaporated under reduced pressure; the residue was purified by column chromatography (silicagel, ethyl acetate/hexane 1:5, v/v) to give the mixture of isomers 5-*t*-Bu/4-*t*-Bu in the ratio ≈ 10:1 as a brown oil (0.81 g, 61%). The ratio of isomers was estimated by ^1H NMR.

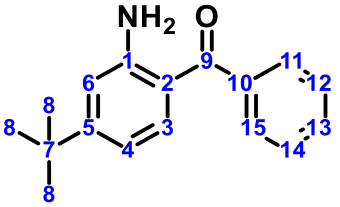

^{1}H NMR ($CDCl_3$ δ, ppm): 7.66 – 7.61 (m, 2H (H-11,15)), 7.54 – 7.49 (m, 1H (H-13)), 7.48 – 7,44 (m, 2H (H-12,14)), 7.40 (d, 1H, ^3J = 8.5 Hz, (H-3)), 6.73 (d, ^4J = 1.8 Hz, 1H (H-6)), 6,65 (dd, ^3J = 8.5 Hz, ^4J = 1.8 Hz, 1H (H-4)), 6.16 (s, 2H (NH)), 1.29 (s, 9H (H-8)).

^{13}C NMR ($CDCl_3$ δ, ppm): 198.63 (C-9), 158.27 (C-5), 151.22 (C-1), 140.51 (C-10), 134.60 (C-3), 130.86 (C-13), 129.06 (C-11, C-15), 128.12 (C-12, C-14), 115.92 (C-2), 113.69 (C-6 or C-4), 113.66 (C-4 or C-6), 35.07 (C-7), 30.93 (C-8).

GC-MS: m/z 253

IR υ, cm^{-1}: 1585 (δ NH), 1620 (ν CO), 2963 (ν CH_3 of tBu), 3345 (ν_s NH_2), 3469 (ν_{as} NH_2).

2.2. *(S)-2-[N-(N'-Benzylprolyl)amino]-4-tert-butylbenzophenone **(L7)***

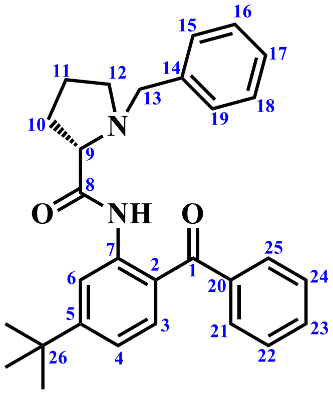

In a manner similar to [8], a solution of (*S*)-benzylproline (1.05 g, 5.1 mmol) in 5.1 mL of CH_2Cl_2 was cooled to −40 °C, and $SOCl_2$ (0.27 mL, 3.7 mmol) was added dropwise. Then, a solution of 2-benzoyl-4-*tert*-butylaniline (0.8 g, 3.1 mmol) in 2.5 mL of CH_2Cl_2 was added at −40°C. The cooling bath was removed, and the reaction mixture was stirred at room temperature overnight. Afterwards, the mixture was cooled with an ice bath, and a solution of Na_2CO_3 (1.23 g, 11.6 mmol) in water (5 mL) was added. The organic layer was separated, the aqueous layer was extracted with CH_2Cl_2, the organic solutions were combined, dried over Na_2SO_4 and evaporated under reduced pressure. The residue was purified by column chromatography (silicagel, ethyl acetate/hexane 1:2) to give a brown oil (1.02 g, 73%). The product is a mixture of isomers (5-*t*-Bu/4-*t*-Bu) in the ratio of ≈ 10:1. er 98:2. HPLC (Daicel IA-3, heptane/isopropanol 95:5, flow rate: 1 mL/min, l = 244 nm) t_{R1} = 3.92 (minor), t_{R2} = 4.26 (major). For HPLC analyses racemic 2-[*N*-(*N*'-benzylprolyl)amino]-4-*tert*-butylbenzophenone was synthesized starting with racemic proline according to the same procedures that was used for (S)-**7**.

HRMS (ESI): m/z 441.2548 (M+H$^+$, 441.2537 calculated for $C_{29}H_{33}N_2O_2$).

^{1}H NMR ($CDCl_3$ δ, ppm): 11.73 (s, 1H (NH)), 8.72 (d, ^4J = 1.9 Hz, 1H, (H-6)), 7.79 – 7.76 (m, 2H (H-21,25), 7.62 – 7.57 (m, 1H (H-23)), 7.52 –7.46(m, 2H (22,24)), 7.45 (d, ^3J = 8.5, 1H (H-3)), 7.44 – 7.40 (m, 2H (H-15,19)), 7.18 – 7.14 (m, 3H (H-16,17,18)), 7.11 (dd, ^3J = 8.5, ^4J = 1.9 Hz, 1H (H-4)), 3.98 (d, ^2J = 13.0 Hz, 1H (H-13)), 3.60 (d, ^2J = 13.0 Hz, 1H (H-13)), 3.34 (dd, ^3J = 10.1, 4.9 Hz, 1H (H-9)), 3.27 – 3.21 (m, 1H (H-12)), 2.45 – 2.37 (m, 1H (H-12)), 2.33 – 2.21 (m, 1H (H-10)), 2.04 – 1.96 (m, 1H (H-10)), 1.92 – 1.73 (m, 2H (H-11)), 1.37 (s, 9H).

^{13}C NMR ($CDCl_3$ δ, ppm): 198.00 (C-1), 174.85 (C-8), 157.69 (C-5), 139.61 (C-20), 139.05 (C-7), 138.31 (C-14), 132.90 (C-3), 132.30 (C-23), 130.05 (C-21,25), 129.21 (C-15,19), 128.31 (C-22,24), 128.23 (C-16,18), 127.09 (C-17), 122.55 (C-2), 119.43 (C-4), 118.76 (C-6), 68.56 (C-9), 59.88 (C-13), 53.86 (C-12), 35.52 (C-26), 31.12 (CH_3 of tBu), 31.03 (C-10), 24.22 (C-11).

2.3. *Complex **(GlyNi)$_{L7}$***

In a manner similar to [8], a two-necked round-bottomed flask was charged with $Ni(NO_3)_2{\cdot}6H_2O$ (1.34 g, 4.6 mmol) and glycine (0.863 g, 11.5 mmol). A solution of **7** (1.02 g, 2.3 mmol) in methanol (8 mL) was added under argon and then the obtained mixture was deaerated and heated to 45–50 °C. A solution of KOH (0.902 g, 16 mmol) in methanol (3.5 mL) was added, and

the mixture was stirred for 1.5 h at 60 °C, then neutralized with AcOH (0.92 mL, 16.1 mmol) and cooled to room temperature. The reaction mixture was diluted with water, extracted with $CHCl_3$, washed with water, and dried over Na_2SO_4. The organic layer was evaporated under reduced pressure, the residue was purified by column chromatography (silicagel, $CHCl_3$/AcMe 5:1) to give a red powder (0.849 g, 66%). The product is a mixture of isomers (7-*t*-Bu/6-*t*-Bu) in a ratio of ≈ 10:1.

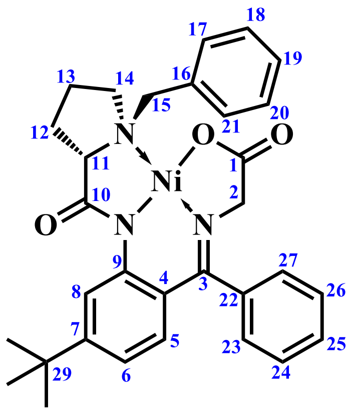

$[\alpha]_D^{26}$ = 1498 (MeOH).

HRMS (ESI): m/z 554.1952 (M+H$^+$, 554.1948 calculated for $C_{31}H_{34}N_3NiO_3$); 576.1775 (M+Na$^+$, 576.1768 calculated for $C_{31}H_{33}N_3NiO_3Na$); 592.1512 (M+K$^+$, 592.1507 calculated for $C_{31}H_{33}N_3NiO_3K$).

^{1}H NMR ($CDCl_3$ δ, ppm): 8.33 (d, ^4J = 1.8 Hz, 1H), 8.13 – 8.10 (m, 2H), 7.54 – 7.44 (m, 4H), 7.45 – 7.39 (m, 2H), 7.12 – 7.08 (m, 1H), 6.98 – 6.94 (m, 1H), 6.75 (dd, ^3J = 8.6, ^4J = 1.8 Hz, 1H), 6.71 (d, ^3J = 8.6 Hz, 1H), 4.49 (d, ^2J = 12.6 Hz, 1H), 3.77 (d, ^2J = 20.1 Hz, 1H), 3.71 – 3.65 (m, 1H), 3.67 (d, ^2J = 20.1 Hz, 1H), 3.66 (d, ^2J = 12.6 Hz, 1H), 3.49 (dd, ^3J = 10.9, 5.9 Hz, 1H), 3.44 – 3.31 (m, 1H), 2.64 – 2.55 (m, 1H), 2.49 – 2.35 (m, 1H), 2.18 – 2.02 (m, 2H), 1.23 (s, 9H).

^{13}C NMR ($CDCl_3$ δ, ppm): 181.35 (C), 177.62 (C), 171.28 (C), 155.86 (C), 142.26 (C), 134.76 (C), 133.56 (C), 132.93 (CH), 131.84 (2CH), 129.74 (CH), 129.57 (CH), 129.29 (CH), 129.11 (CH), 128.96 (2CH), 126.41 (CH), 125.77(CH), 123.01 (C), 121.43 ((CH), 118.71(CH), 70.13 (CH), 63.30 (CH_2), 61.15 (CH_2), 57.59 (CH_2), 35.19(C), 30.87 (CH_3), 30.73 (CH_2), 23.77 (CH_2).

*2.4. Complex **(SerNi)$_{L7}$***

In a manner similar to [9], a three-necked 250 mL round-bottomed flask equipped with a rubber septum with two electrodes (a glassy carbon anode, 15 cm^2, and a platinum plate, 10 cm^2) was filled with a solution of KOH (4.5 g, 80 mmol) in 150 mL of methanol and connected to a Schlenk line. The solution was deaerated by a freeze-pump-thaw cycling method. Afterwards, complex **(GlyNi)$_{L7}$** (1.5 g, 2.7 mmol) was added. The 70 mA current (corresponding to a current density of 5 mA/cm^2) was passed through the solution during 3 h with vigorous stirring. Afterwards, glacial acetic acid (7.5 mL, 131 mmol) was added and the mixture was concentrated in vacuum to 1/3 of the initial volume. Then the reaction mixture was diluted with water, extracted with chloroform, washed with $NaHCO_3$ and dried over Na_2SO_4. Evaporation of the chloroform solution yields red powder (1.55 g, 98%, dr 10:1 (*S*,2*R*):(*S*,2*S*)). The compound obtained is pure enough for the majority of applications. To obtain analytically pure sample, the complex was further purified by column chromatography (silicagel, $CHCl_3$/AcMe 5:1). The product is a mixture of isomers (7-*t*-Bu/6-*t*-Bu) in a ratio of ≈ 10:1.

(2*R*)-(SerNi)$_{L7}$

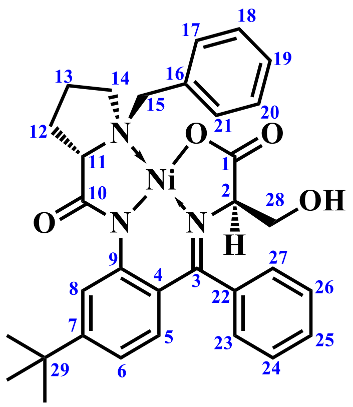

HRMS (ESI): m/z 584.2057 (M+H$^+$, 584.2054 calculated for $C_{32}H_{36}N_3NiO_4$); 606.1874 (M+Na$^+$, 606.1873 calculated for $C_{32}H_{35}N_3NiO_4Na$).

^{1}H NMR ($CDCl_3$ δ, ppm): 8.59 (d, ^4J = 1.9 Hz, 1H), 8.14 – 8.10 (m, 2H), 7.52 – 7.40 (m, 6H), 7.18 – 7.13 (m, 1H), 7.11 – 7.06 (m, 1H),

6.76 (dd, ^3J = 8.7, ^3J = 1.9 Hz, 1H), 6.71 (d, ^3J = 8.7 Hz, 1H), 4.43 (d, ^2J = 12.6 Hz, 1H), 4.10 – 4.02(m, 1H), 3.89 (dd, ^3J = 6.9, 4.5 Hz, 1H), 3.74 – 3.67 (m, 1H), 3.62 – 3.54 (m, 3H), 3.15 (br. s., 1H), 2.70 – 2.58 (m, 1H), 2.58 – 2.49 (m, 1H), 2.35 – 2.25 (m, 1H), 2.25 – 2.15 (m, 1H), 1.97-1.87 (m, 1H),1.23 (s, 9H).

^{13}C NMR ($CDCl_3$ δ, ppm): 182.30 (C), 179.88 (C), 172.53 (C), 156.50 (C), 142.74 (C), 134.19 (C), 133.78 (C), 133.62 (CH), 131.74 (2CH), 129.80 (CH), 129.28 (CH), 129.26 (CH), 129.17 (2CH), 128.71 (CH), 128.45 (CH), 126.56 (CH), 123.48 (C), 121.03 (CH), 118.75 (CH), 71.24 (CH), 69.47 (CH), 64.73 (CH_2), 62.37 (CH_2), 58.66 (CH_2), 35.26 (C), 30.86 ($3CH_3$), 23.37 (CH_2).

(2*S*)-(SerNi)$_{L7}$

HRMS (ESI): m/z 584.2055 (M+H$^+$, 584.2054 calculated for $C_{32}H_{36}N_3NiO_4$); 606.1871 (M+Na$^+$, 606.1873 calculated for $C_{32}H_{35}N_3NiO_4Na$).

^{1}H NMR ($CDCl_3$ δ, ppm): 8.20 – 8.13 (m, 3H), 7.54 – 7.40 (m, 3H), 7.40 – 7.33 (m, 2H), 7.25 – 7.21 (m, 1H), 7.20 – 7.15 (m, 1H), 6,99 – 6,95 (m, 1H), 6.69 (dd, ^3J = 8.7, ^4J = 1.9 Hz, 1H), 6.54 (d, ^3J = 8.6 Hz, 1H), 4.35 (d, ^2J = 12.6 Hz, 1H), 3.99 – 3.94 (m, 1H), 3.86 – 3.72 (m, 3H), 3.51 – 3.43 (m, 3H), 2.96 (s, 1H), 2.76 – 2.67 (m, 1H), 2.54 – 2.40 (m, 1H), 2.16 – 2.06 (m, 1H), 2.06 – 1.97 (m, 1H), 1.21 (s, 9H).

*2.5. Complex **(ΔAlaNi)$_{L7}$***

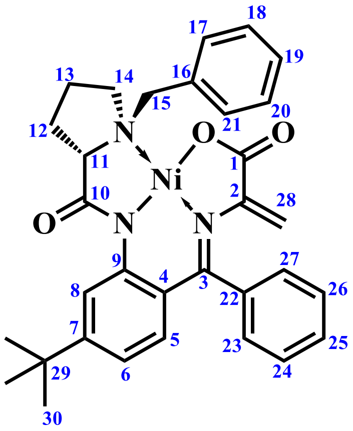

In a manner similar to [10], to the solution of **(SerNi)$_{L7}$** (2 g, 3.4 mmol) in MeCN (6 mL), Na_2CO_3 (1.73 g, 16.3 mmol), acetic anhydride (2.45 mL, 26 mmol) and acetic acid (1.49 mL, 26 mmol) were added. The solution was deaerated by a freeze-pump-thaw cycling method. The stirring was continued at 65 °C until complete conversion of the initial complex (8.5 h), as monitored by TLC (silicagel, $CHCl_3$/AcMe 5:1). The mixture was cooled, filtered, the precipitate was washed with $CHCl_3$, the filtrate and washings were combined and evaporated under reduced pressure; the residue was purified by column chromatography (silicagel, $CHCl_3$/AcMe 5:1) to give a red powder (1.79 g, 92%). The product is a mixture of isomers (7-*t*-Bu/6-*t*-Bu) in a ratio of ≈ 10:1.

$[\alpha]_D^{20}$ = 2127 (MeOH).

HRMS (ESI): m/z 566.1949 (M+H$^+$, 566.1948 calculated for $C_{32}H_{34}N_3NiO_3$); 588.1760 (M+Na$^+$, 588.1768 calculated for $C_{32}H_{33}N_3NiO_3Na$).

^{1}H NMR ($CDCl_3$ δ, ppm): 8.22 – 8.18 (m, 2H (H-17,21)), 8.14 (dd, ^4J = 1.8, ^5J = 0.6 Hz, 1H (H-8)), 7.50 – 7.36 (m, 4H (H-24,25,26,27)), 7.37 – 7.30 (m, 2H (H-18,20)), 7.18 – 7.00 (m, 2H (H-19,23)), 6.74 (dd, ^3J = 8.7, ^5J = 0.6 Hz, 1H, (H-5)), 6.71 (dd, ^3J = 8.7, ^4J = 1.8 Hz, 1H, (H-6)), 5.59 (d, ^2J = 1.2 Hz, 1H (H-28)), 4.33 (d, ^2J = 12.6 Hz, 1H (H-15)), 4.10 (d, ^2J = 1.2 Hz, 1H (H-28)), 3.83 – 3.63 (m, 1H (H-13)), 3.60 – 3.52 (m, 1H (H-14), 3.52-3.44 (m, 1H (H-11)), 3.36 (d, ^2J = 12.6 Hz, 1H (H-15)), 2.71 – 2.60 (m, 1H (H-12)), 2.59 – 2.45 (m, 1H (H-12)), 2.22 – 2.12 (m, 1H (H-13)), 2.12 – 2.01 (m, 1H (H-14)), 1.22 (s, 9H (H-30)).

^{13}C NMR ($CDCl_3$ δ, ppm): 180.57 (C-10), 170.97 (C-1), 168.69 (C-3), 156.78 (C-7), 146.84 (C-2), 143.25 (C-9), 135.45 (C-22), 133.99 (C-16), 133.92 (C-5), 131.50 (C-17,21), 129.96 (C-25), 128.98 (C-18,20), 128.92 (C-19), 128.7 (br.s., 3CH), 127.8 (br.s., CH), 124.82 (C-4), 120.55 (C-8), 118.79

(C-6), 114.78 (C-28), 70.80 (C-11), 63.39 (C-15), 57.97 (C-14), 35.25 (C-29), 30.84 (C-12), 30.79 (C-30), 24.04 (C-13).

*2.6. General procedure for synthesis of complexes **(^{R}CysNi)$_{L7}$***

In a manner similar to [10], to a solution of **(ΔAlaNi)$_{L7}$** (50 mg, 0.088 mmol) in CH_3CN (0.14 mL), K_2CO_3 (24 mg, 0.174 mmol) and ArSH (0.092 mmol) were added under argon. The mixture was stirred at 50–55 °C until complete conversion of the initial complex (2–4 h), as monitored by TLC (silicagel, $CHCl_3$/AcMe 5:1). Afterwards, the mixture was diluted with water, extracted with $CHCl_3$, dried over Na_2SO_4 and evaporated under reduced pressure. The residue was purified by column chromatography (silicagel, $CHCl_3$/EtOAc 5:1). The products are mixtures of isomers (7-*t*-Bu/6-*t*-Bu) in a ratio of ≈ 10:1.

*2.7. Complex **(^{Bn}CysNi)$_{L7}$***

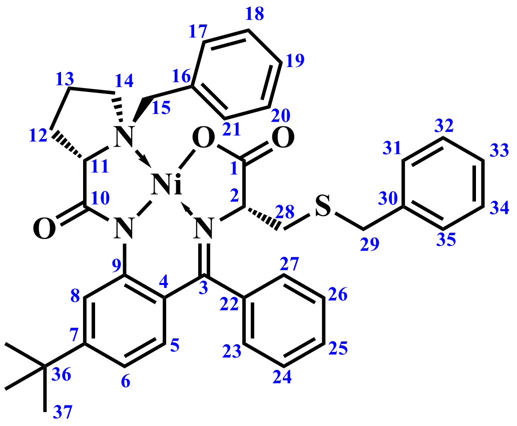

Complex **(^{Bn}CysNi)$_{L7}$**: red powder, 52 mg, yield 85%, dr >52:1.

$[\alpha]_D^{26}$ = 665 (MeOH)

HRMS (ESI): 690.2255 (M+H$^+$, 690.2295 calculated for $C_{39}H_{42}N_3NiO_3S$), 712.2071 (M+Na$^+$, 712.2114 calculated for $C_{39}H_{41}N_3NiO_3SNa$).

^{1}H NMR ($CDCl_3$ δ, ppm): 8.30 (d, ^4J = 2.0 Hz, 1H (H-8)), 8.18 – 8.11 (m, 2H (H-17,21)), 7.47 – 7.14 (m, 12H (H-18,19,20,24,25,26,27,31,32,33,34,35), 6.67 (dd, ^3J = 8.7, ^4J = 2.0 Hz, 1H (H-6)), 6.58 – 6.53 (m, 1H (H-23)), 6.48 (d, ^3J = 8.7 Hz, 1H (H-5)), 4.46 (d, ^2J = 12.6 Hz, 1H (H-15)), 4.15 (dd, ^3J = 5.4, 2.7 Hz, 1H (H-2)), 3.90 (d, ^2J = 13.5 Hz, 1H (H-29)), 3.81 (d, ^2J = 13.5 Hz, 1H (H-29)), 3.77 – 3.61 (m, 2H (H-13,14)), 3.56 (d, ^2J = 12.6 Hz, 1H (H-15)), 3.48 (dd, ^3J = 10.5, 6.4 Hz, 1H (H-11)), 2.91 – 2.80 (m, 1H (H-12)), 2.74 (dd, ^2J = 13.4, ^3J = 2.7 Hz, 1H (H-28)), 2.54 – 2.42 (m, 1H (H-12)), 2.39 (dd, ^2J = 13.4, ^3J = 5.4 Hz, 1H (H-28)), 2.14 – 1.94 (m, 2H (H-13,14)), 1.23 (s, 9H (H-37)).

^{13}C NMR ($CDCl_3$ δ, ppm): 180.71 (C-10), 178.72 (C-1), 171.11 (C-3), 156.06 (C-7), 142.60 (C-9), 137.72 (C-30), 133.16 (C-5), 131.77 (C-17,21), 134.02, 133.76, 129.66, 129.28, 128.98, 128.94, 128.87, 128.60, 127.23, 126.85 (C-16,18,19,20,22,24,25,26,27,32,33,34), 128.91 (C-31,35), 127.58 (C-23), 123.99 (C-4), 120.74 (C-8), 118.47 (C-6), 70.91 (C-11), 69.13 (C-2), 63.71 (C-15), 57.60 (C-14), 37.56 (C-29), 35.22 (C-36), 34.24 (C-28), 30.93 (C-12), 30.89 (C-37), 23.32 (C-13).

*2.8. Complex **(^{Ph}CysNi)$_{L7}$***

Complex **(^{Ph}CysNi)$_{L7}$**: red powder, 44 mg, yield 73%, dr >44:1.

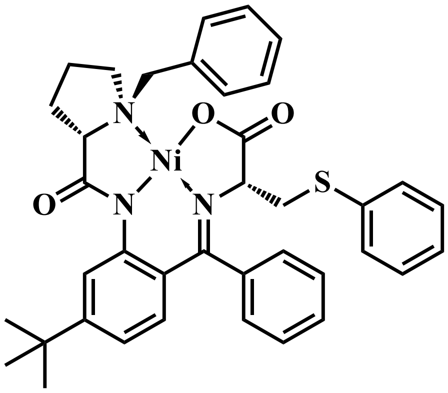

$[\alpha]_D^{26}$ = 1171 (MeOH).

HRMS (ESI): 676.2099 (M+H$^+$, 676.2138 calculated for $C_{38}H_{40}N_3NiO_3S$), 698.1914 (M+Na$^+$, 698.1958 calculated for $C_{38}H_{39}N_3NiO_3SNa$), 714,1650 (M+K$^+$, 714.1697 calculated for $C_{38}H_{39}N_3NiO_3SK$).

^{1}H NMR ($CDCl_3$ δ, ppm): 8.29 (m, ^4J = 2.0 Hz, 1H), 8.17 – 8.13 (m, 2H), 7.50 – 7.38 (m, 4H), 7.38 – 7.33 (m, 2H), 7.33

– 7.28 (m, 1H), 7.23 – 7.14 (m, 5H), 6.68 (dd, ^3J = 8.7, ^4J = 2.0 Hz, 1H), 6.61 – 6.57 (m, 1H), 6.49 (d, ^3J = 8.7 Hz, 1H), 4.45 (d, ^2J = 12.6 Hz, 1H), 4.27 (dd, ^3J = 6.1, 2.6 Hz, 1H), 3.80 – 3.65 (m, 1H), 3.65 – 3.58 (m, 1H), 3.55 (d, ^2J = 12.6 Hz, 1H), 3.47 (dd, ^3J = 6.4, 10.5 Hz, 1H), 3.27 (dd, ^2J = 13.1, ^3J = 2.6 Hz, 1H), 2.91 (dd, ^2J = 13.1, ^3J = 6.1 Hz, 1H), 2.91-2.82 (m, 1H), 2.58 – 2.43 (m, 1H), 2.12 – 1.96 (m, 2H), 1.23 (s, 9H).

^{13}C NMR ($CDCl_3$ δ, ppm): 180.66 (C), 178.53 (C), 171.38 (C), 156.13 (C), 142.67 (C), 135.93 (C), 134.09 (C), 133.71 (C), 133.15 (CH), 131.77 (2CH), 131.02 (2CH), 129.83 (CH), 129.21 (2CH), 128.96 (2CH), 128.92 (CH), 128.89 (2CH), 127.58 (CH), 127.02 (CH), 126.88 (CH), 124.01 (C), 120.82 (CH), 118.50 (CH), 70.92 (CH), 69.08 (CH), 63.66 (CH_2), 57.60 (CH_2), 38.67 (CH_2), 35.23 (C), 30.93 (CH_2), 30.89 ($3CH_3$), 23.34 (CH_2).

*2.9. Complex (**^{pMe}CysNi)$_{L7}$***

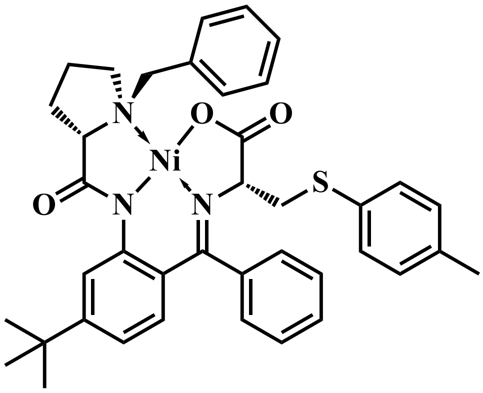

Complex **(^{pMe}CysNi)$_{L7}$** was obtained from 42 mg of initial complex **(ΔAlaNi)$_{L7}$**: red powder, 43 mg, yield 83%, dr >43:1, 3% of the initial complex did not react.

$[\alpha]_D^{26}$ = 1510 (MeOH).

HRMS (ESI): m/z 690.2293 (M+H$^+$, 690.2295 calculated for $C_{39}H_{42}N_3NiO_3S$).

^{1}H NMR ($CDCl_3$ δ, ppm): 8.30 (d, ^4J = 2.0 Hz, 1H), 8.17 – 8.12 (m, 2H), 7.49 – 7.40 (m, 2H), 7.40 – 7.28 (m, 5H), 7.22 – 7.14 (m, 2H), 7.03 – 6.98 (m, 2H), 6.68 (dd, ^3J = 8.7, ^4J = 2.0 Hz, 1H), 6.65 – 6.60 (m, 1H), 6.49 (d, ^3J = 8.7 Hz, 1H), 4.46 (d, ^2J = 12.6 Hz, 1H), 4.24 (dd, ^3J = 6.1, 2.6 Hz, 1H), 3.81 – 3.67 (m, 1H), 3.67 – 3.60 (m, 1H), 3.56 (d, ^2J = 12.6 Hz, 1H), 3.51 – 3.43 (m, 1H), 3.21 (dd, ^2J = 13.1, ^3J = 2.6 Hz, 1H), 2.92– 2.84 (m, 1H), 2.88 (dd, ^2J = 13.1, ^3J = 6.1 Hz, 1H), 2.56 – 2.44 (m, 1H), 2.26 (s, 3H), 2.12 – 1.96 (m, 2H), 1.24 (s, 9H).

^{13}C NMR ($CDCl_3$ δ, ppm): 180.68 (C), 178.60 (C), 171.31 (C), 156.03 (C), 142.54 (C), 137.25 (C), 134.04 (C), 133.74 (C), 133.10 (CH), 132.13 (C), 131.72 (3CH), 129.92 (2CH), 129.75 (CH), 128.90 (2CH) 128.87 (CH), 128.84 (3CH), 127.53 (CH), 126.82 (CH), 123.99 (C), 120.76 (CH), 118.48 (CH), 70.94 (CH), 69.18 (CH), 63.67 (CH_2), 57.63 (CH_2), 39.30 (CH_2), 35.17 (C), 30.89 (CH_2), 30.84 ($3CH_3$), 23.30 (CH_2), 21.07 (CH_3).

*2.10. Complex (**^{oBr}CysNi)$_{L7}$***

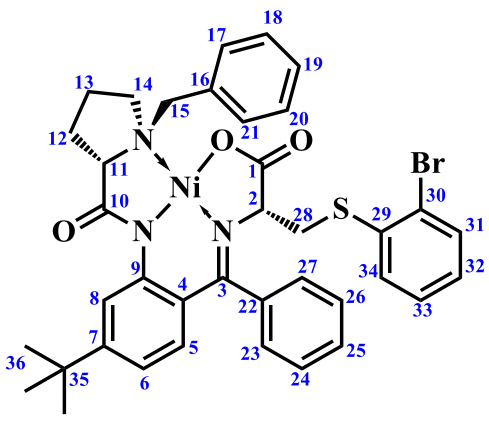

Complex **(^{oBr}CysNi)$_{L7}$** was obtained from 30 mg of initial complex **(ΔAlaNi)$_{L7}$**: red powder, 29 mg, yield 71%, dr >29:1.

$[\alpha]_D^{26}$ = 918 (MeOH).

HRMS (ESI): m/z 754.1242 (M+H$^+$, 754.1243 calculated for $C_{38}H_{39}BrN_3NiO_3S$).

^{1}H NMR ($CDCl_3$ δ, ppm): 8.27 (d, ^4J = 2.0 Hz, 1H (H-8)), 8.16 – 8.12 (m, 2H (H-17,21)), 7.54 (dd, ^3J = 7.9, ^4J = 1.5 Hz, 1H (H-31)), 7.50 – 7.41 (m, 2H (H-25,26)), 7.39 – 7.32 (m, 3H (H-18,20,24)), 7.25 – 7.21 (m, 1H (H-27)), 7.20 –

7.16 (m, 2H (H-19, 34)), 7.13 (td, ^3J = 7.9, ^4J = 1.5 Hz, 1H (H-33)), 7.00 (ddd, ^3J = 7.9, 7.2, ^4J = 1.8 Hz, 1H (H-32)), 6.83 – 6.79 (m, 1H (H-23)), 6.68 (dd, ^3J = 8.7, ^4J = 2.0 Hz, 1H (H-6)), 6.52 (d, ^3J = 8.7 Hz, 1H (H-5)), 4.42 (d, ^2J = 12.6 Hz, 1H (H-15)), 4.34 (dd, ^3J = 6.0, 3.0 Hz, 1H (H-2)), 3.74 – 3.67 (m, 1H (H-13)), 3.62 – 3.52 (m, 2H (H-14,15)), 3.46 (dd, ^2J = 10.5, ^3J = 6.6 Hz, 1H (H-11)), 3.28 (dd, ^2J = 12.6, ^3J = 3.0 Hz, 1H (H-28)), 2.95 – 2.89 (m, 2H (H-12,28)), 2.58 – 2.46 (m, 1H (H-12)), 2.12 – 1.97 (m, 2H (H-13,14)), 1.23 (s, 9H (H-36)).

^{13}C NMR ($CDCl_3$ δ, ppm): 180.66 (C-10), 178.41 (C-1), 171.68 (C-3), 156.28 (C-7), 142.76 (C-9), 137.15 (C-29), 134.04 (C-22), 133.71 (C-16), 133.26 (C-5), 133.12 (C-31), 131.76 (C-17,21), 129.88 (C-25), 129.03 (C- 19), 128.96 (C-24 or26), 128.94 (C-24 or 26), 128.92 (C-18,20), 128.76 (C-34), 128.09 (C-33), 127.75 (C-23), 127.29 (C-32), 126.84 (C-27), 124.19 (C-30), 124.03 (C-4), 120.84 (C-8), 118.57 (C-6), 71.01 (C-11), 68.33 (C-2), 63.68 (C-15), 57.69 (C-14), 36.59 (C-28), 35.25 (C-35), 31.09 (C-12), 30.88 (C-36), 23.55 (C-13).

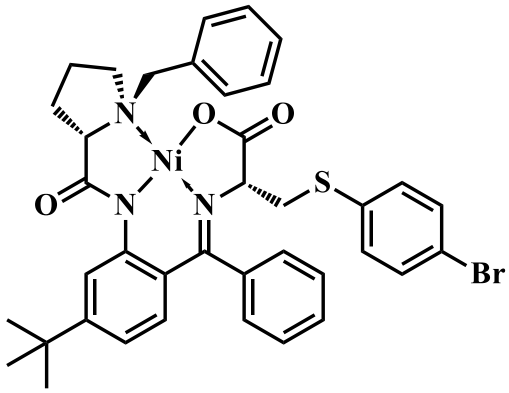

2.11. *Complex (**^{pBr}CysNi)$_{L7}$***

Complex **(^{pBr}CysNi)$_{L7}$** was obtained from 31 mg of initial complex **(ΔAlaNi)$_{L7}$**: red powder, 32 mg, yield 78%, dr = 32:1, 20% of the initial complex did not react.

$[\alpha]_D^{26}$ = 1166 (MeOH).

HRMS (ESI): m/z 754.1241 ($M+H^+$, 754.1243 calculated for $C_{38}H_{39}BrN_3NiO_3S$).

^{1}H NMR ($CDCl_3$ δ, ppm): 8.27 (d, ^4J = 1.9 Hz, 1H), 8.16 – 8.12 (m, 2H), 7.48 – 7.45 (m, 2H), 7.38 – 7.31 (m, 5H), 7.25 – 7.19 (m, 3H), 7.19 – 7.14 (m, 1H), 6.75 – 6.70 (m, 1H), 6.69 (dd, ^3J = 8.7, ^4J = 1.9 Hz, 1H), 6.50 (d, ^3J = 8.7 Hz, 1H), 4.43 (d, ^2J = 12.6 Hz, 1H), 4.24 (dd, ^3J = 6.4, 2.8 Hz, 1H), 3.78 – 3.54 (m, 2H), 3.55 (d, ^2J = 12.6 Hz, 1H), 3.48 (dd, J = 10.6, 6.5 Hz, 1H), 3.21 (dd, ^2J = 13.1, ^3J = 2.8 Hz, 1H), 2.96 (dd, ^2J = 13.1, ^3J = 6.4 Hz, 1H), 2.90 – 2.79 (m, 1H), 2.56 – 2.44 (m, 1H), 2.11 – 1.98 (m, 2H), 1.23 (s, 9H).

^{13}C NMR ($CDCl_3$ δ, ppm): 180.65 (C), 178.28 (C), 171.46 (C), 156.32 (C), 142.64 (C), 134.92 (C), 134.03 (C), 133.69 (C), 133.23 (CH), 132.35 (2CH), 132.25 (2CH), 131.75 (2CH), 129.94 (CH), 129.08 (CH), 129.04 (CH), 128.97 (CH), 128.93 (2CH), 127.53 (CH), 126.92 (CH), 123.95 (C), 121.06 (C), 120.82 (CH), 118.62 (CH), 70.86 (CH), 68.82 (CH), 63.69 (CH_2), 57.57 (CH_2), 38.68 (CH_2), 35.25 (C), 30.92 (CH_2), 30.88 ($3CH_3$), 23.41 (CH_2).

2.12. *L-S-tolylcysteine*

In a manner similar to [11], to a solution of complex **(^{pMe}CysNi)$_{L7}$** (0.7 g, 1 mmol) in methanol (7 mL), HCl (12 N, 0.42 mL, 5 mmol) was added, and the reaction mixture was heated at 60 °C for 7.5–8 h, then cooled to room temperature. The disappearance of the red color of the complex was observed. The solution was evaporated under reduced pressure and diluted with distilled water and diethyl ether. Ethylenediaminetetraacetic acid disodium salt dihydrate (0.561 g, 1.5 mmol) was added, and the solution was basified to pH 12–13 by aqueous solution of NaOH (12 N). The aqueous layer was washed with diethyl ether and concentrated to dryness. The white residue was diluted with a small amount of water (leaving some amount of residue undissolved) and the obtained solution was neutralized with 2 N HCl to pH 6–7 to give a

precipitate which was filtered and washed with distilled water yielding a white powder (0.088 g). The filtrate was concentrated to obtain additional amount of the amino acid (0.010 g, 49% total yield). For preparation of an NMR sample, HCl (12 N, 5 μL) was added to the suspension of the amino acid in DMSO-d_6. The organic layers were combined, washed with water, dried over Na_2SO_4 and evaporated under reduced pressure to obtain **L7** as brown oil (0.306 g, 69%).

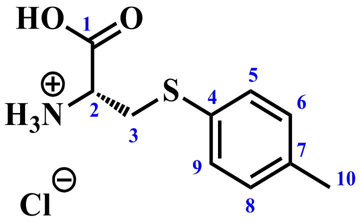

^{1}H NMR (DMSO-d_6 δ, ppm): 8.66 (s, 3H (NH)), 7.37 (m, 2H (H-5,9)), 7.18 (m, 1H (H-6,8)), 4.37 – 3.56 (overlap of the signals of H-2, aqueous HCl and protonated amino-group), 3.40 (d, ^{3}J = 6.0 Hz, 1H (H-3)), 2.28 (s, 3H (H-10)).

^{13}C NMR (DMSO-d_6 δ, ppm): 169.20 (C-1), 136.73 (C-7), 130.43 (C-5,9), 130,28 (C-4), 129.95 (C-6,8), 51.57 (C-2), 34.31 (C-3), 20.64 (C-10).

3. NMR spectra for all compounds

3.1. ^{1}H NMR spectrum of 2-benzoyl-5-tert-butylaniline

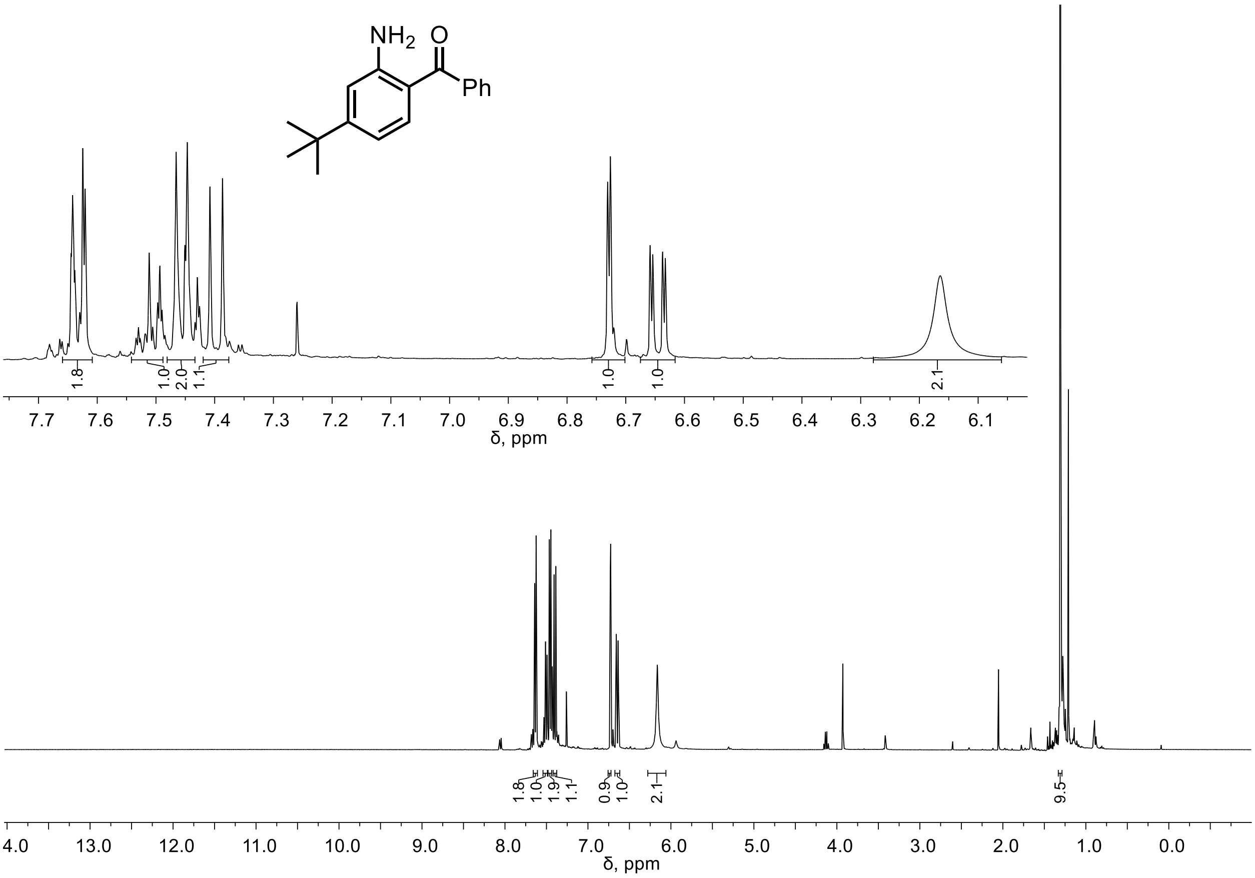

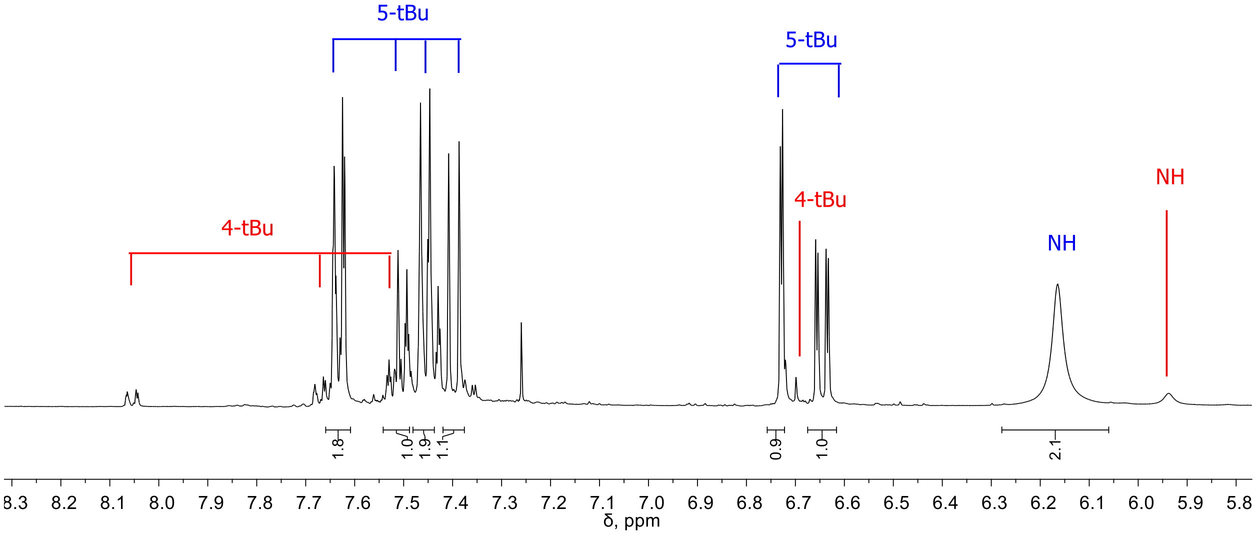

*In all NMR spectra additional signals of 2-benzoyl-4-*tert*-butylaniline are observed in the ratio ≈ 1:10 because of the incomplete migration of *t*-Bu-group. However, these signals could be identified easily, and it does not complicate analysis of NMR spectra.

3.2. ^{13}C NMR spectrum of 2-benzoyl-5-tert-butylaniline

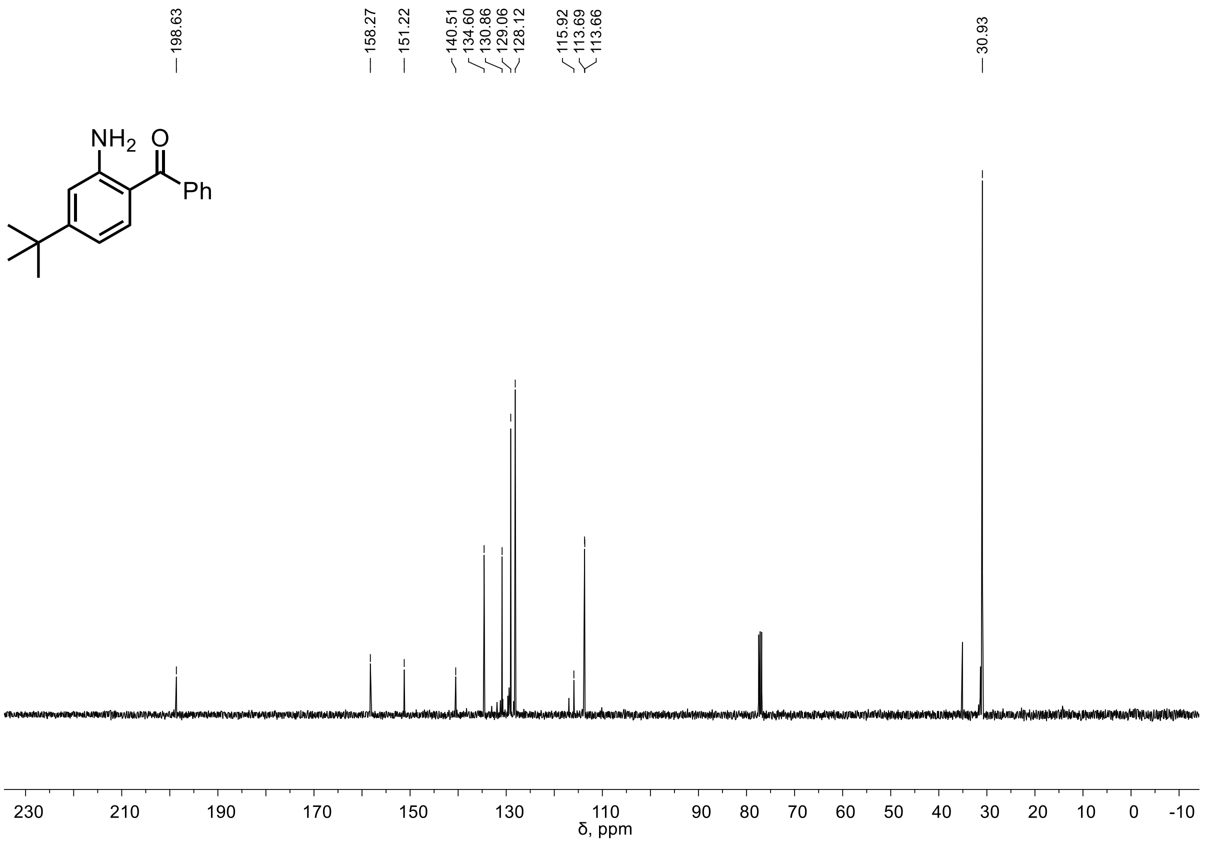

3.3. *HSQC spectrum of 2-benzoyl-5-tert-butylaniline*

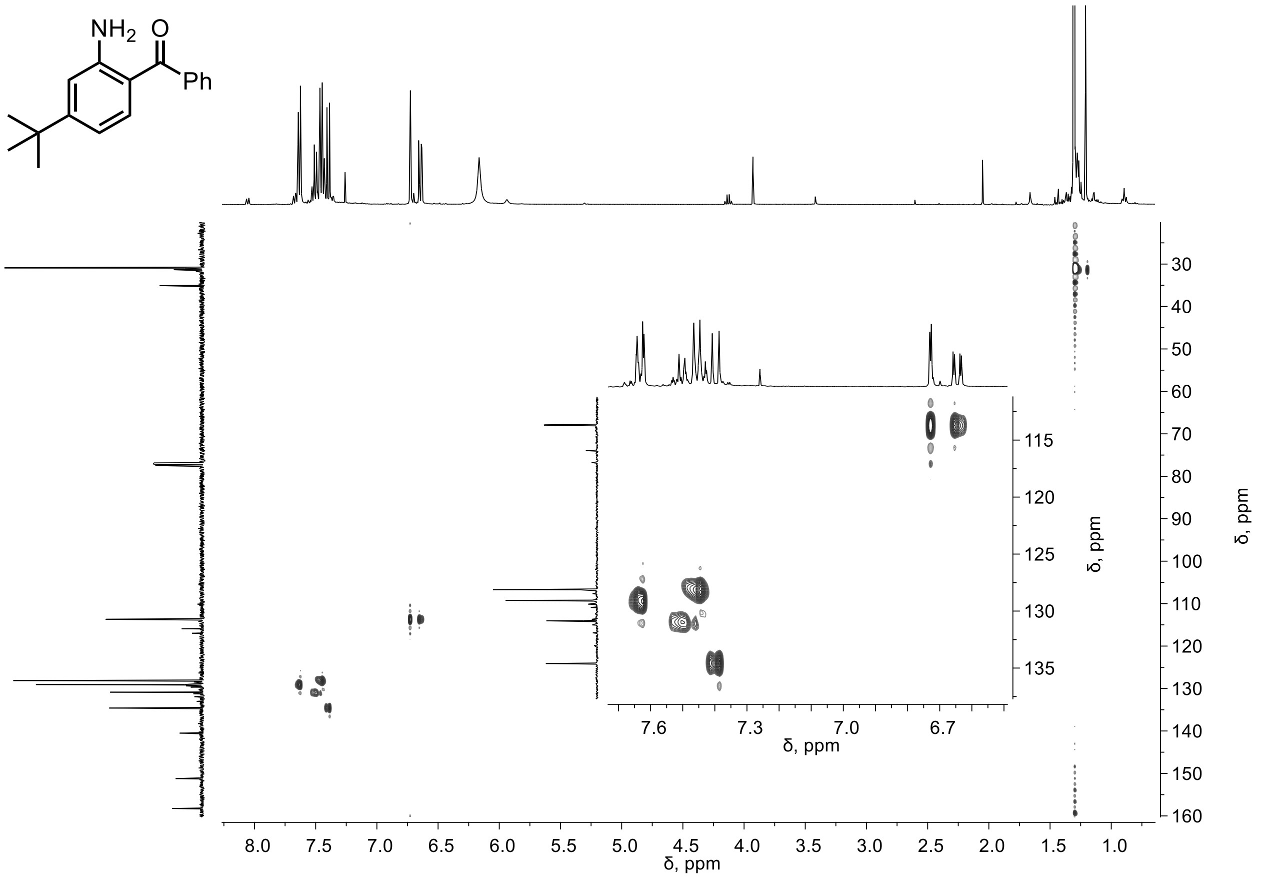

3.4. *HMBC spectrum of 2-benzoyl-5-tert-butylaniline*

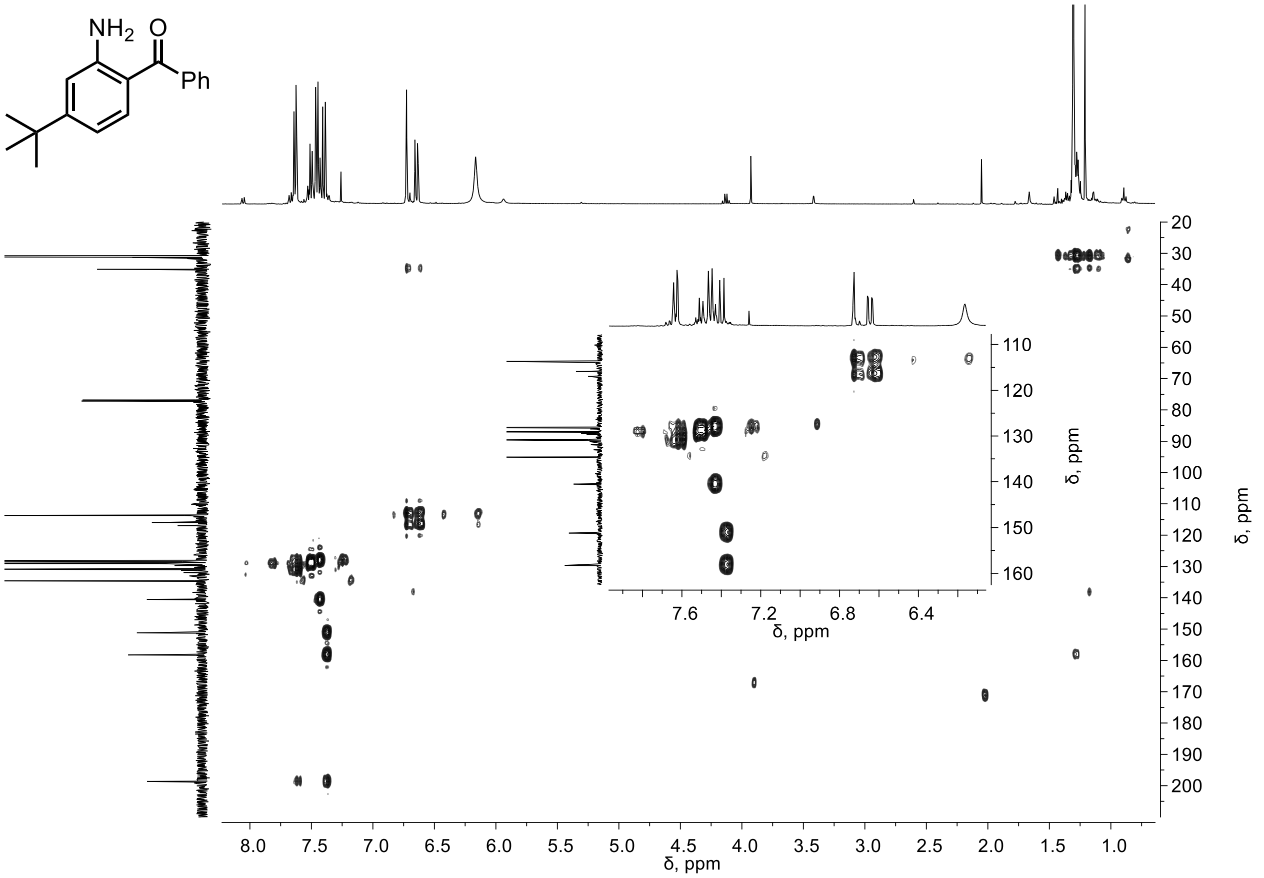

3.5. ^{1}H NMR spectrum of compound ***L7***

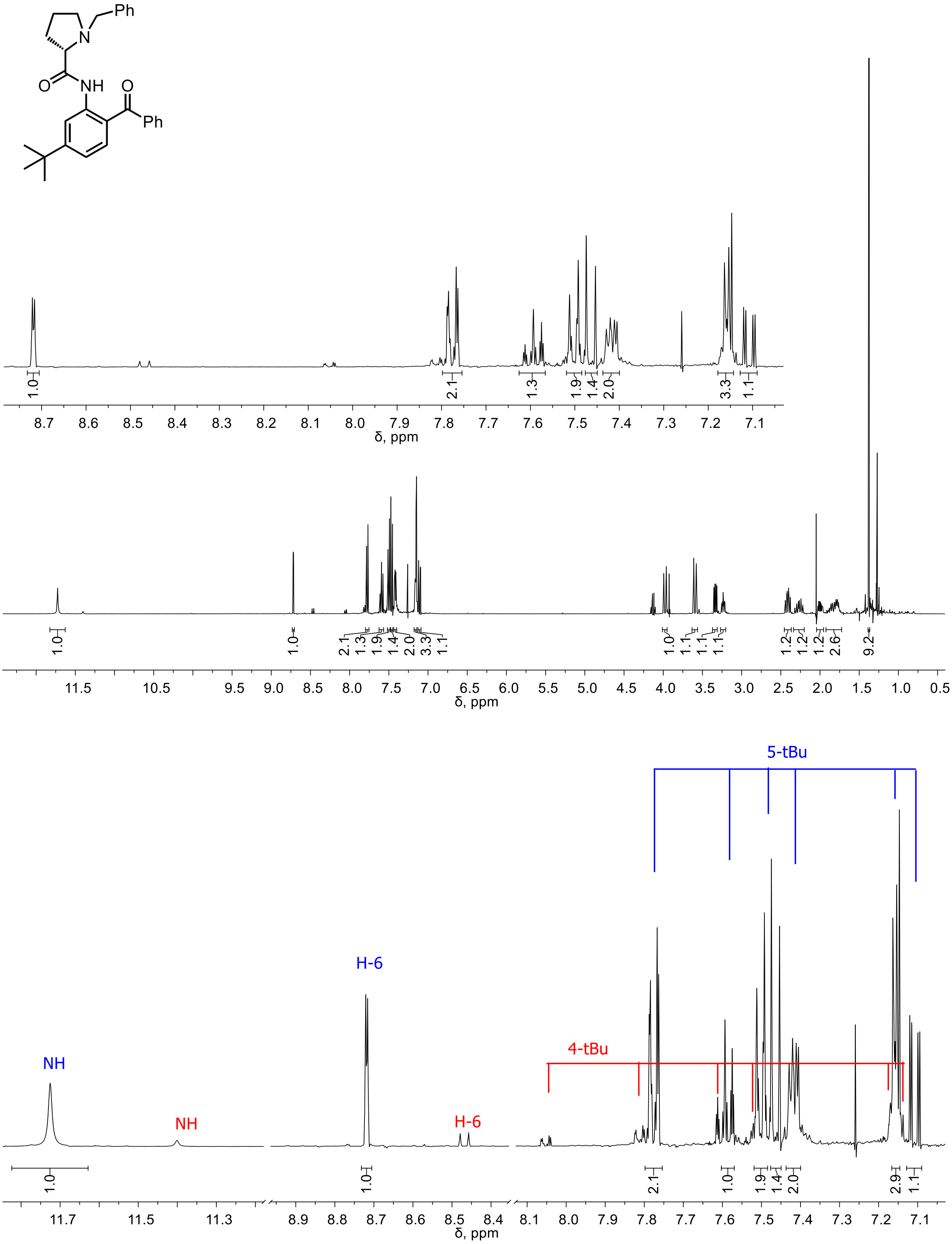

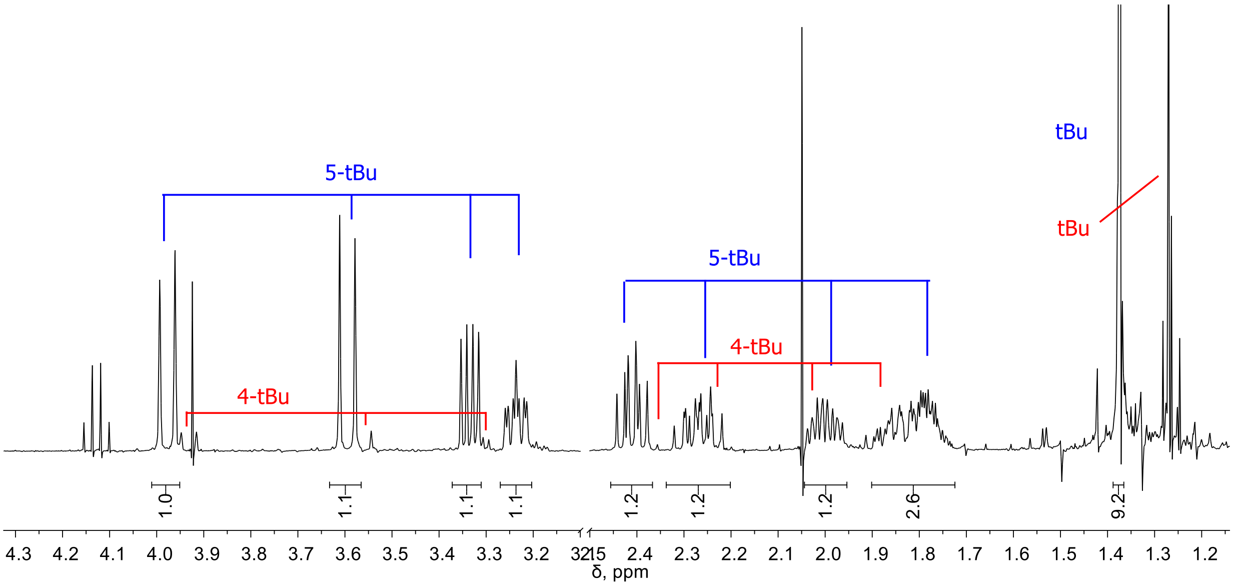

3.6. ^{13}C NMR spectrum of compound ***L7***

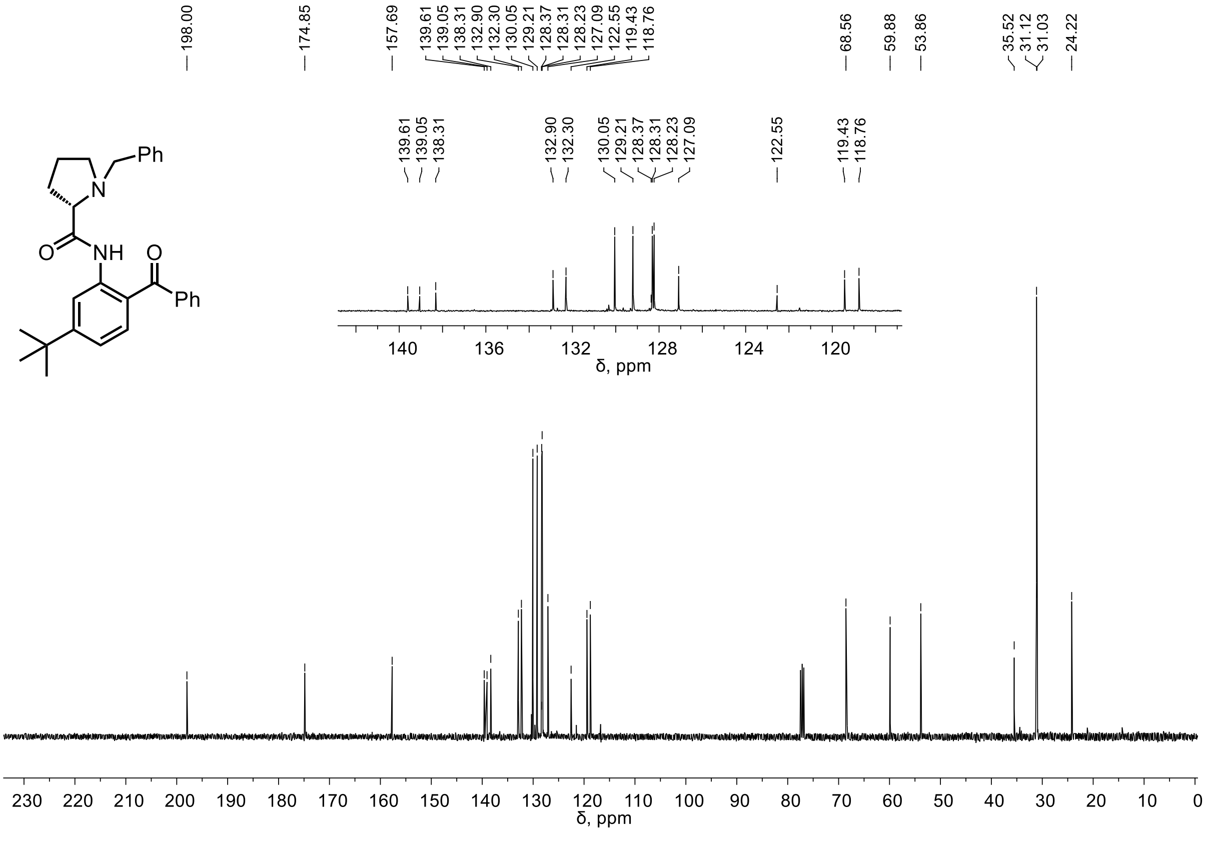

*3.7. HSQC spectrum of compound **L7***

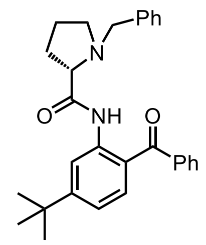

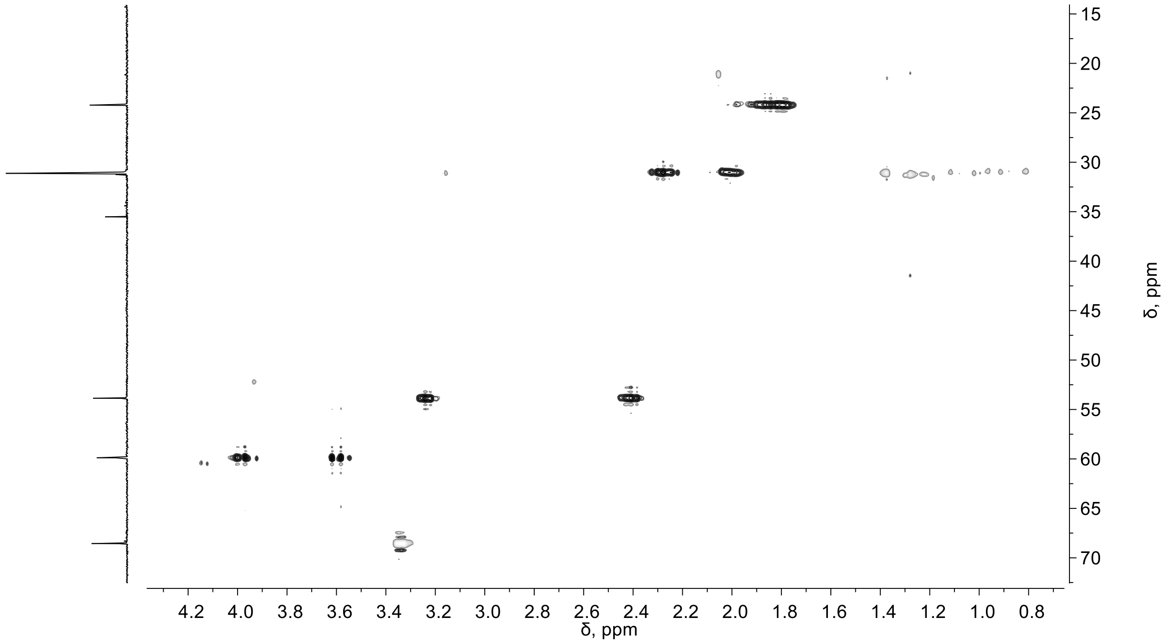

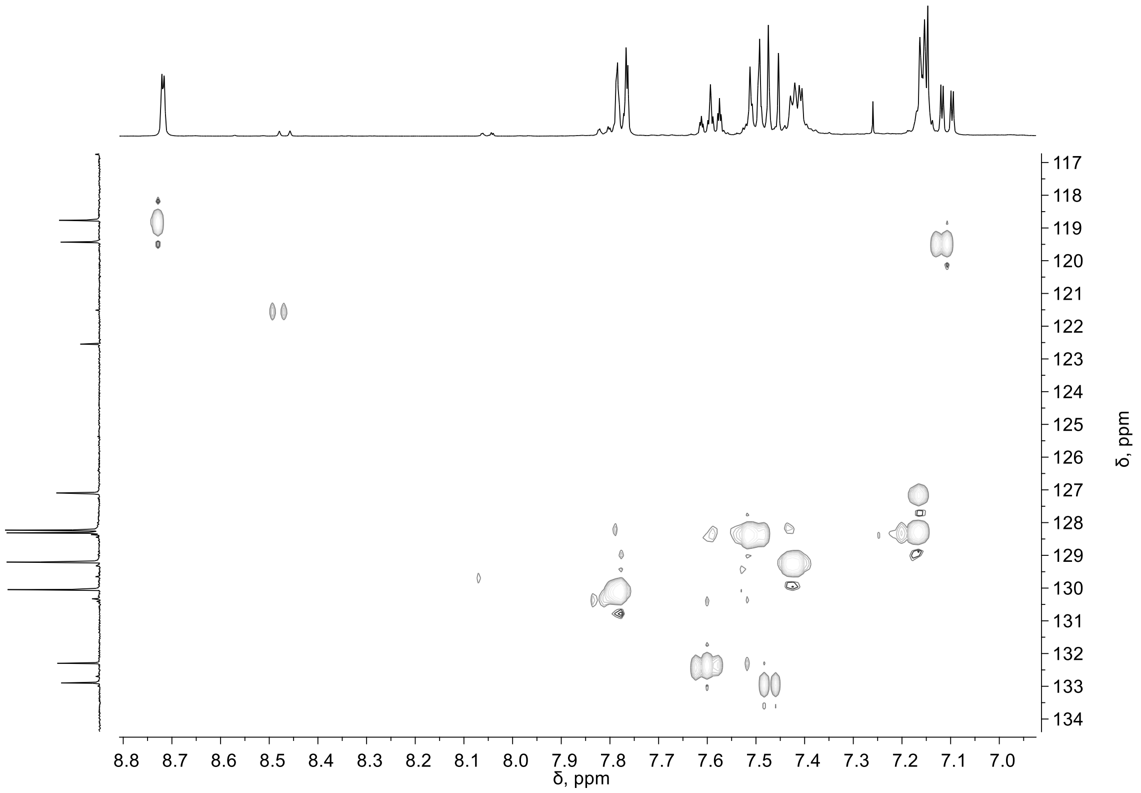

3.8. HMBC spectrum of compound **L7**

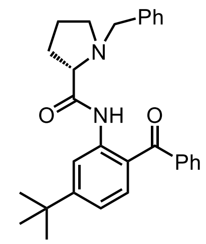

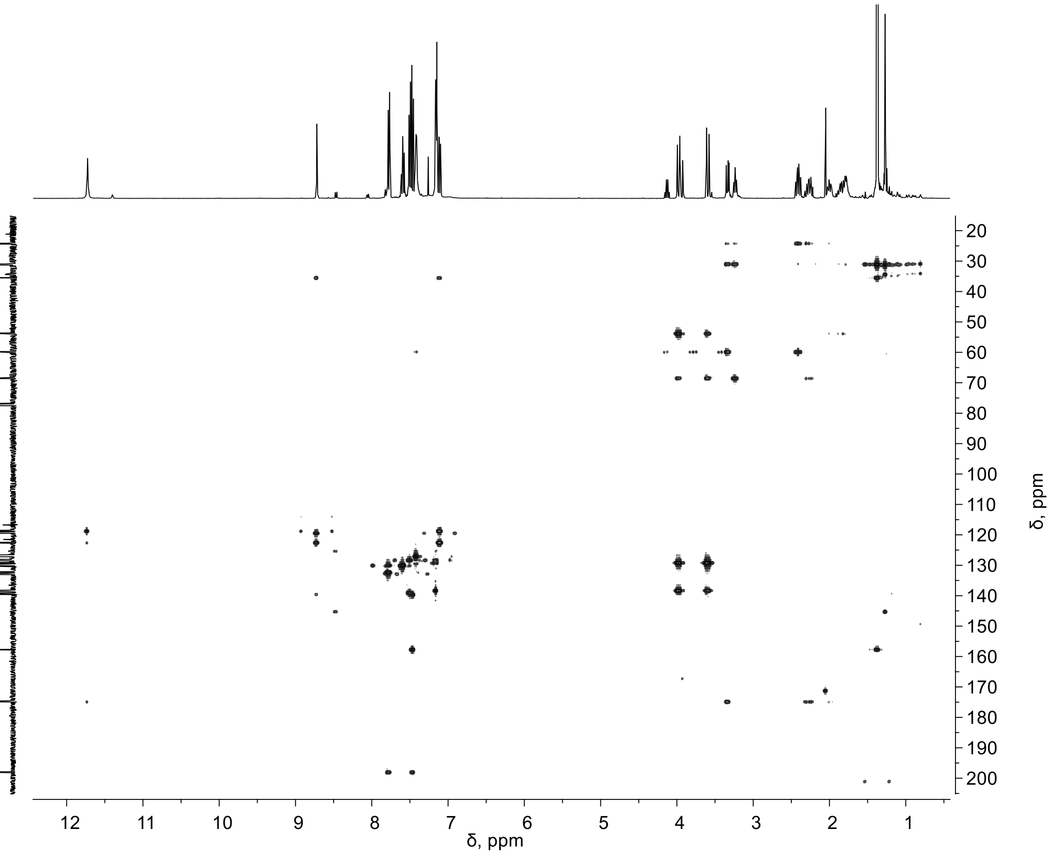

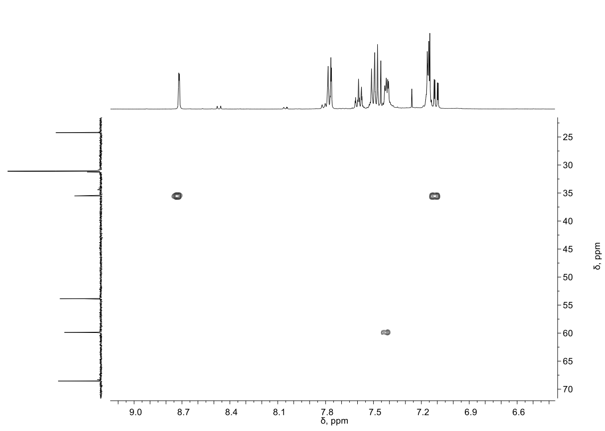

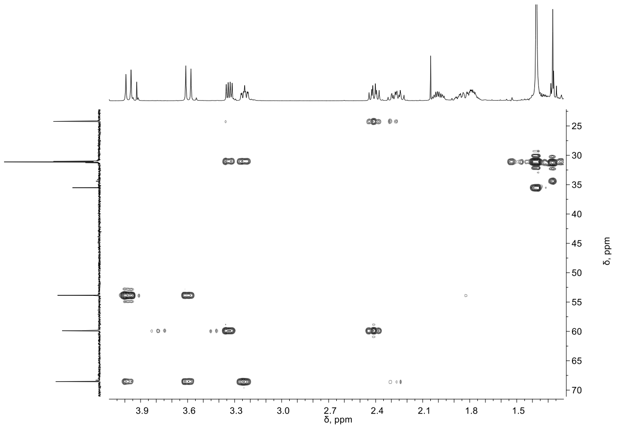

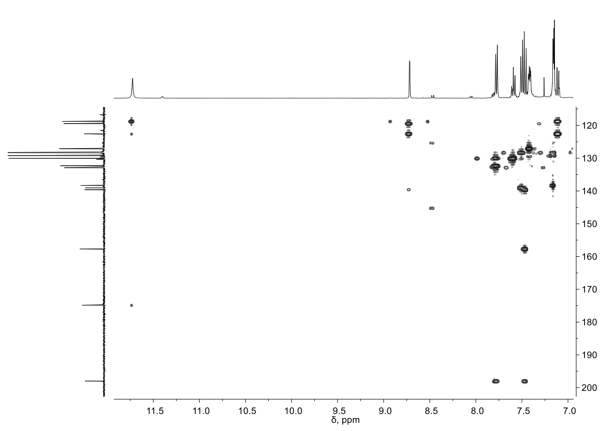

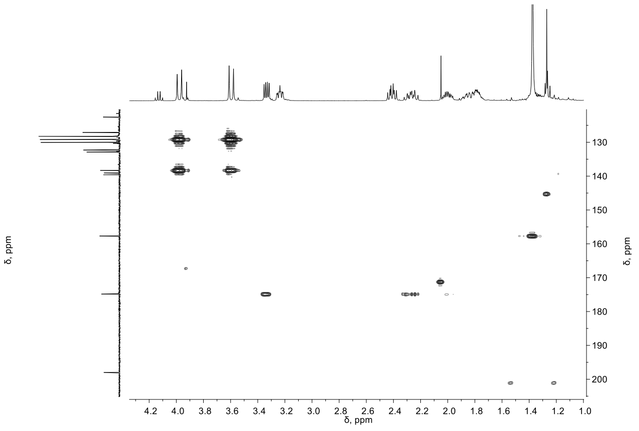

3.9. ^{1}H NMR spectrum of complex **(GlyNi)$_{L7}$**

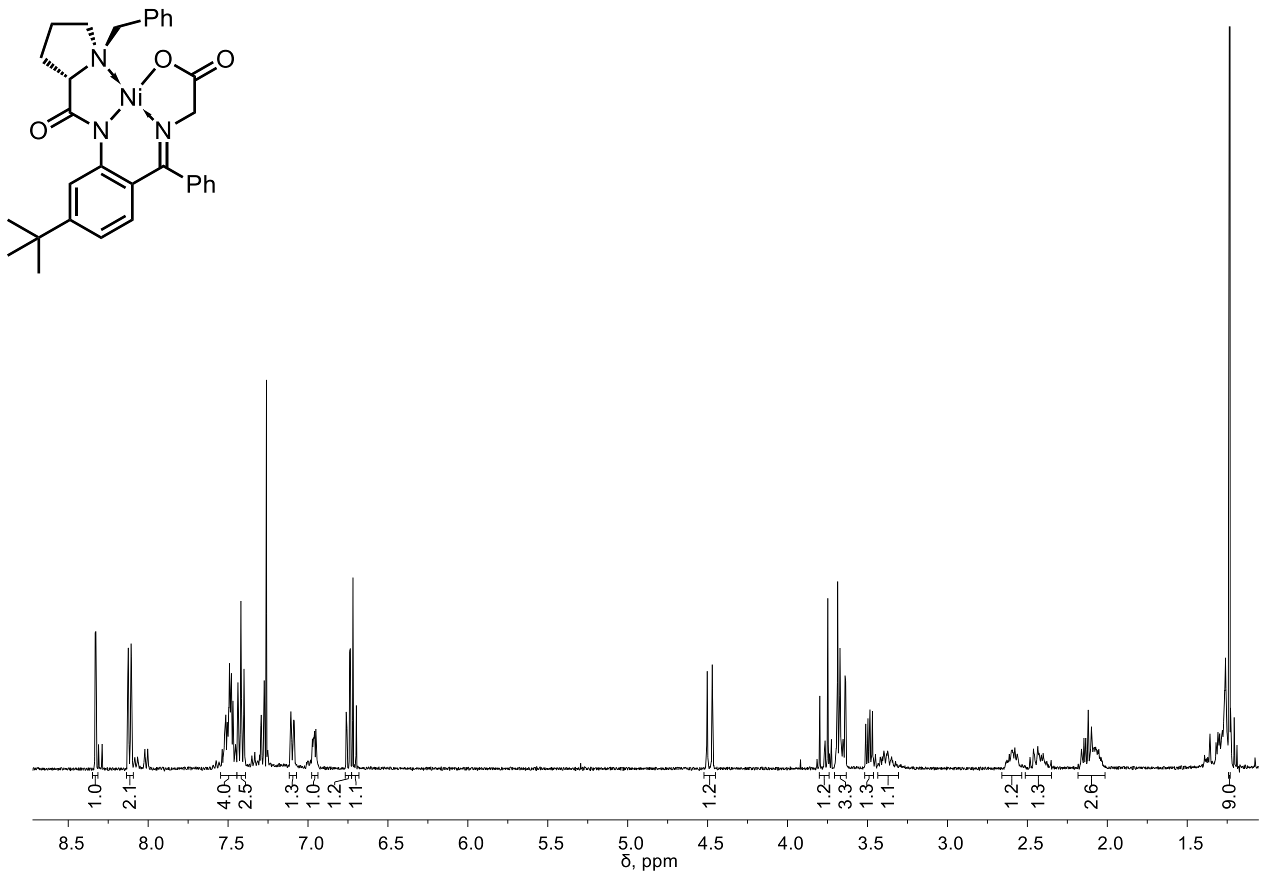

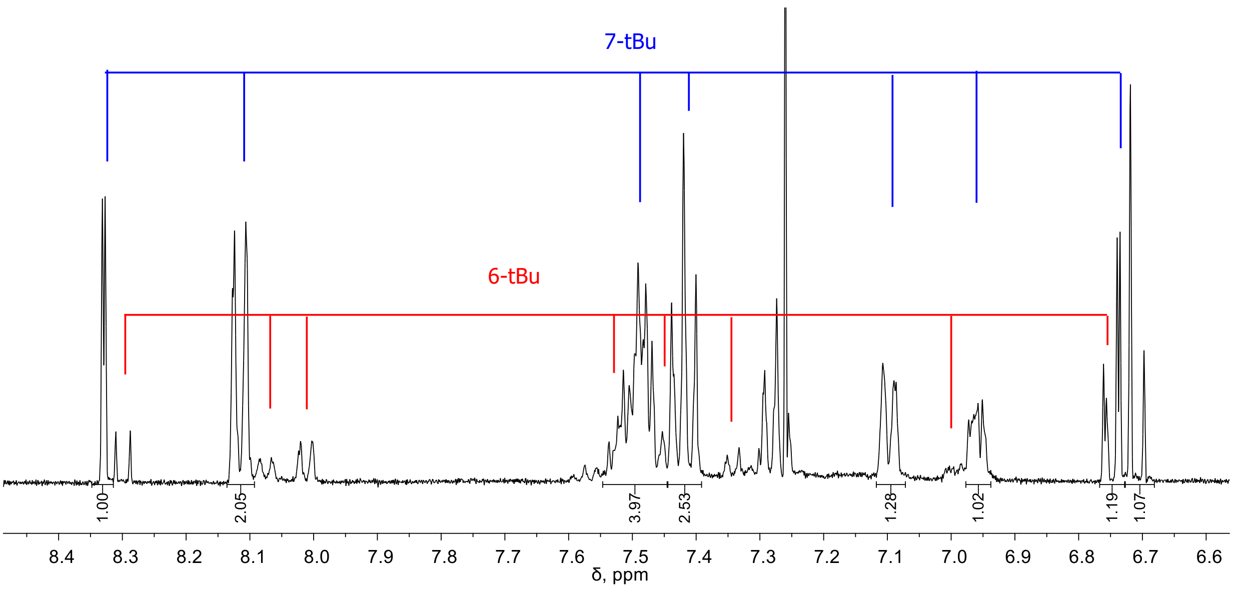

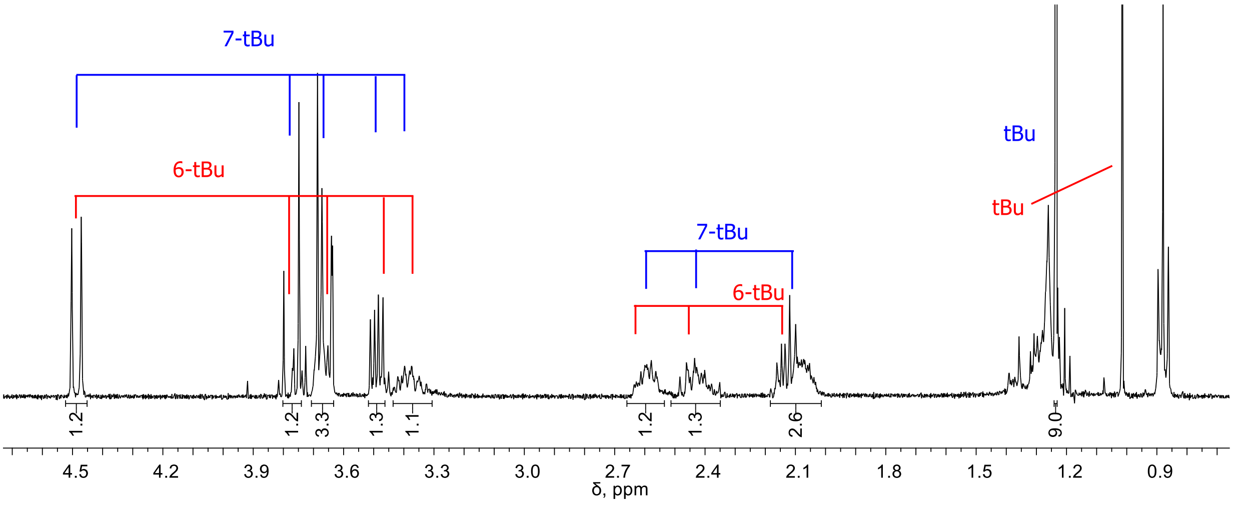

3.10. ^{13}C NMR spectrum of complex ***(GlyNi)$_{L7}$***

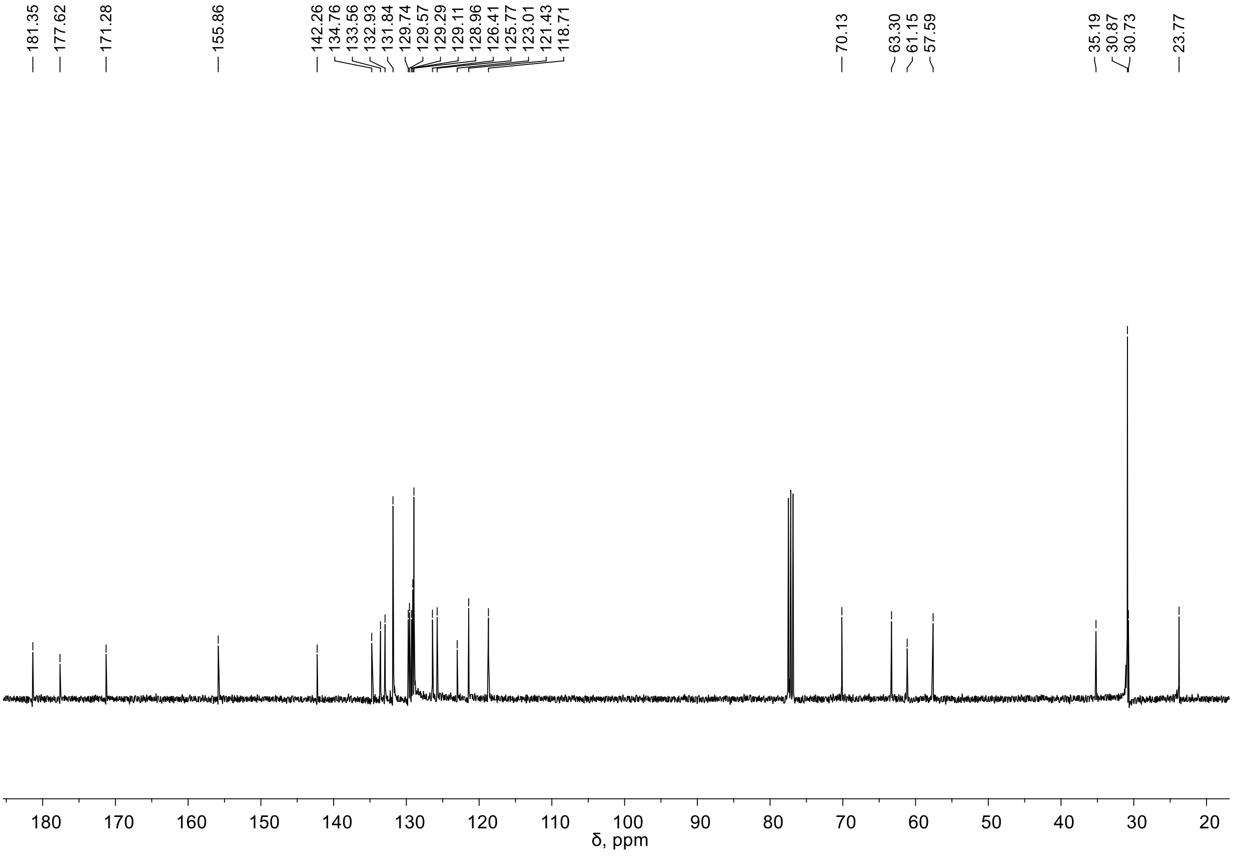

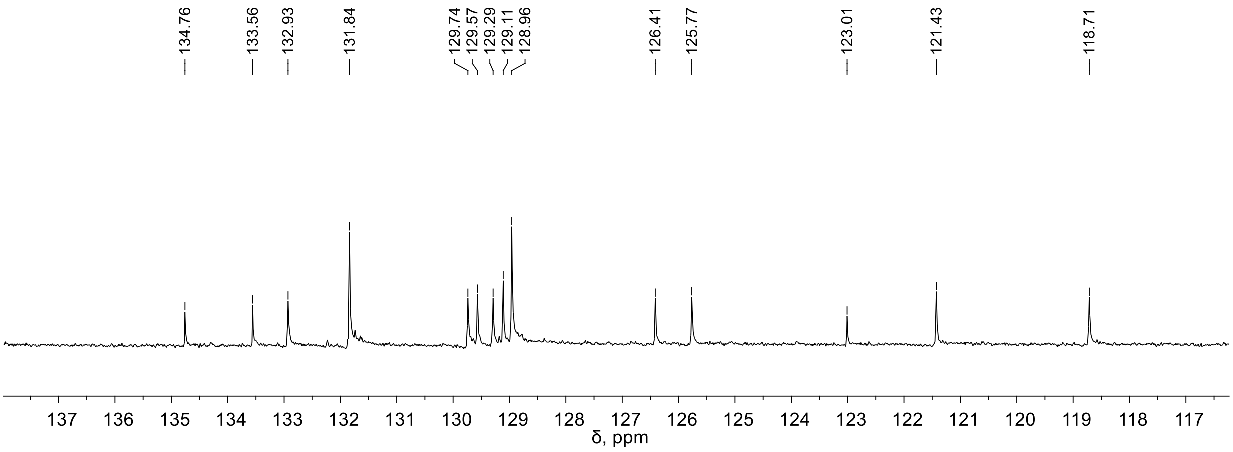

3.11. ^{1}H NMR spectrum of complex ***(2R)-(SerNi)***

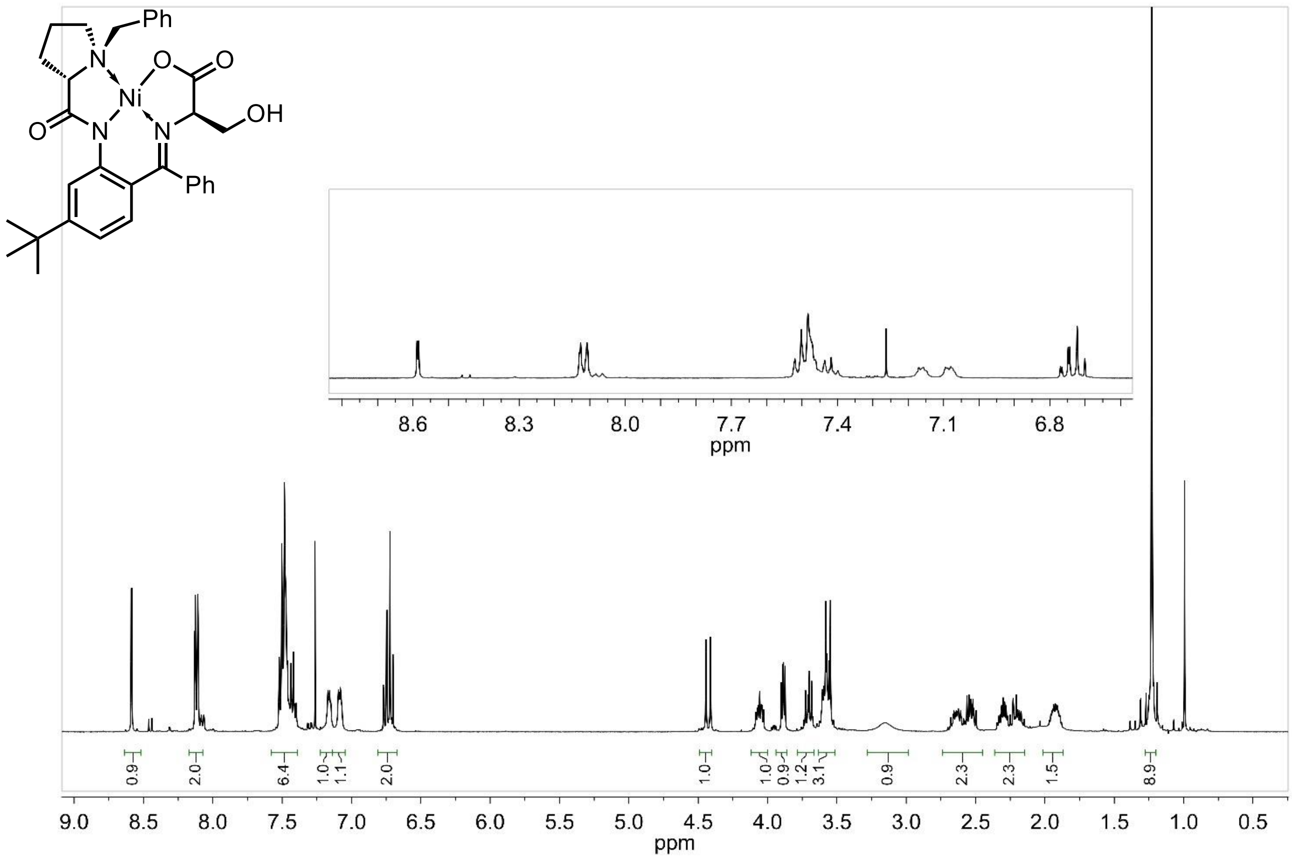

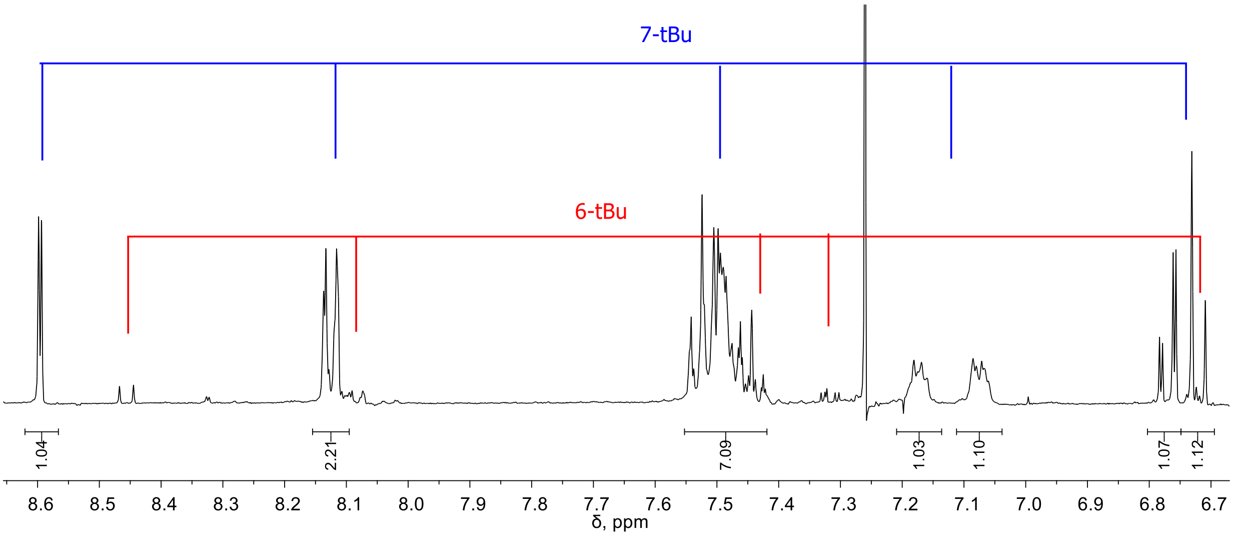
7-tBu
6-tBu
1.04
2.21
7.09
1.03
1.10
1.07
1.12
8.6 8.5 8.4 8.3 8.2 8.1 8.0 7.9 7.8 7.7 7.6 7.5 7.4 7.3 7.2 7.1 7.0 6.9 6.8 6.7
δ, ppm

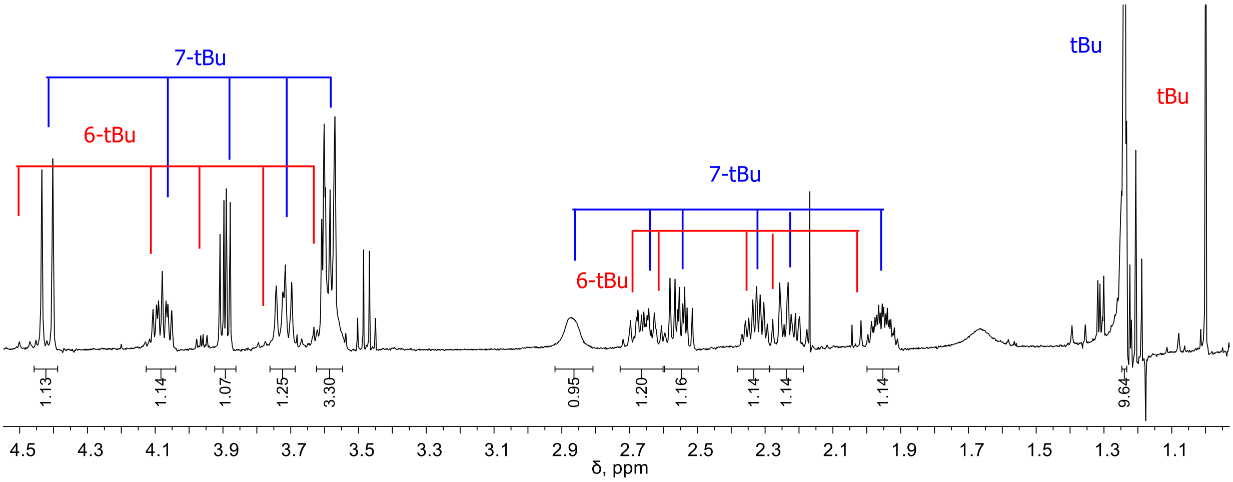
7-tBu
6-tBu
7-tBu
6-tBu
tBu
tBu
1.13
1.14
1.07
1.25
3.30
0.95
1.20
1.16
1.14
1.14
1.14
9.64
4.5 4.3 4.1 3.9 3.7 3.5 3.3 3.1 2.9 2.7 2.5 2.3 2.1 1.9 1.7 1.5 1.3 1.1
δ, ppm

3.12. ^{13}C NMR spectrum of complex **(2R)-(SerNi)**

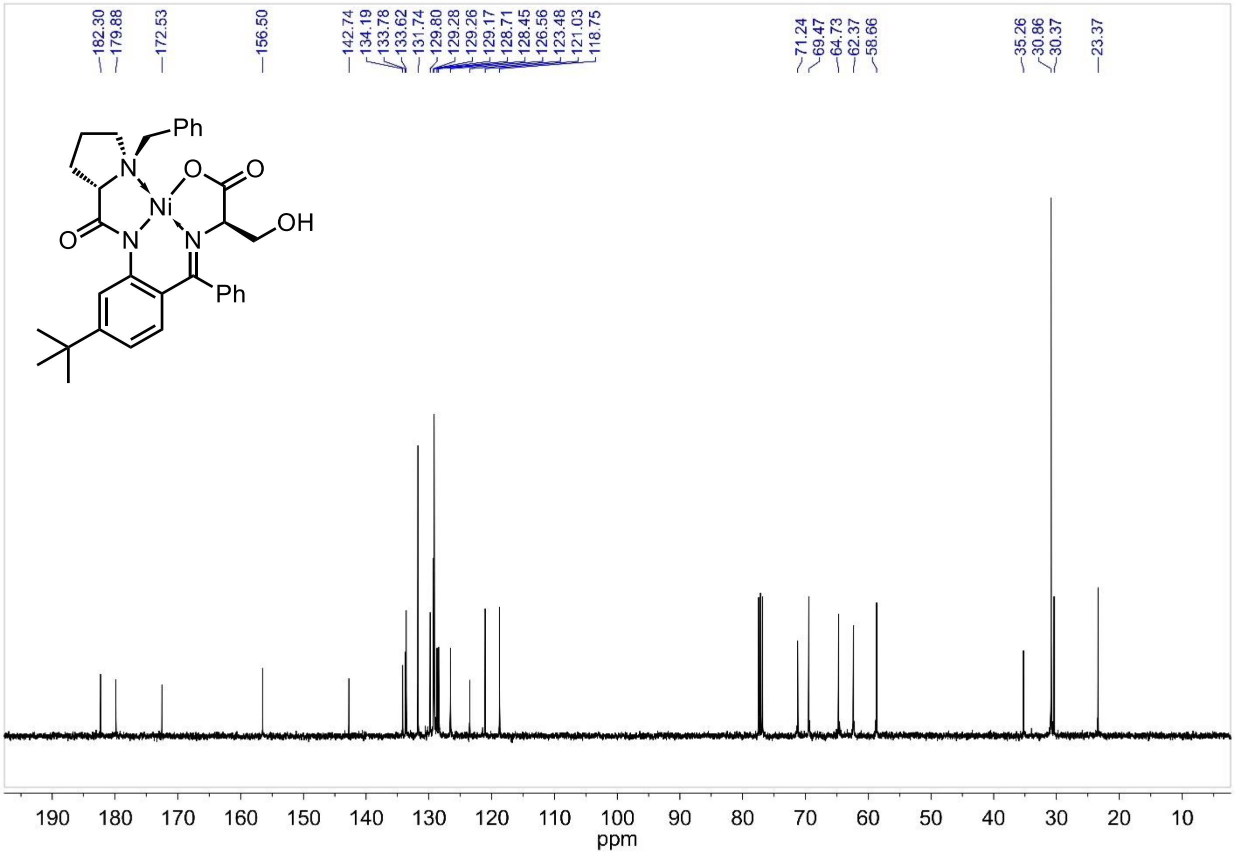

3.13. HSQC spectrum of complex **(2R)-(SerNi)**

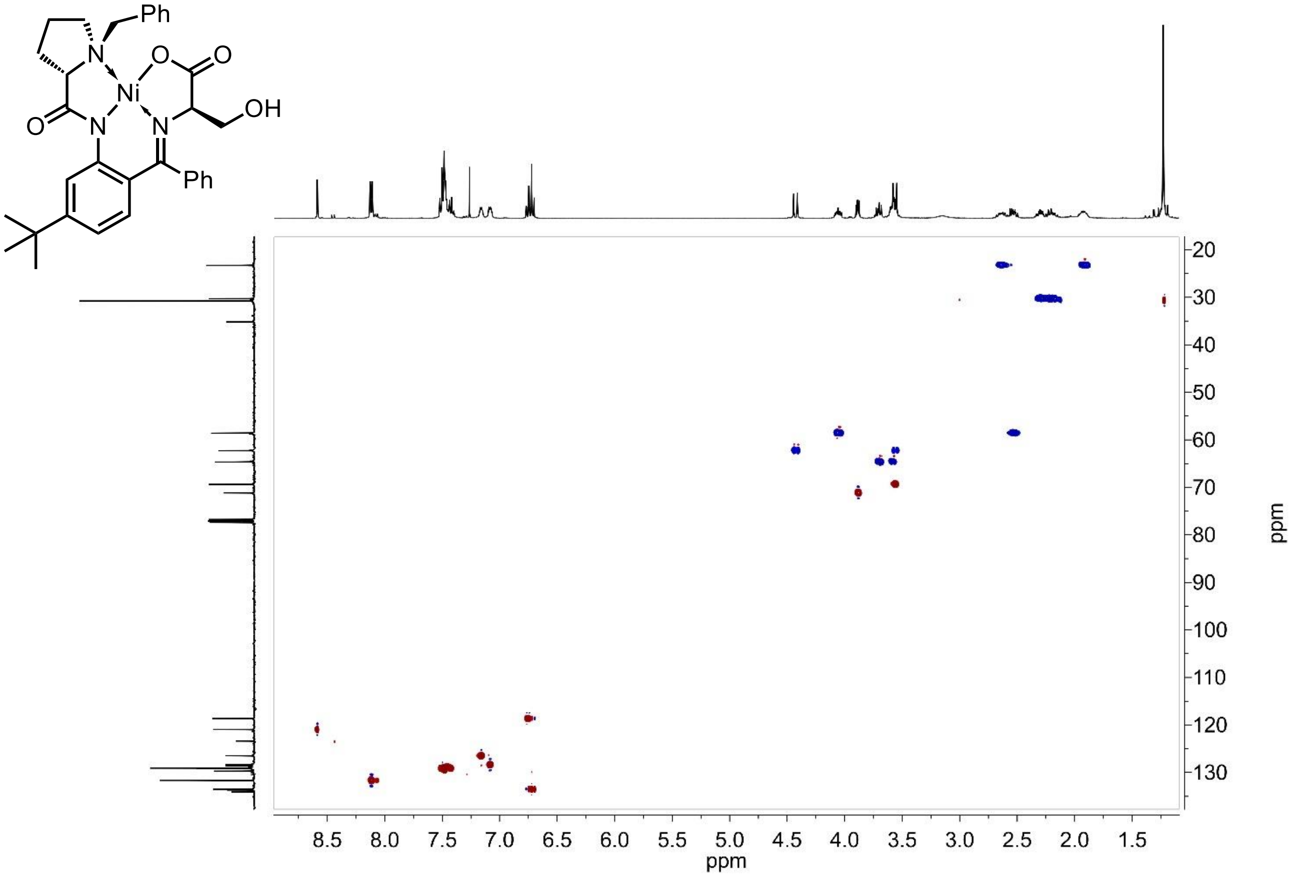

3.14. ^1H NMR spectrum of complex **(2S)-(SerNi)**

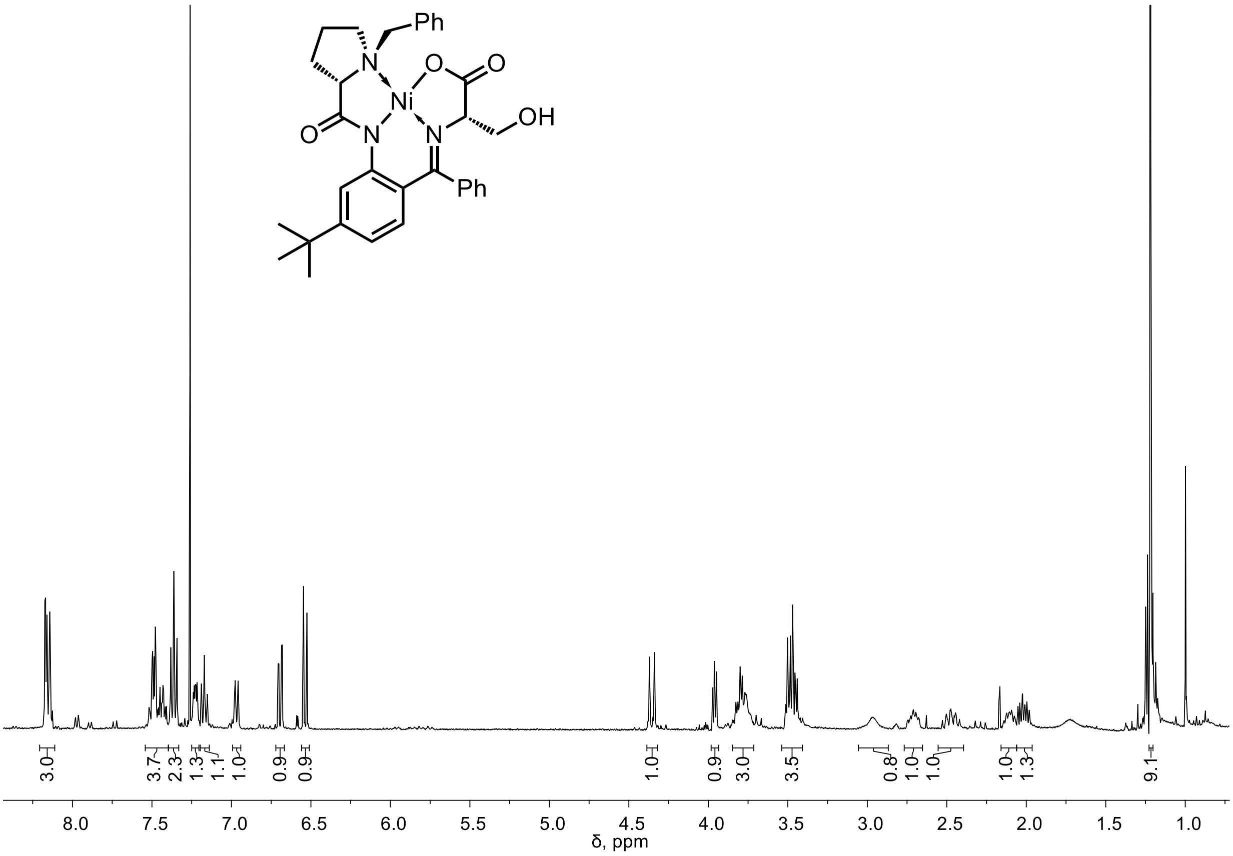

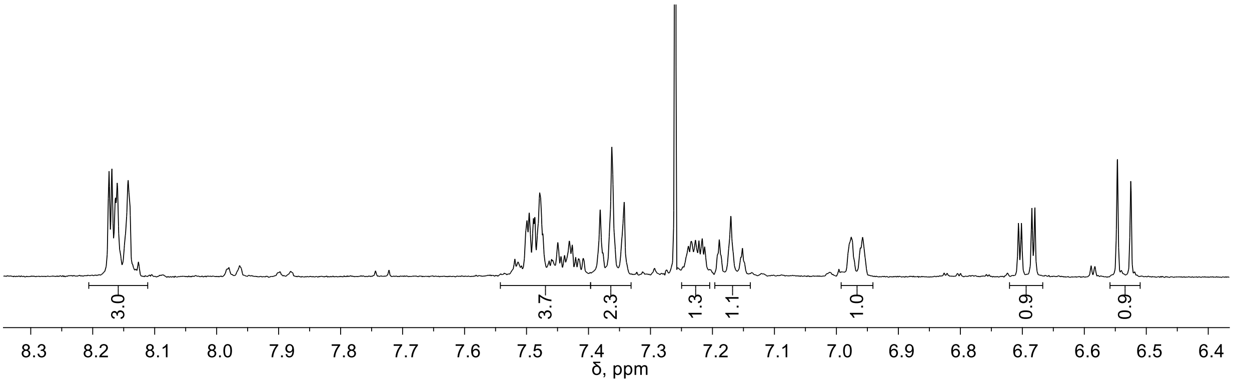

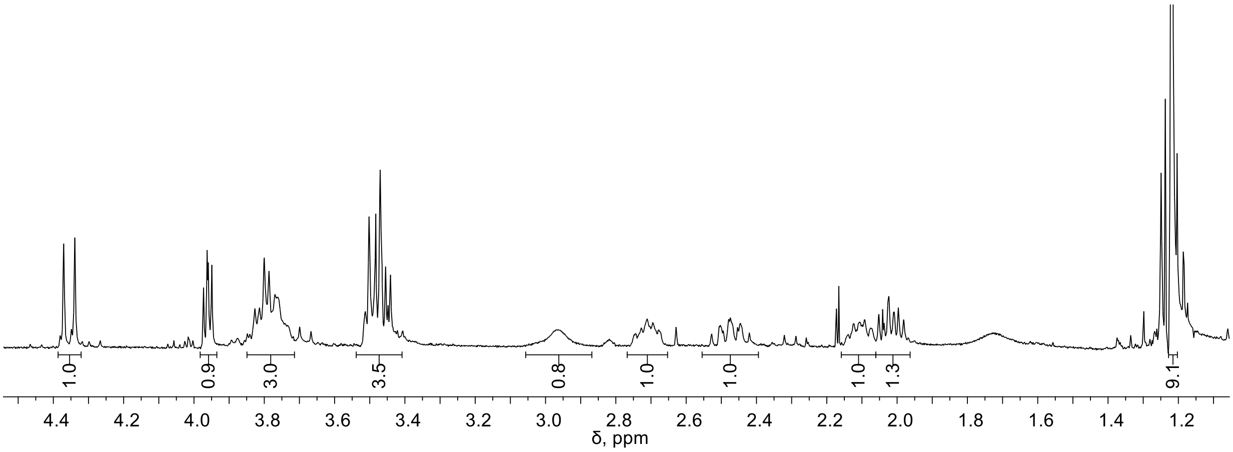

3.15. ^{1}H NMR spectrum of complex **(ΔAlaNi)$_{L7}$**

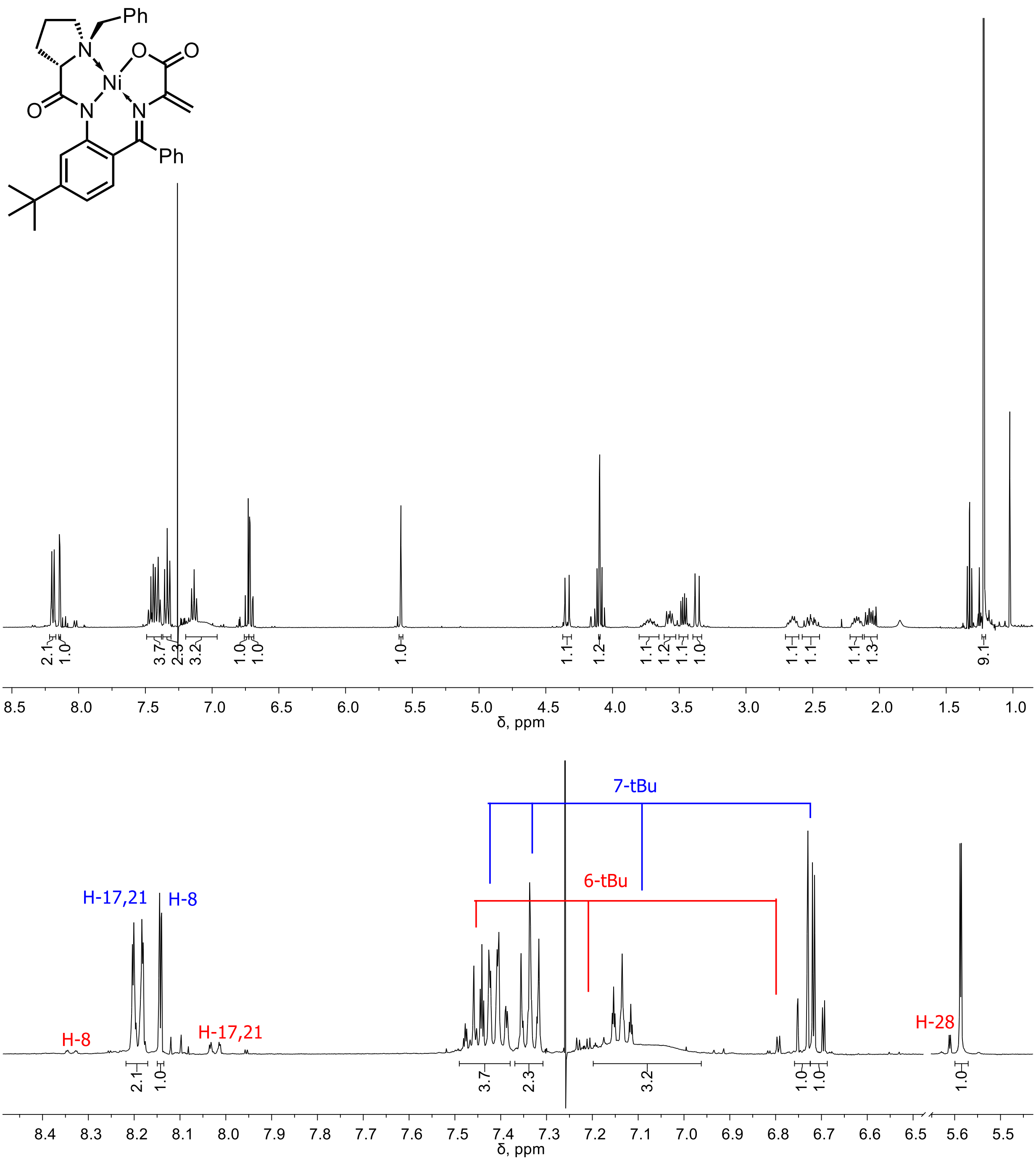

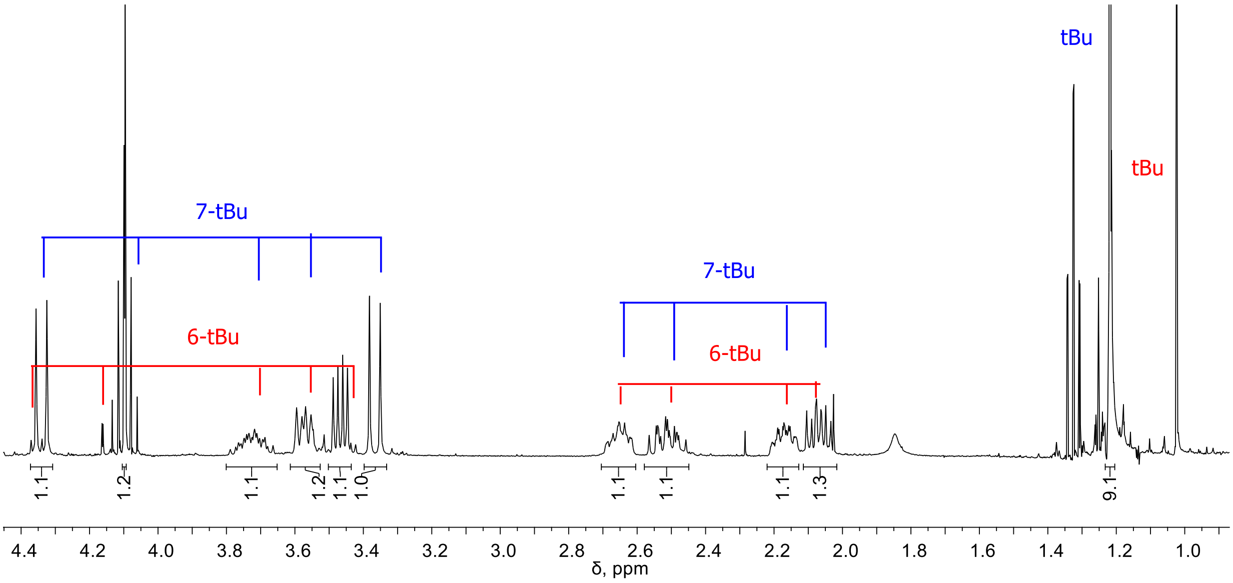
tBu
tBu
7-tBu
6-tBu
7-tBu
6-tBu
1.1
1.2
1.1
1.2
1.1
1.0
1.1
1.1
1.1
1.3
9.1
4.4 4.2 4.0 3.8 3.6 3.4 3.2 3.0 2.8 2.6 2.4 2.2 2.0 1.8 1.6 1.4 1.2 1.0
δ, ppm

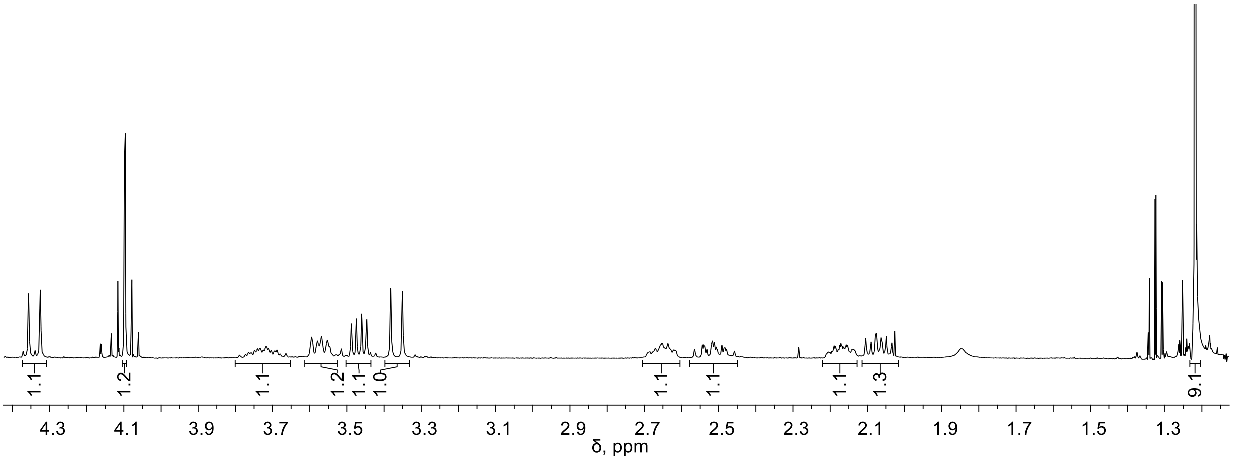
1.1
1.2
1.1
1.2
1.1
1.0
1.1
1.1
1.1
1.3
9.1
4.3 4.1 3.9 3.7 3.5 3.3 3.1 2.9 2.7 2.5 2.3 2.1 1.9 1.7 1.5 1.3
δ, ppm

3.16. ^{13}C NMR spectrum of complex **(ΔAlaNi)$_{L7}$**

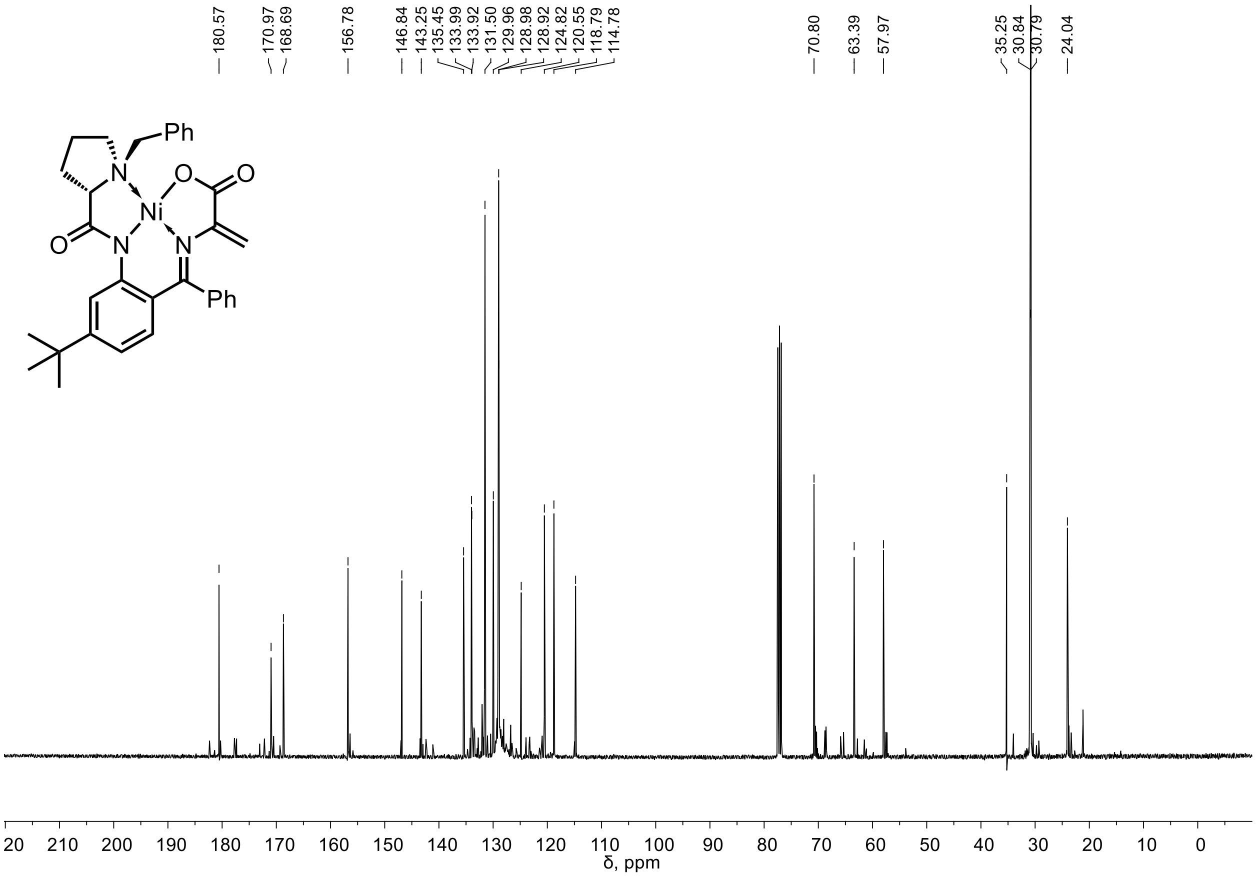

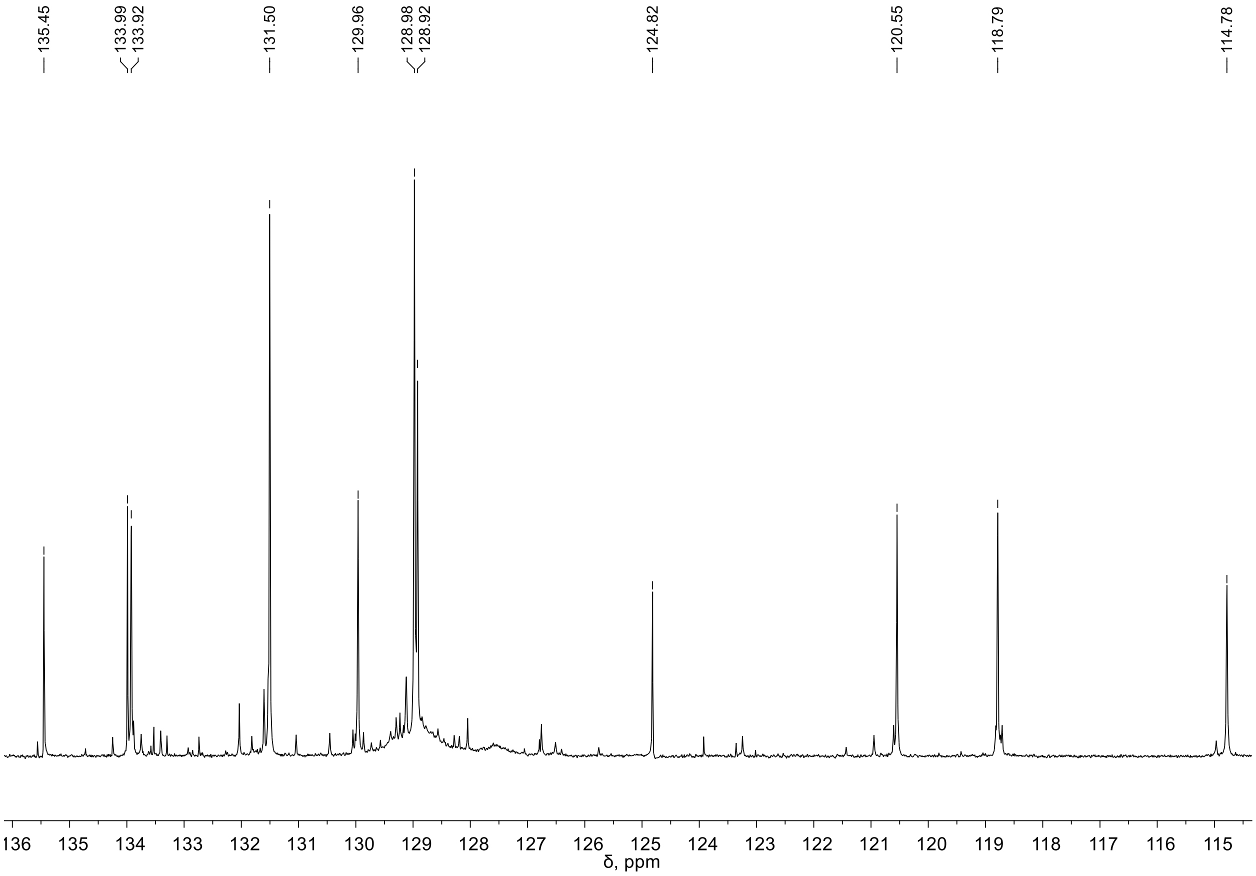

3.17. HSQC spectrum of complex ***(ΔAlaNi)$_{L7}$***

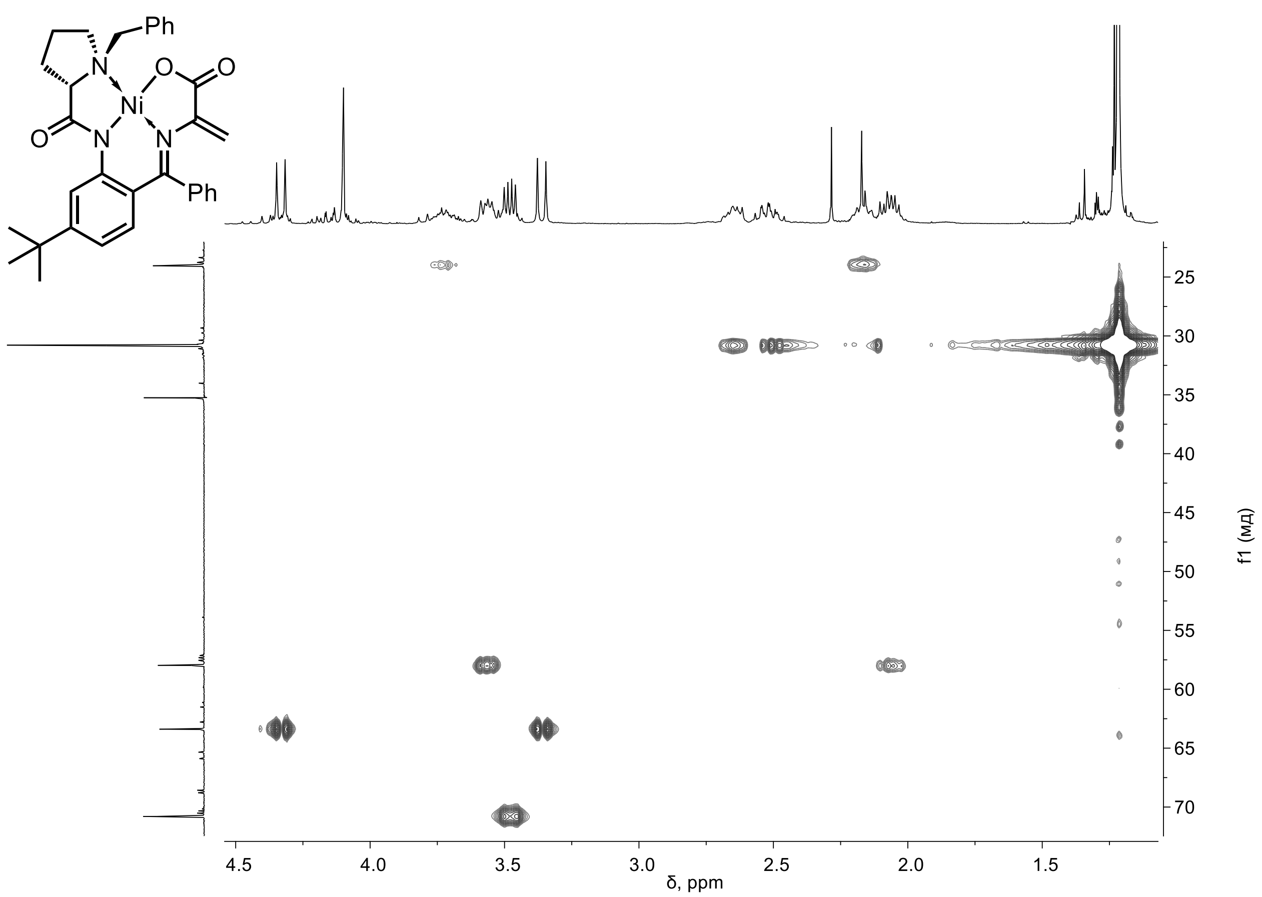

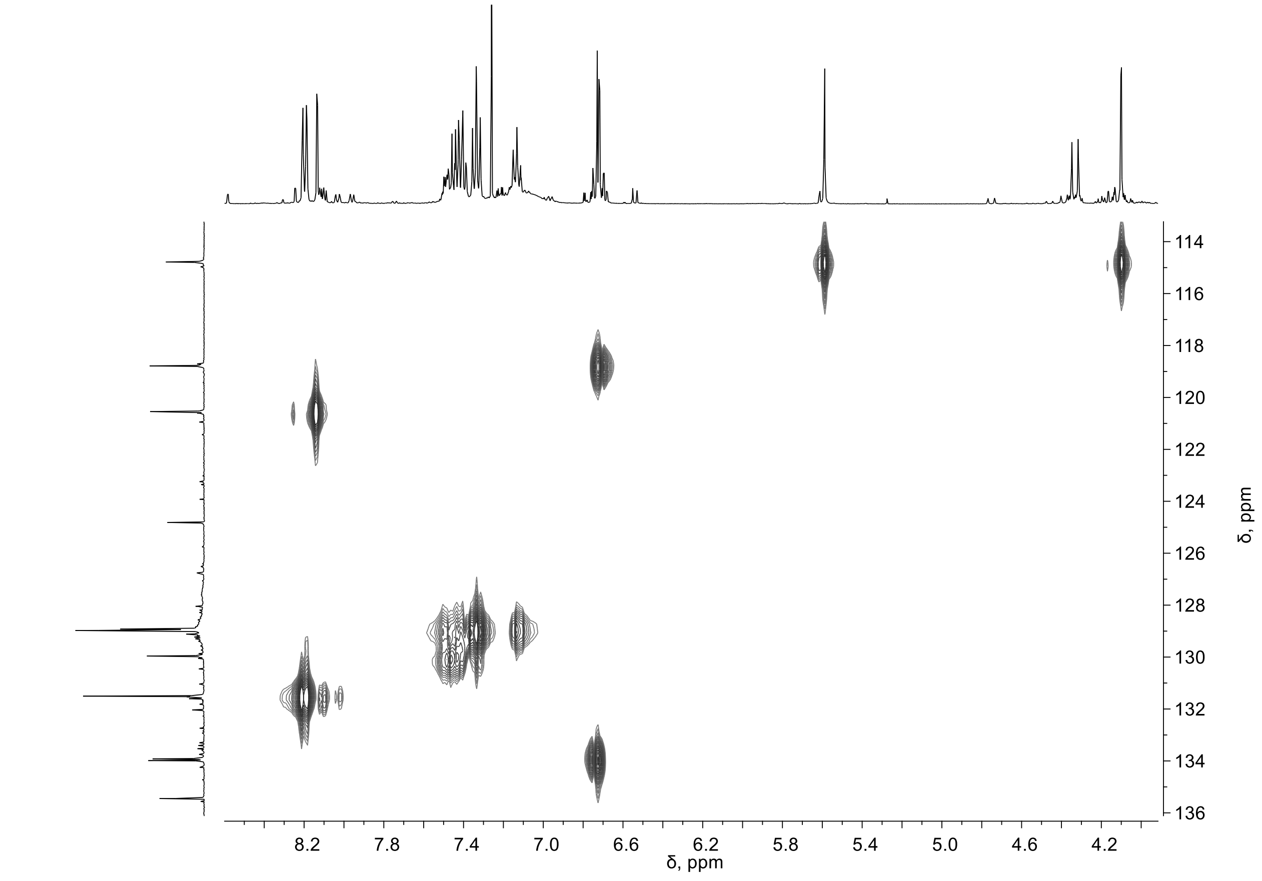

*3.18. HMBC spectrum of complex **(ΔAlaNi)$_{L7}$***

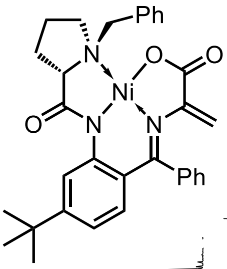

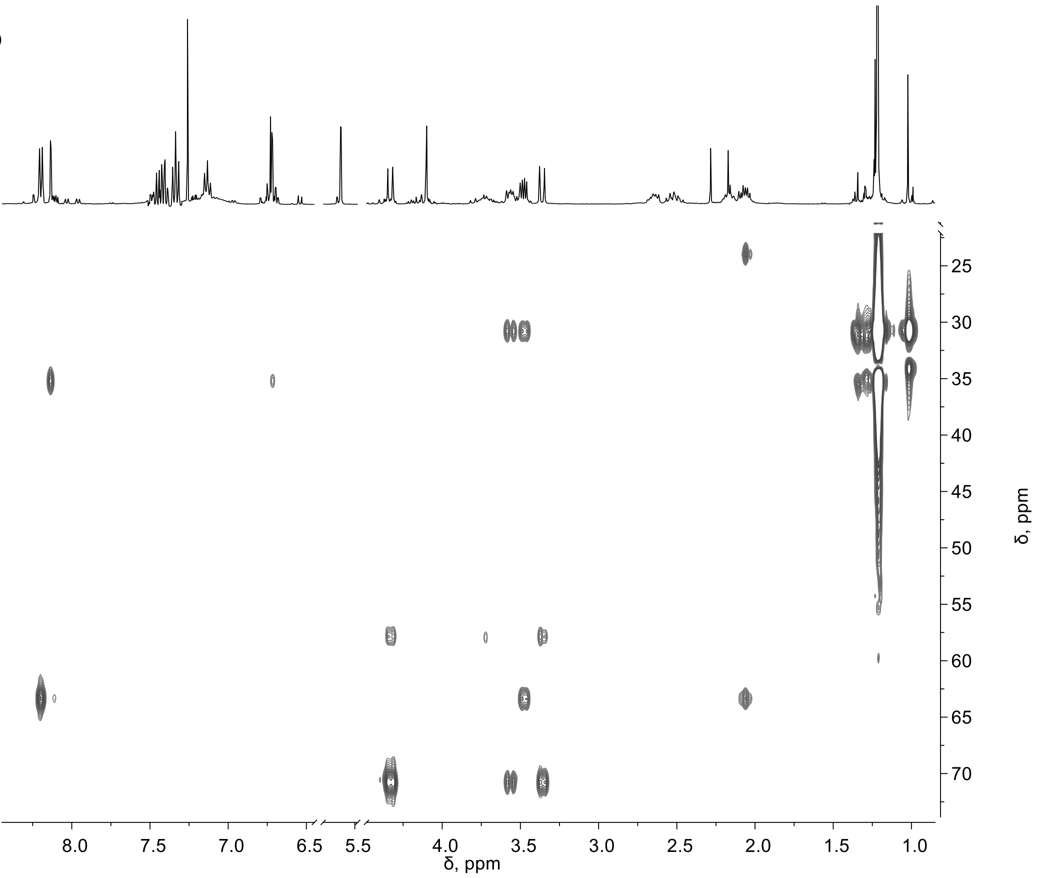

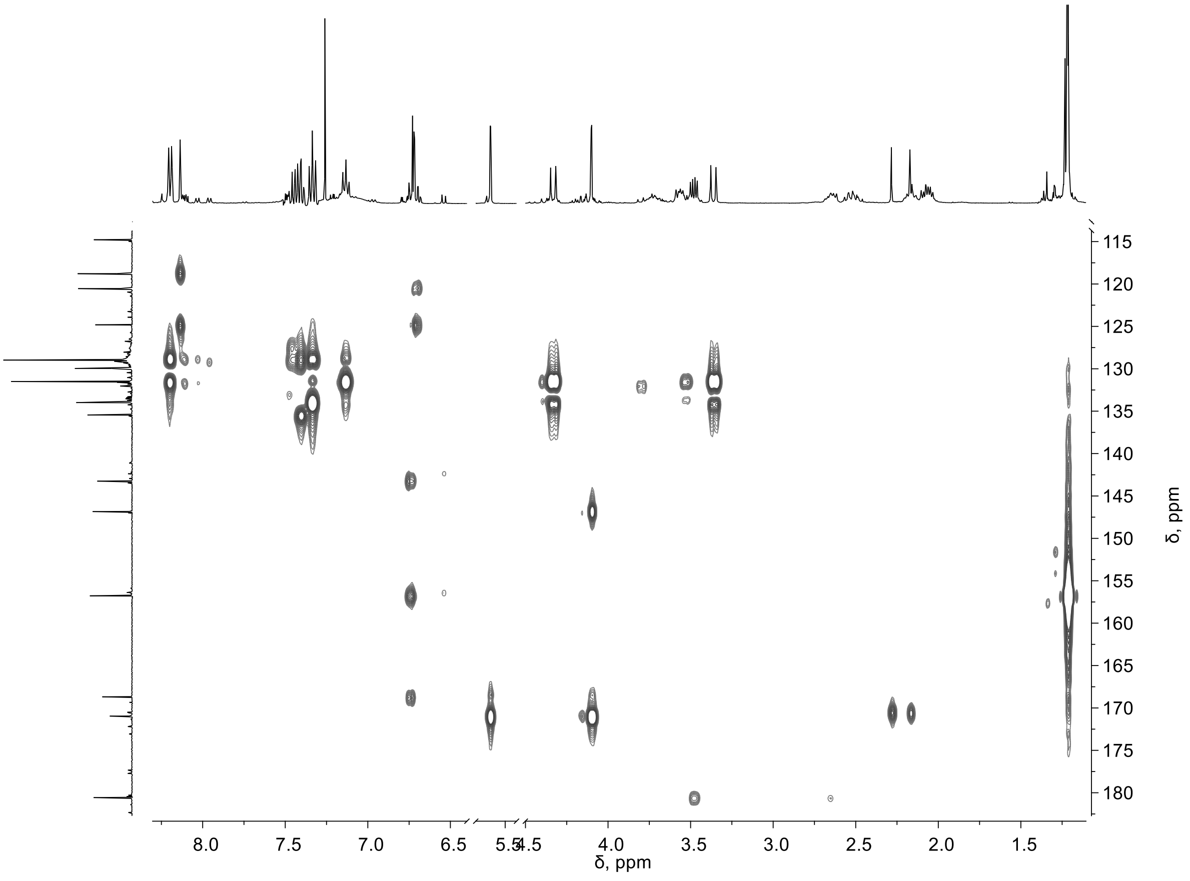

3.19. *^{1}H NMR spectrum of complex* ***(^{Bn}CysNi)$_{L7}$***

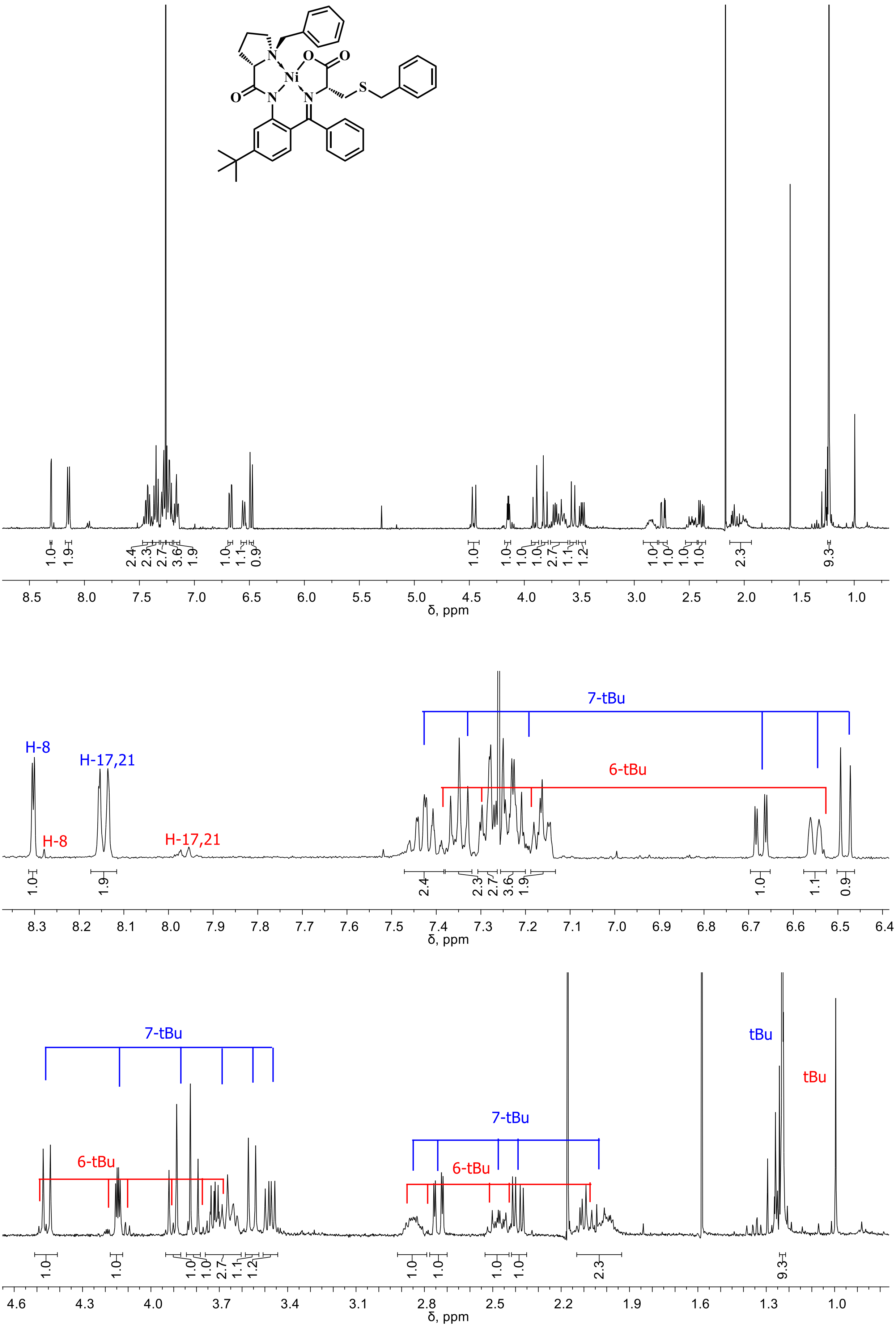

3.20. ^{13}C NMR spectrum of complex **(^{Bn}CysNi)$_{L7}$**

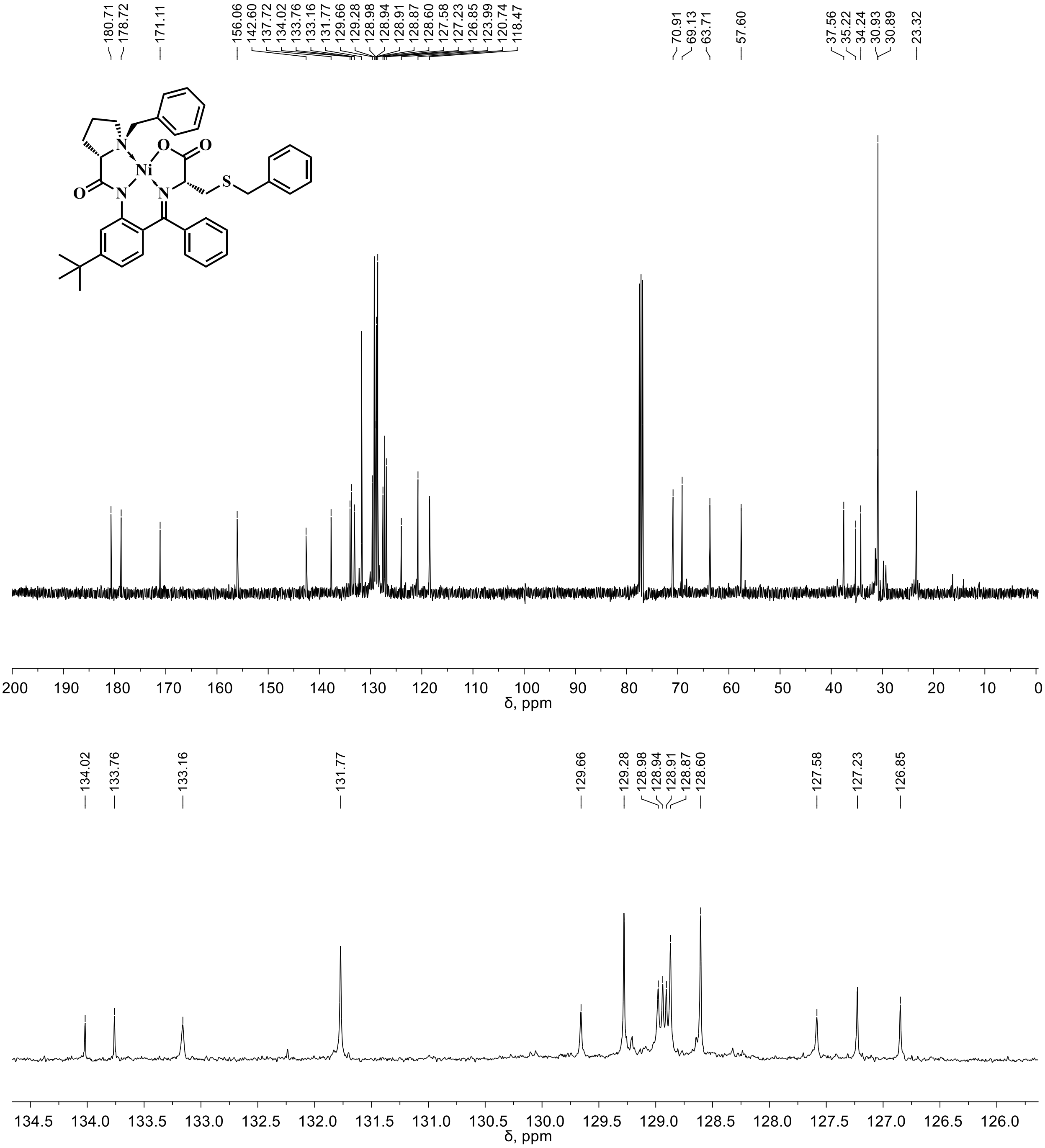

3.21. HSQC spectrum of complex *(**^{Bn}CysNi)$_{L7}$***

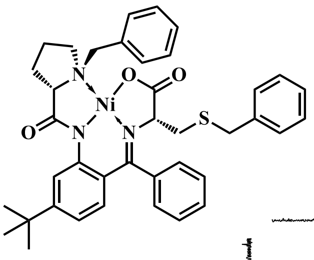

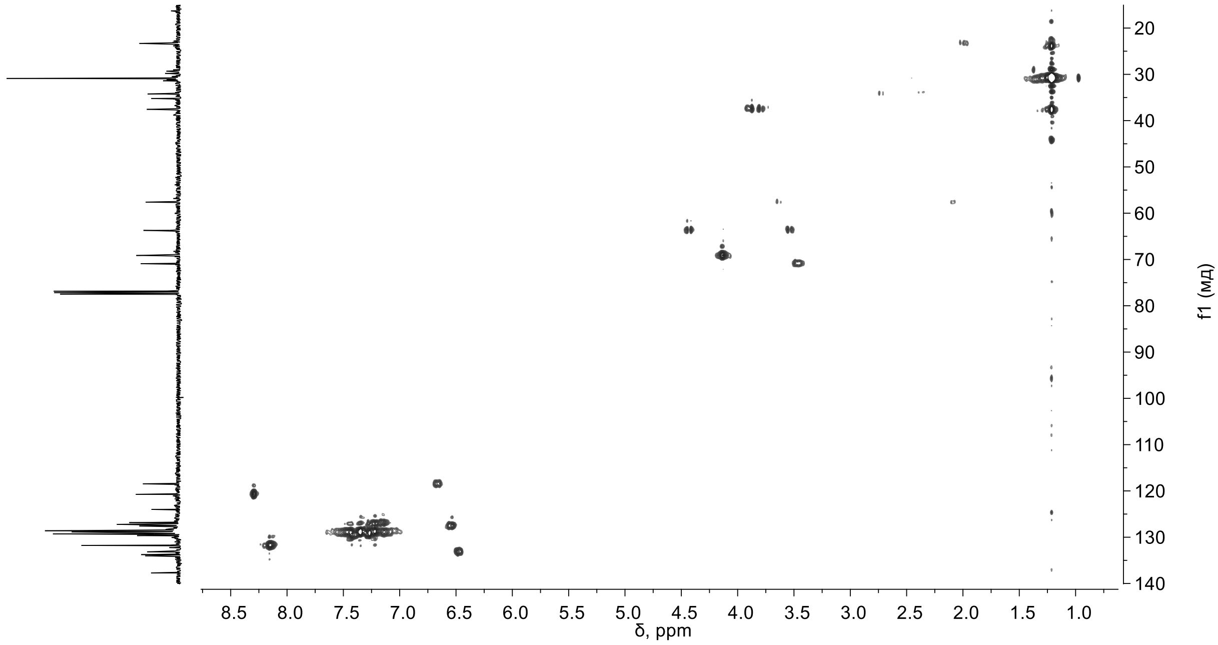

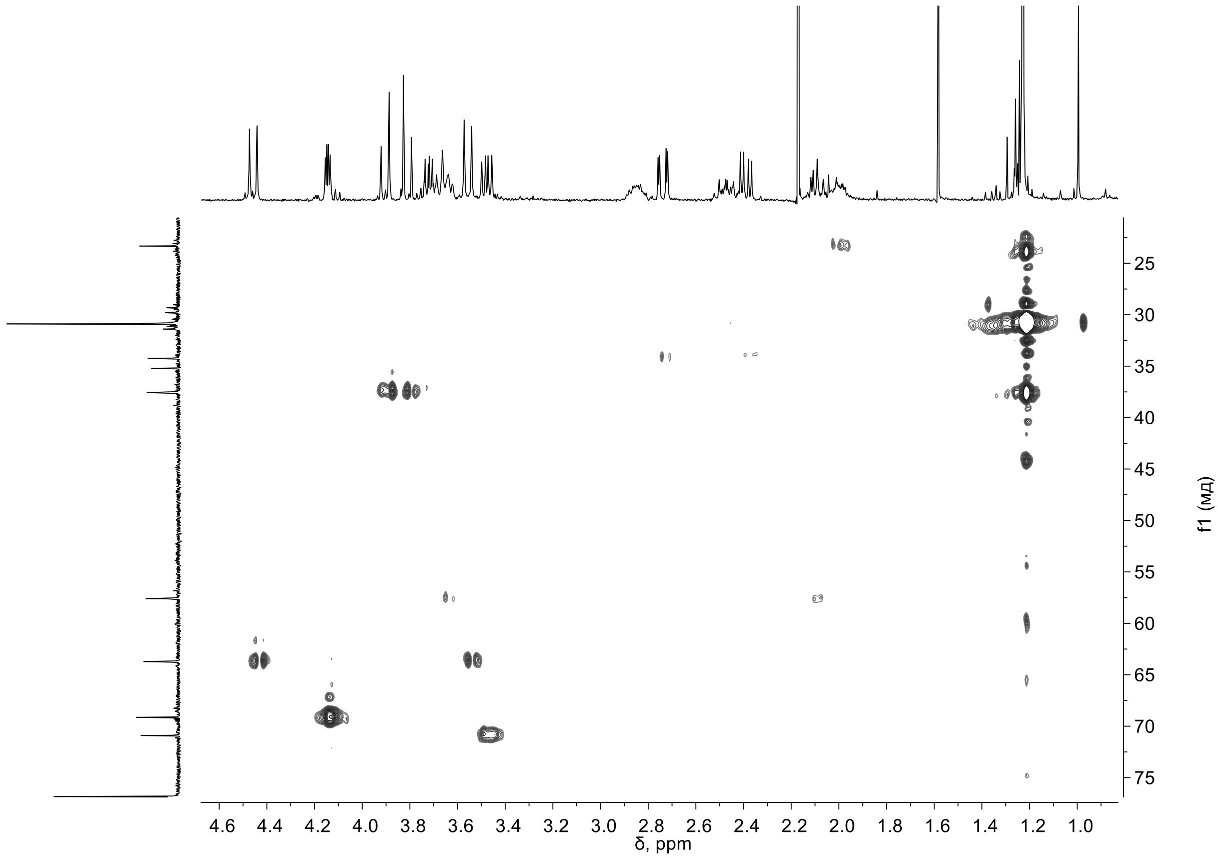

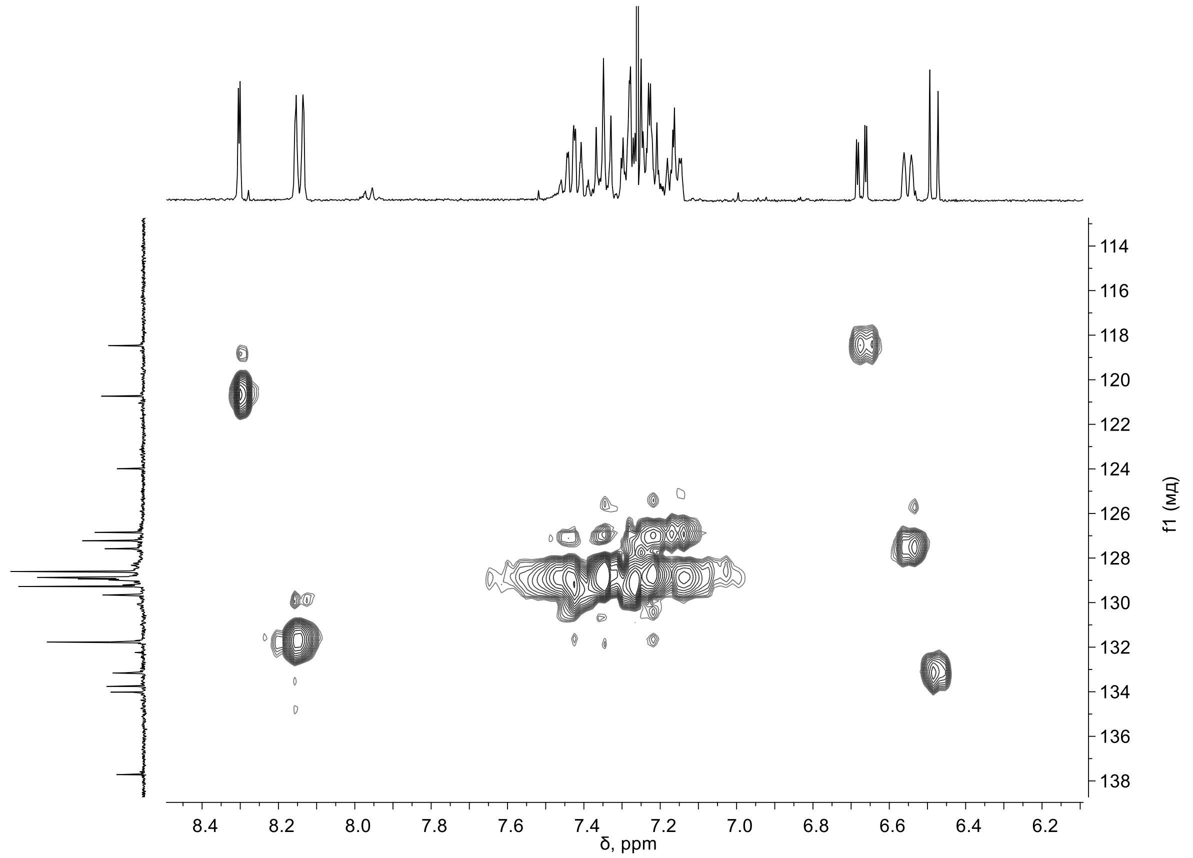

3.22. HMBC spectrum of complex $\boldsymbol{(^{Bn}CysNi)_{L7}}$

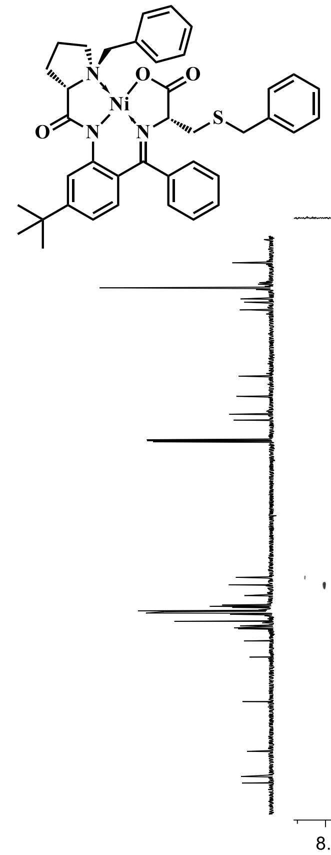

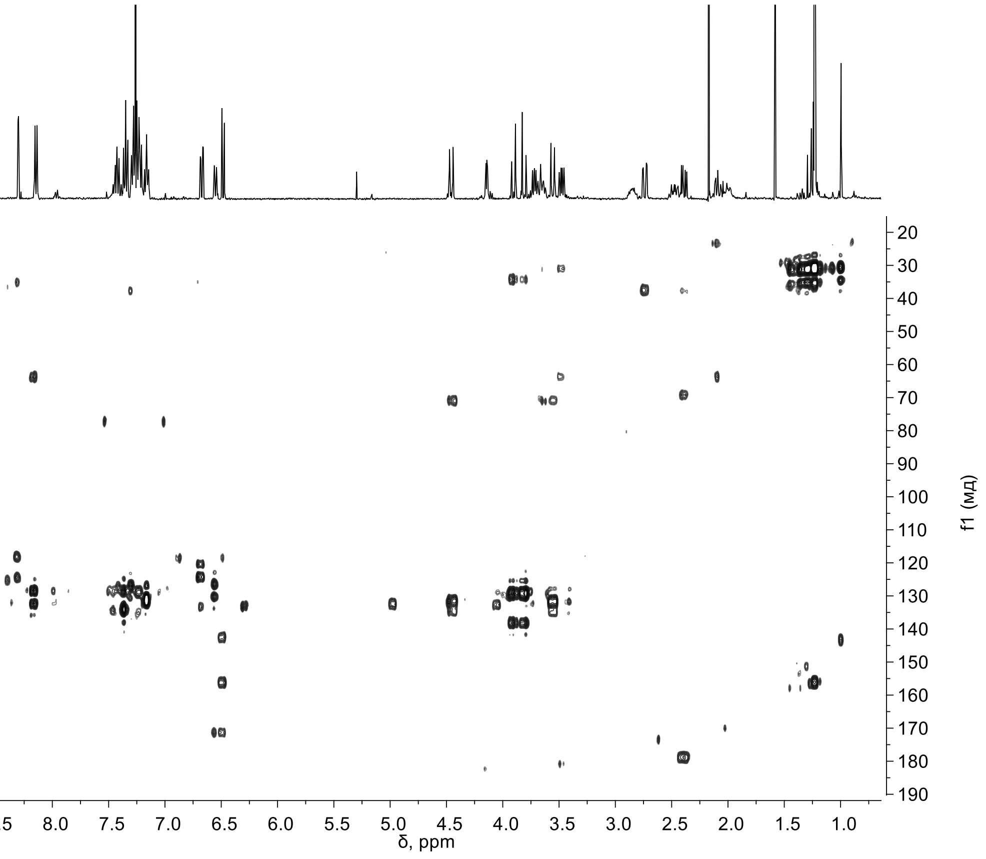

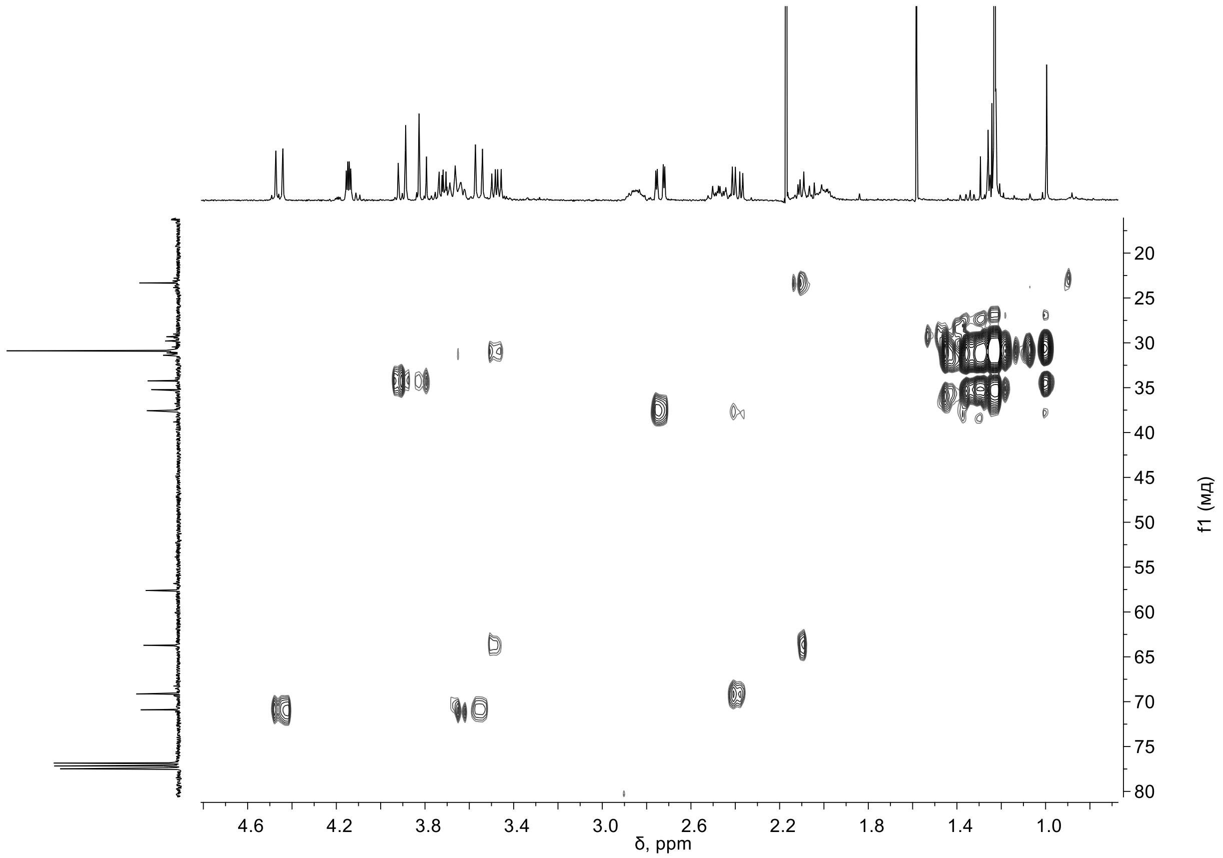
f1 (мд)
δ, ppm

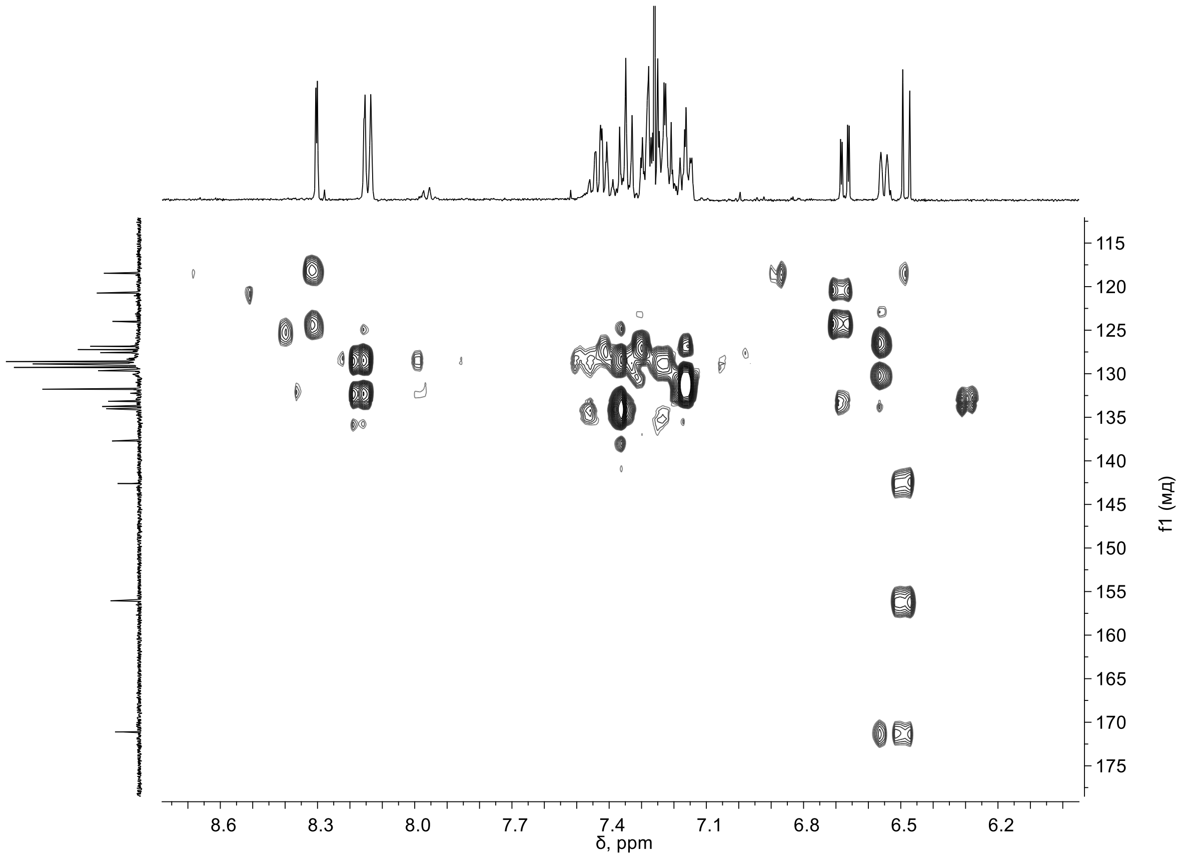
f1 (мд)
δ, ppm

3.23. ^{1}H NMR spectrum of complex **(^{Ph}CysNi)$_{L7}$**

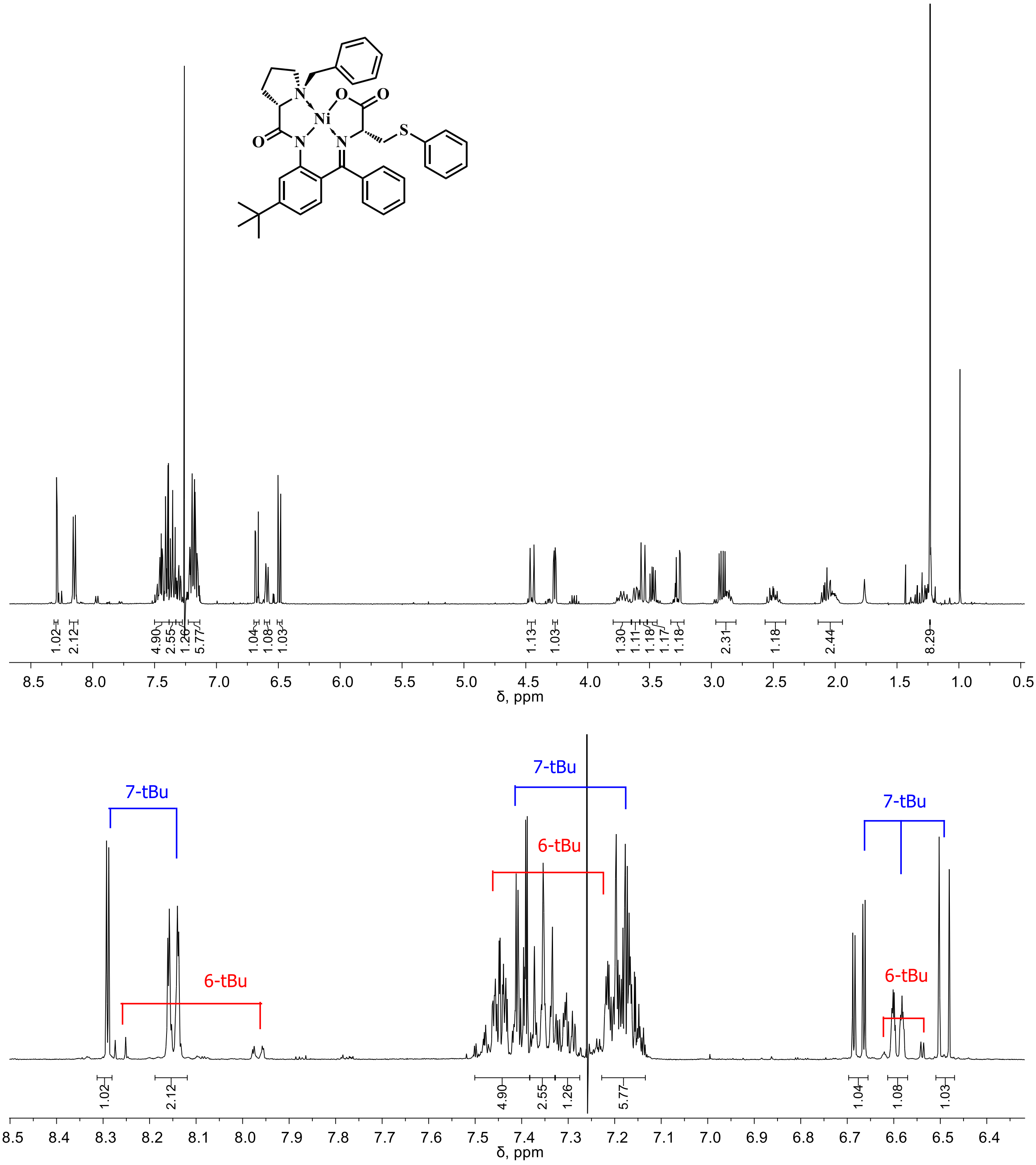

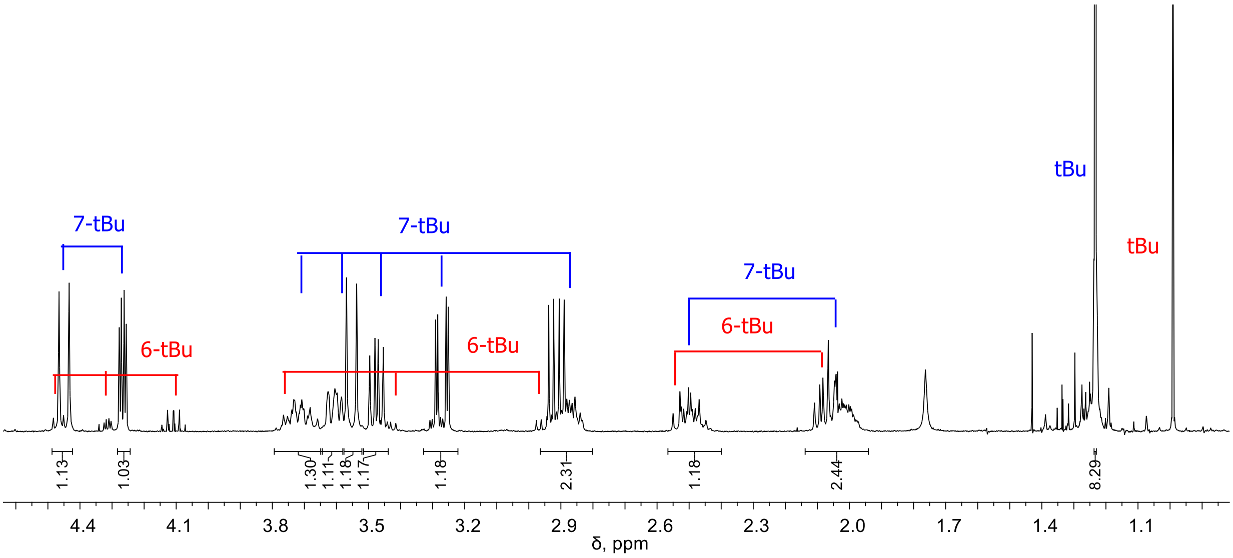

*3.24. ^{13}C NMR spectrum of complex **(^{Ph}CysNi)$_{L7}$***

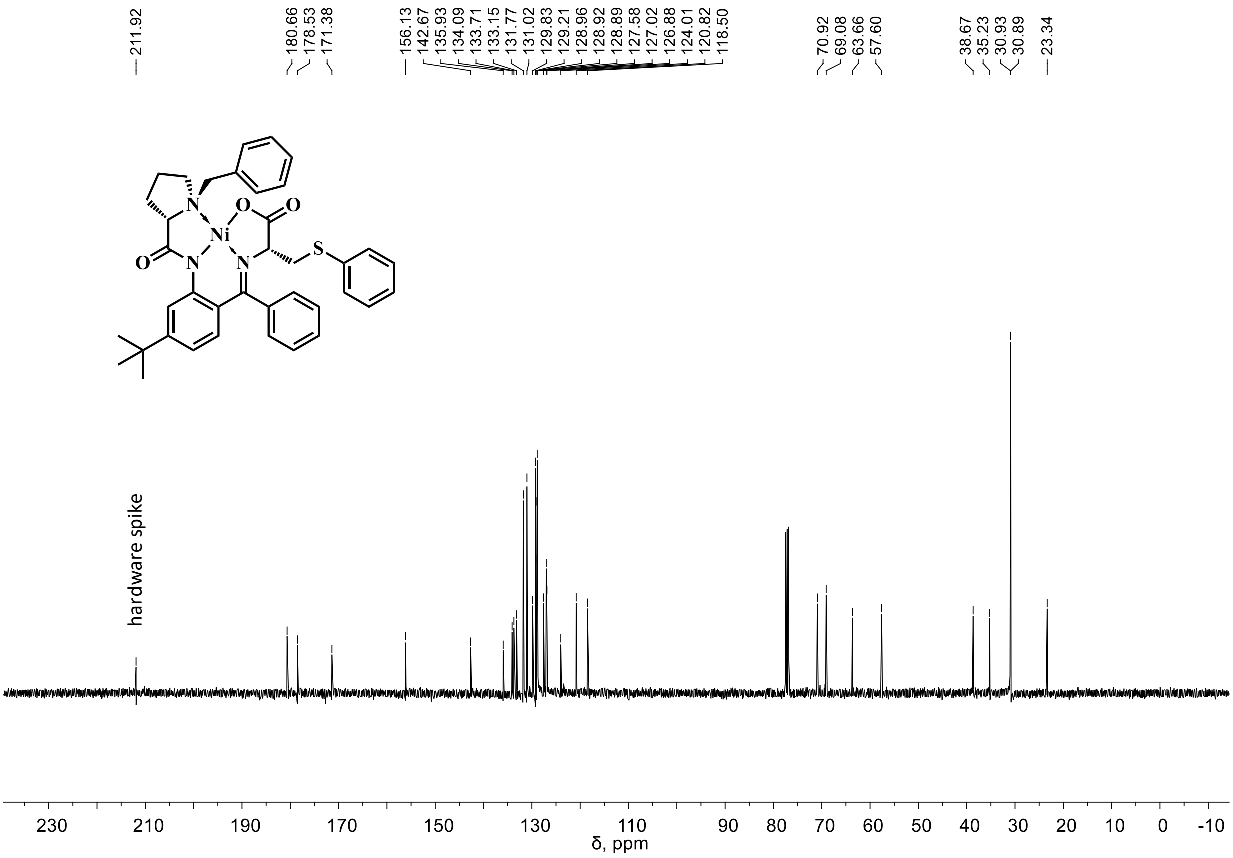

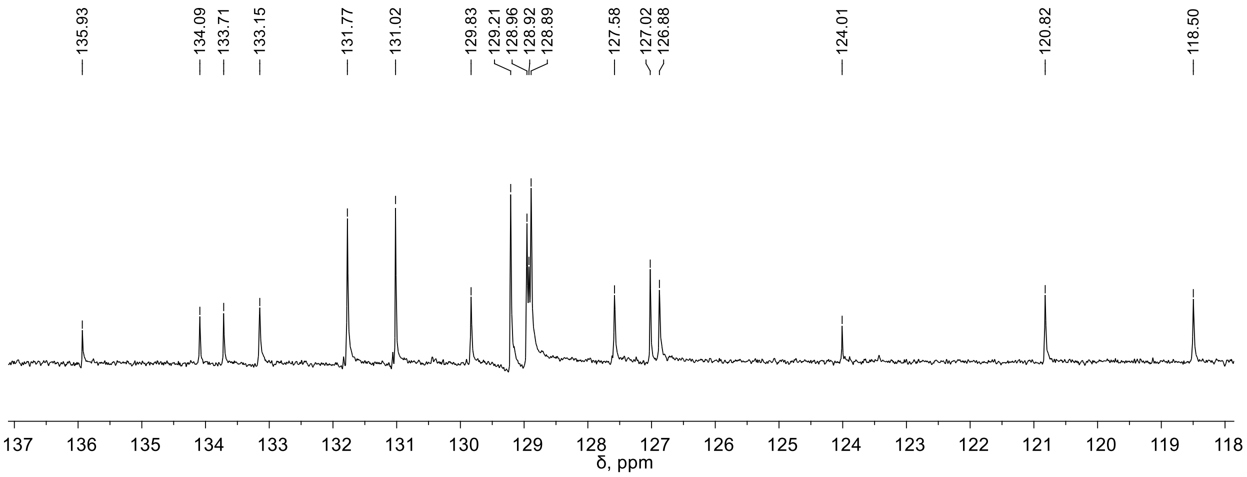

3.25. ^{1}H NMR spectrum of complex **(^{pMe}CysNi)$_{L7}$**

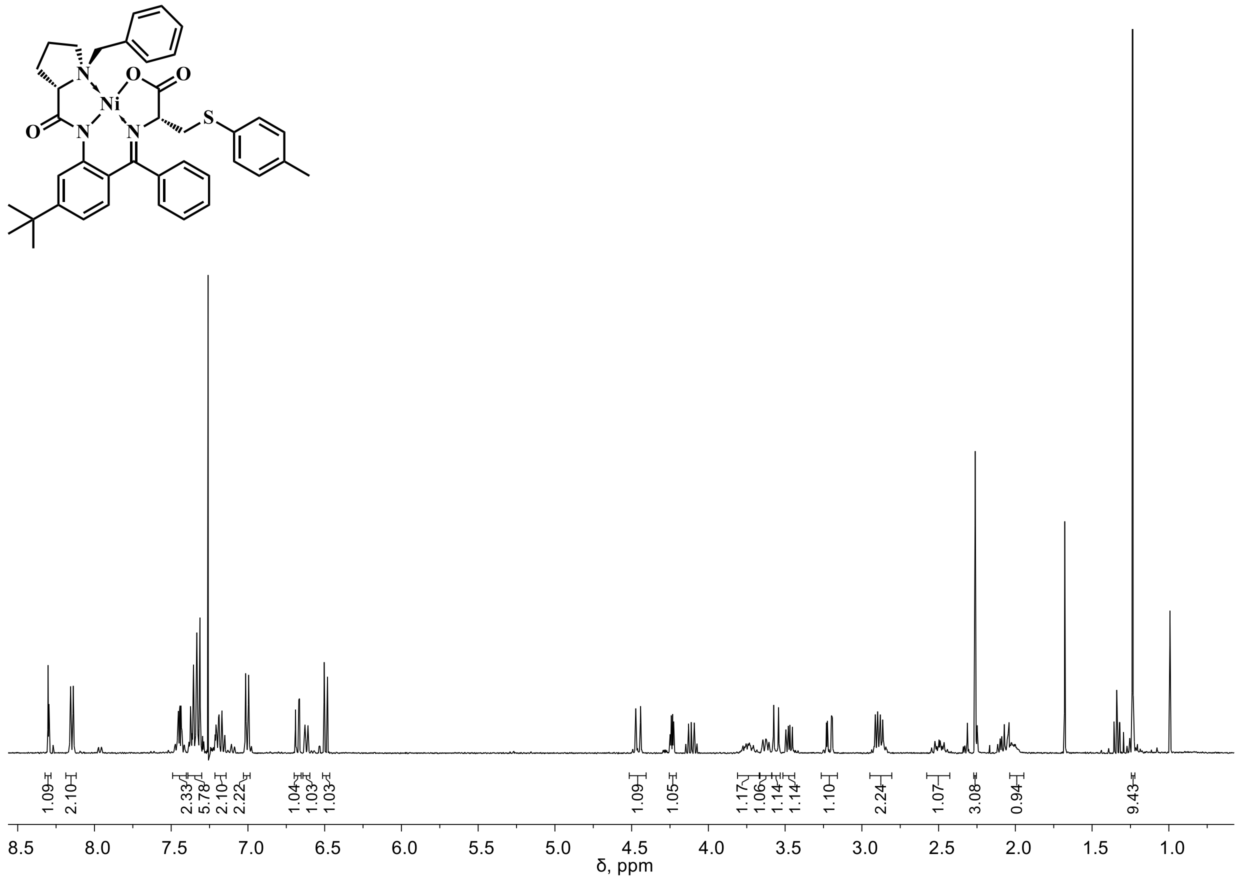

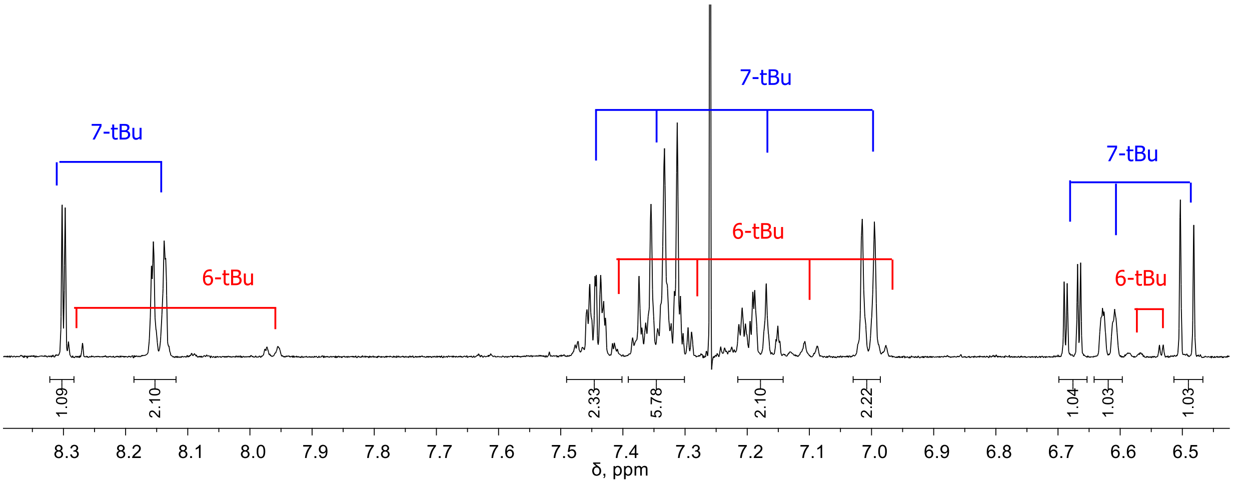
7-tBu
6-tBu
7-tBu
6-tBu
7-tBu
6-tBu
1.09
2.10
2.33
5.78
2.10
2.22
1.04
1.03
1.03
8.3
8.2
8.1
8.0
7.9
7.8
7.7
7.6
7.5
7.4
7.3
7.2
7.1
7.0
6.9
6.8
6.7
6.6
6.5
δ, ppm

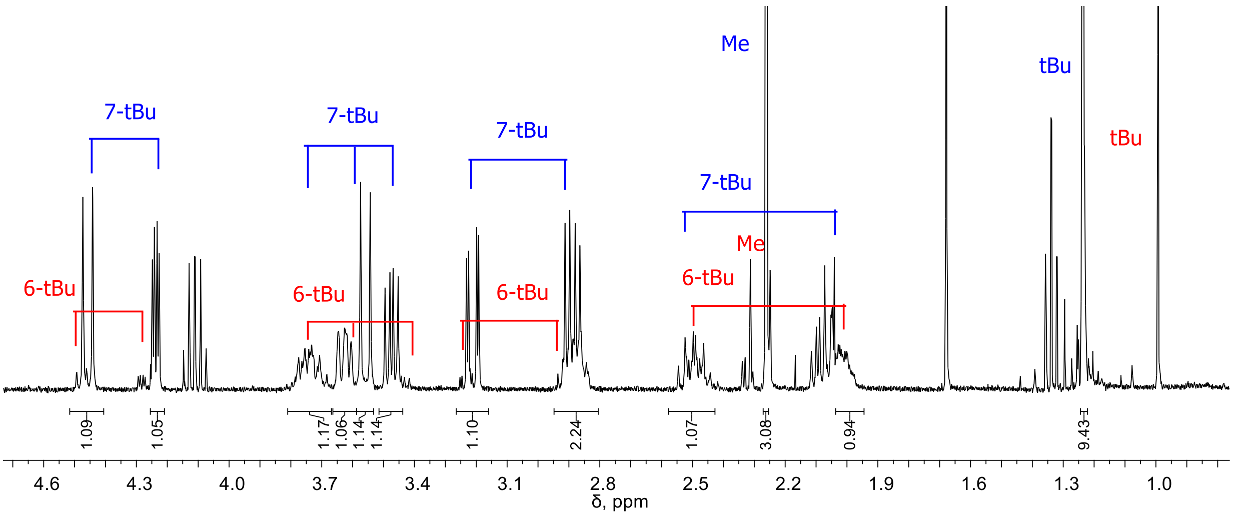
7-tBu
6-tBu
7-tBu
6-tBu
7-tBu
6-tBu
Me
7-tBu
Me
6-tBu
tBu
tBu
1.09
1.05
1.17
1.06
1.14
1.14
1.10
2.24
1.07
3.08
0.94
9.43
4.6
4.3
4.0
3.7
3.4
3.1
2.8
2.5
2.2
1.9
1.6
1.3
1.0
δ, ppm

3.26. ^{13}C NMR spectrum of complex (**^{pMe}CysNi)$_{L7}$**

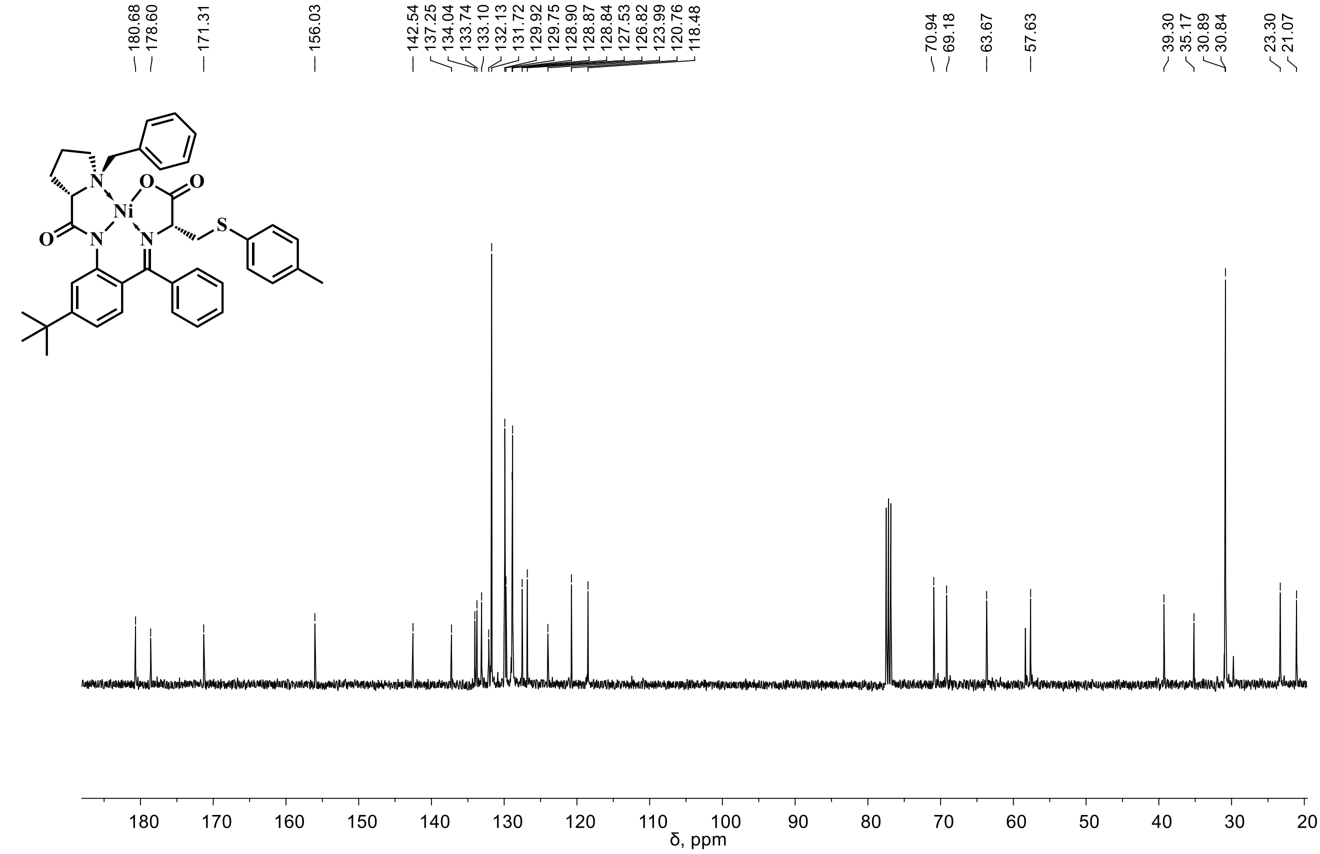

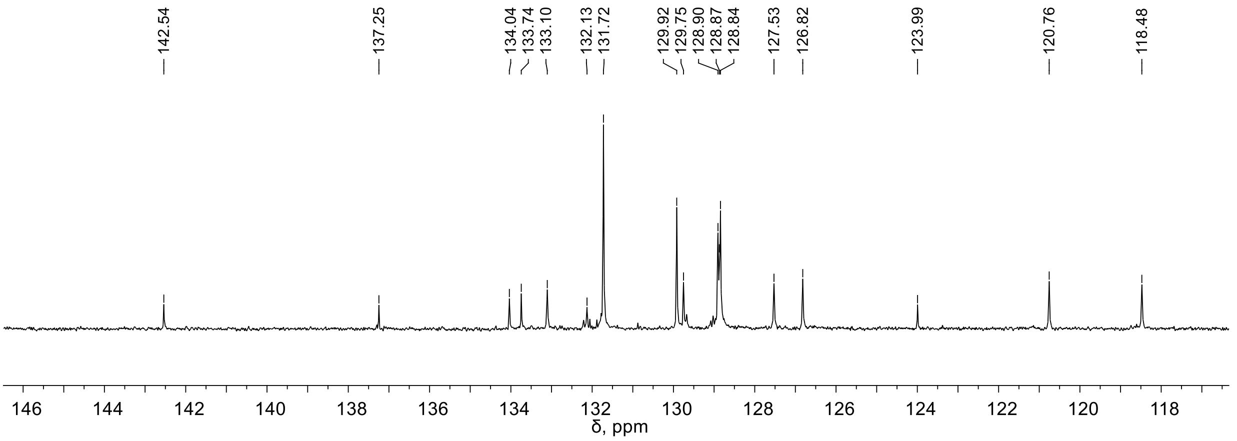

3.27. ^{1}H NMR spectrum of complex **(^{oBr}CysNi)$_{L7}$**

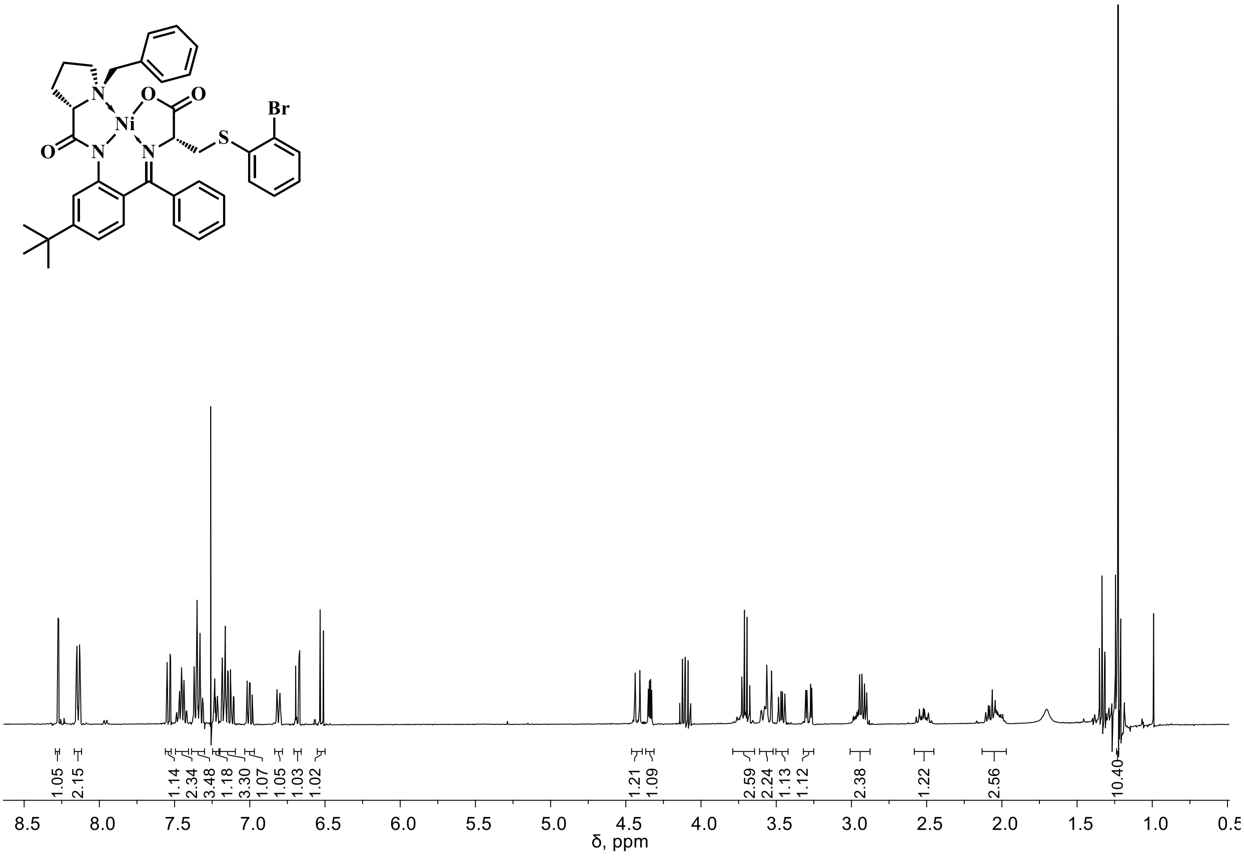

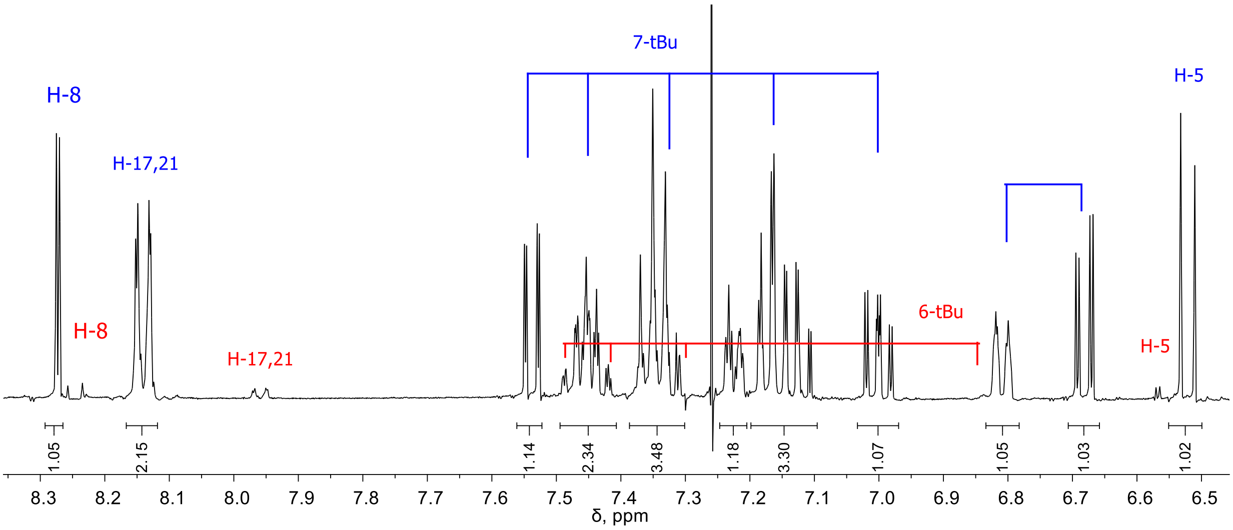

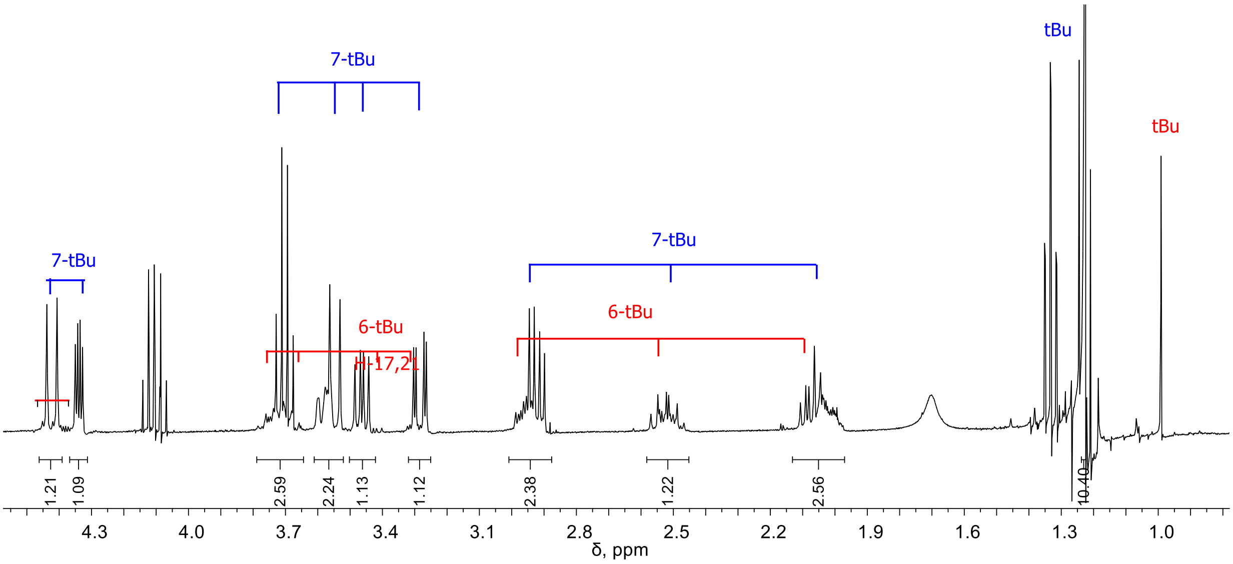

3.28. ^{13}C NMR spectrum of complex (**^{oBr}CysNi)$_{L7}$**

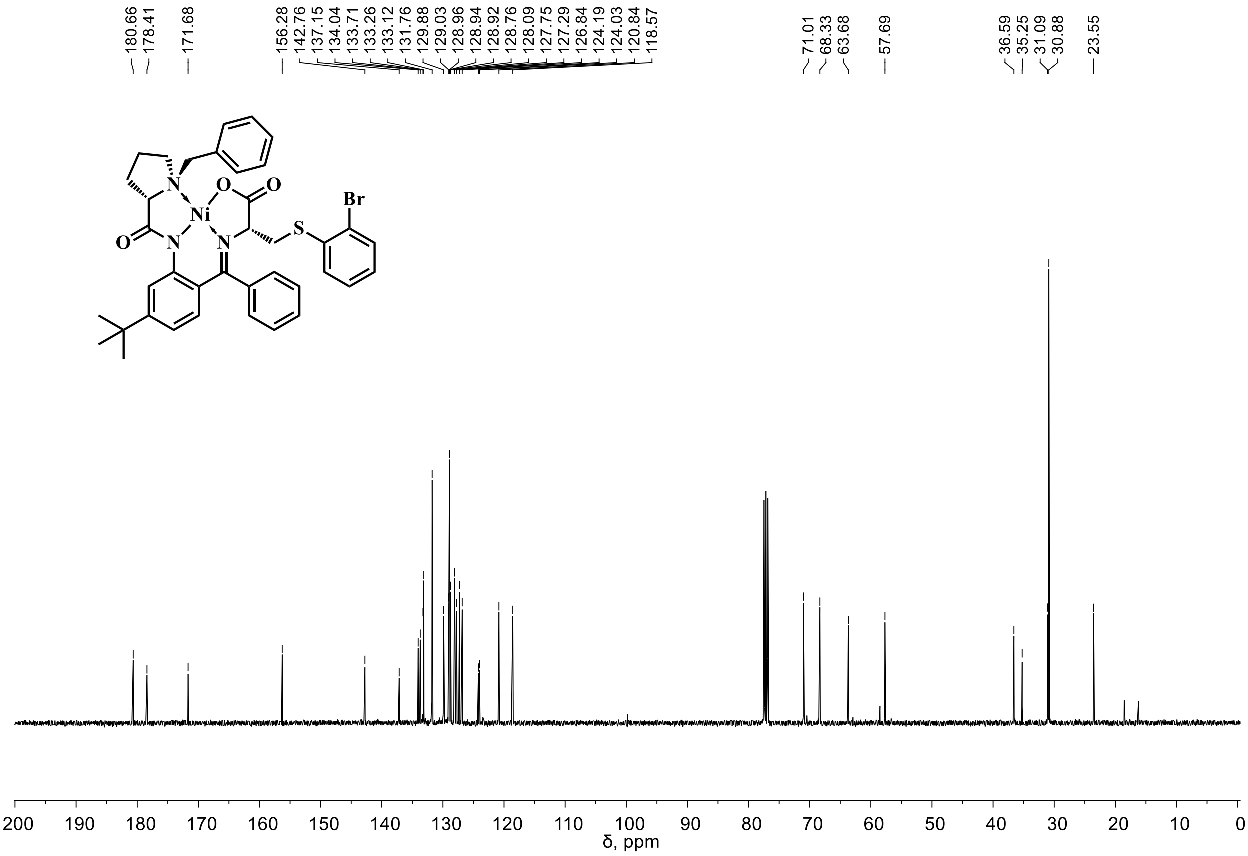

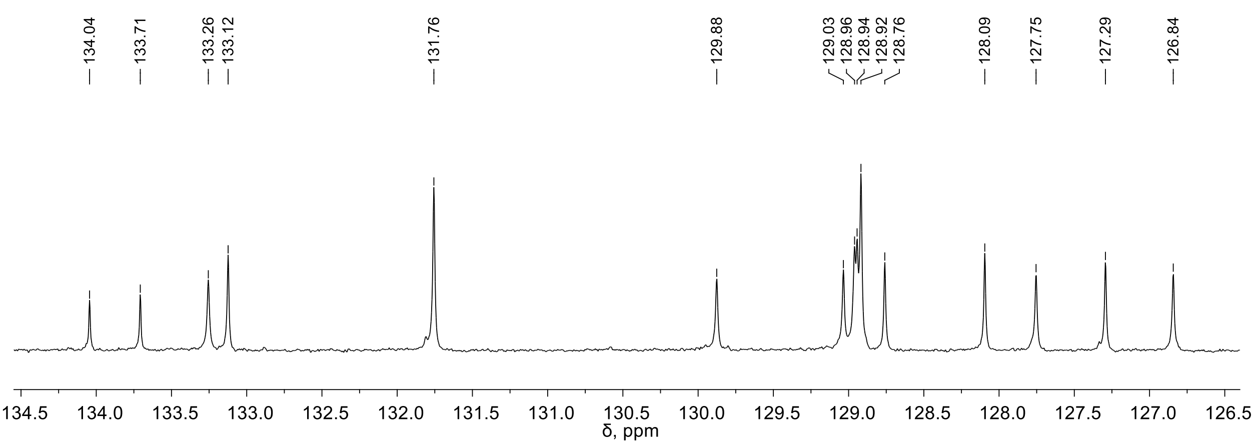

3.29. HSQC spectrum of complex ***(^{oBr}CysNi)$_{L7}$***

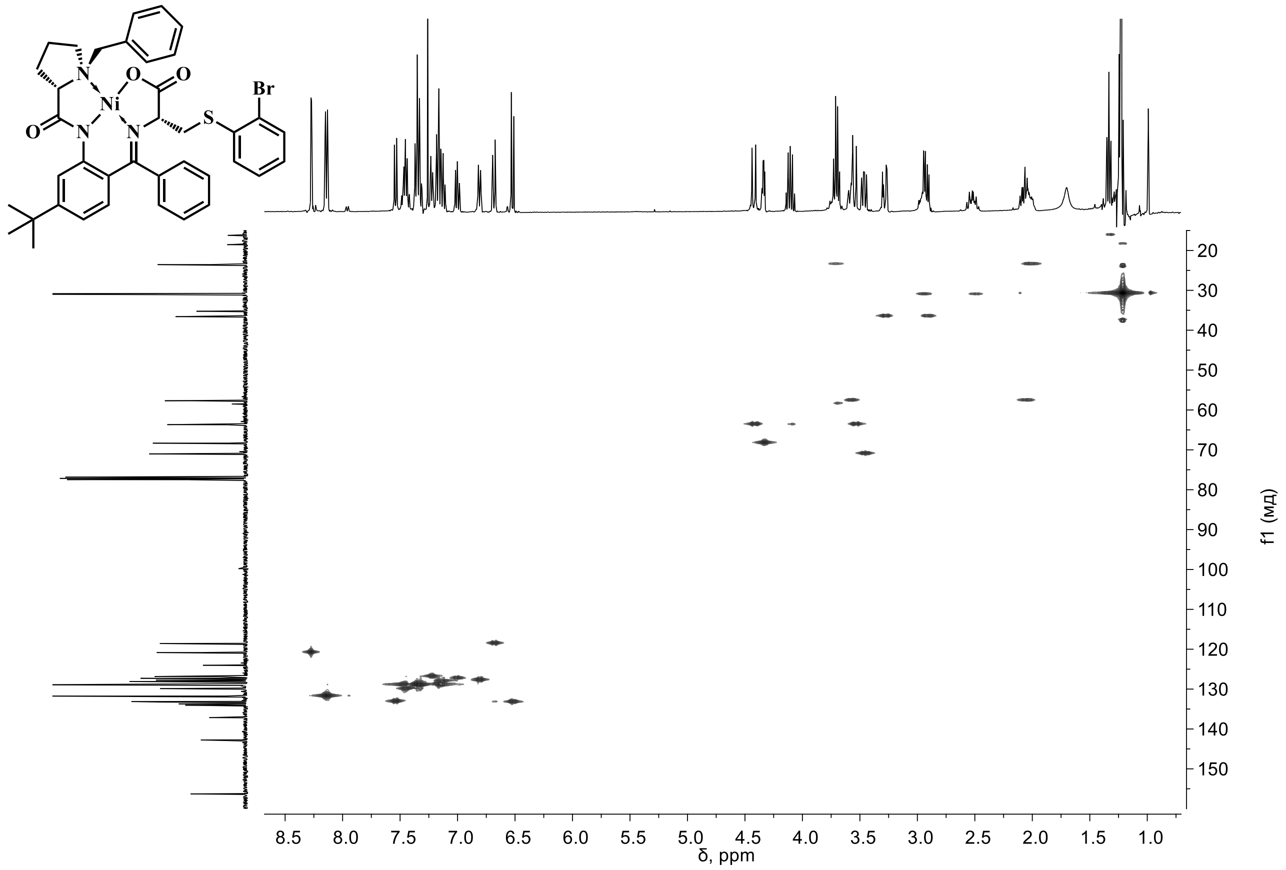

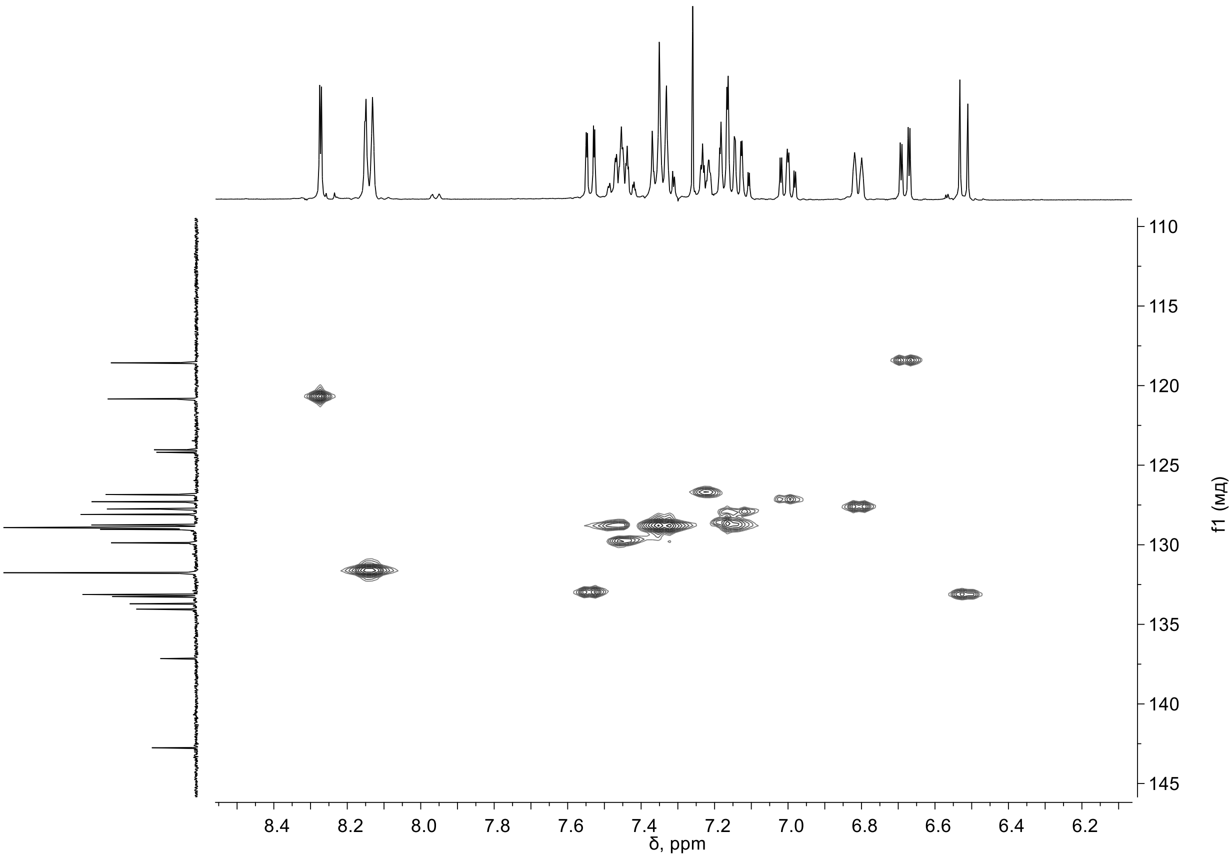
110
115
120
125
130
135
140
145
f1 (мд)
8.4
8.2
8.0
7.8
7.6
7.4
7.2
7.0
6.8
6.6
6.4
6.2
δ, ppm

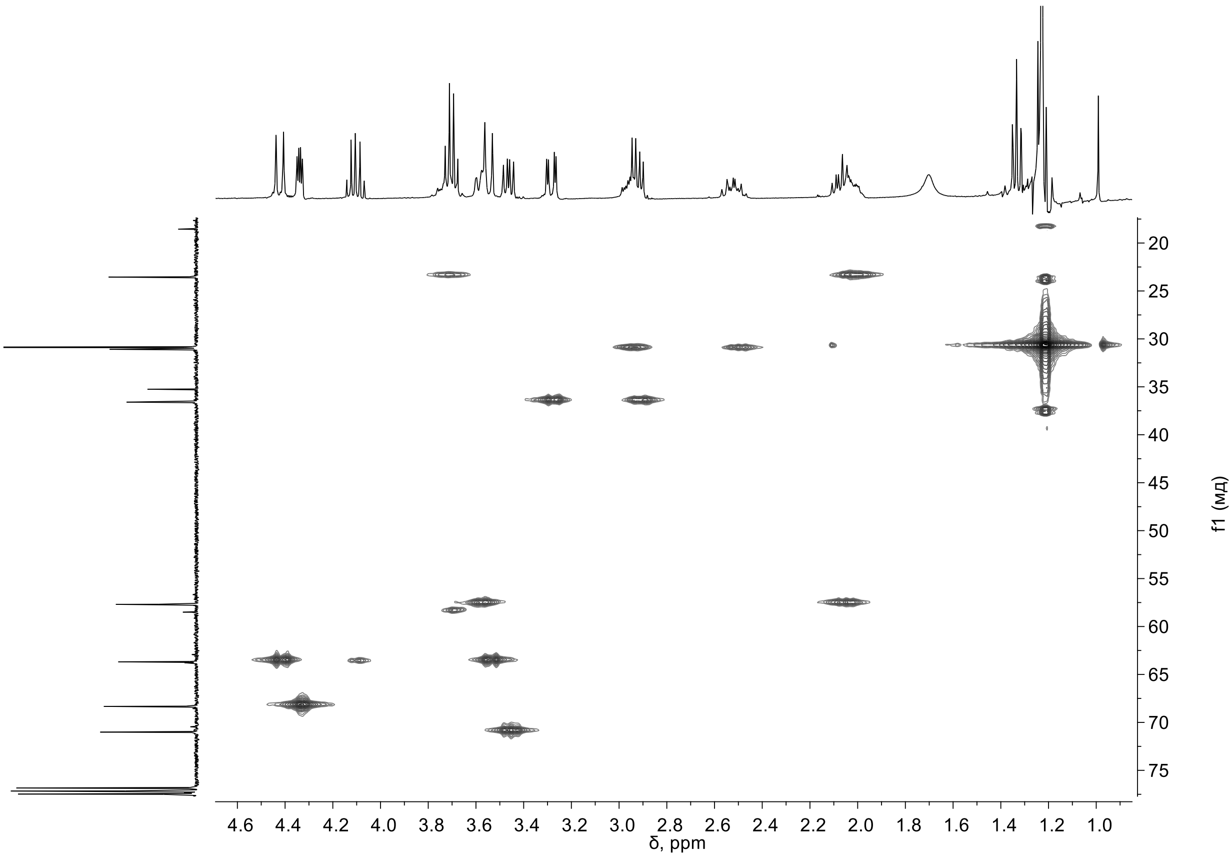
20
25
30
35
40
45
50
55
60
65
70
75
f1 (мд)
4.6
4.4
4.2
4.0
3.8
3.6
3.4
3.2
3.0
2.8
2.6
2.4
2.2
2.0
1.8
1.6
1.4
1.2
1.0
δ, ppm

3.30. HMBC spectrum of complex (^{oBr}CysNi)$_{L7}$

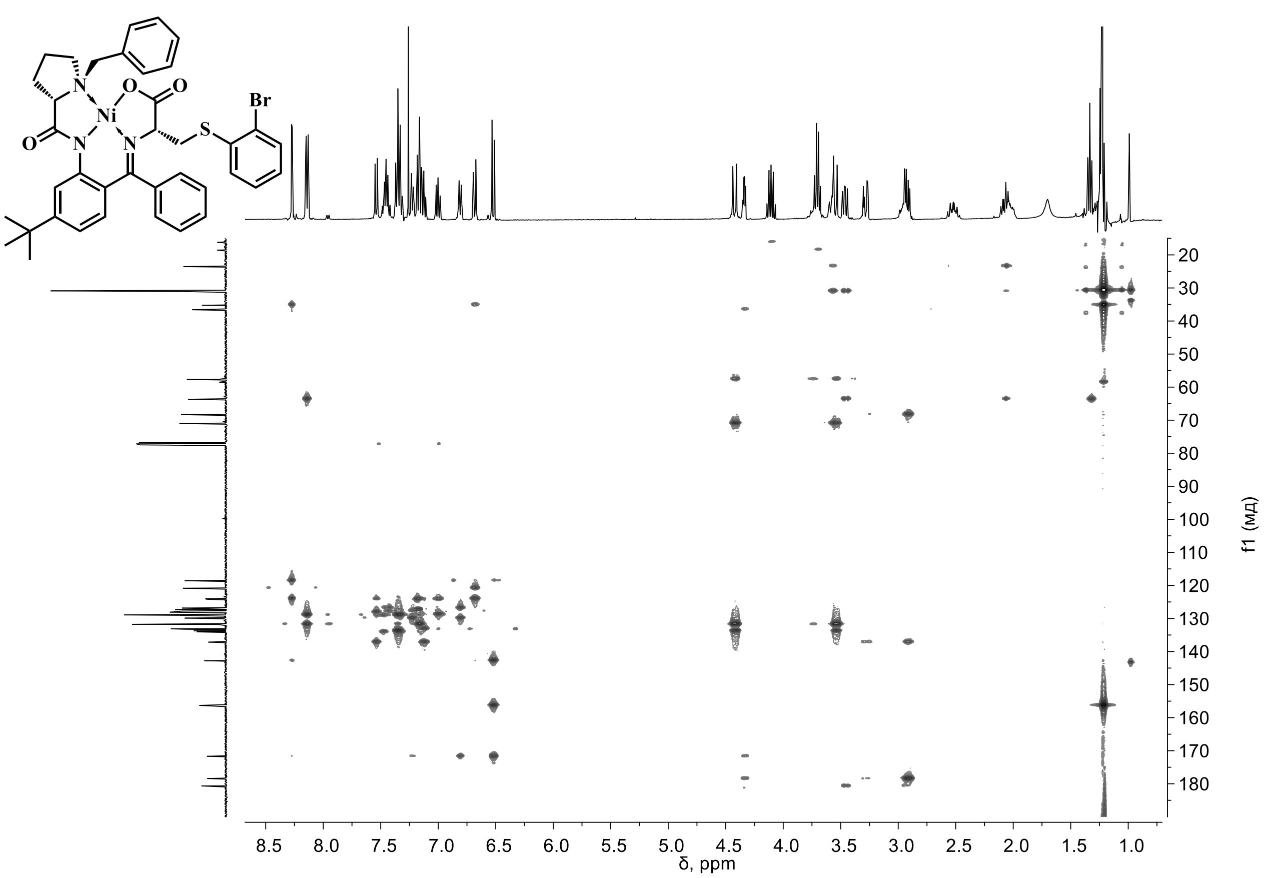

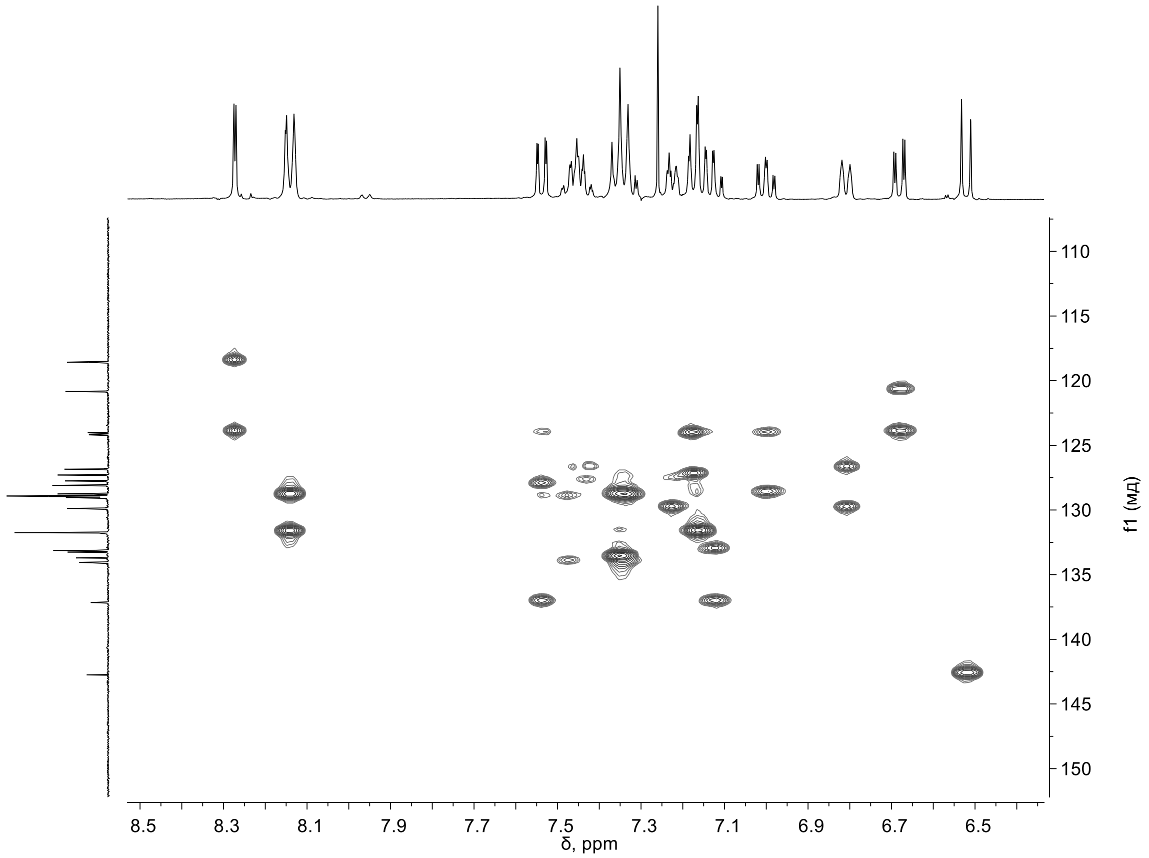

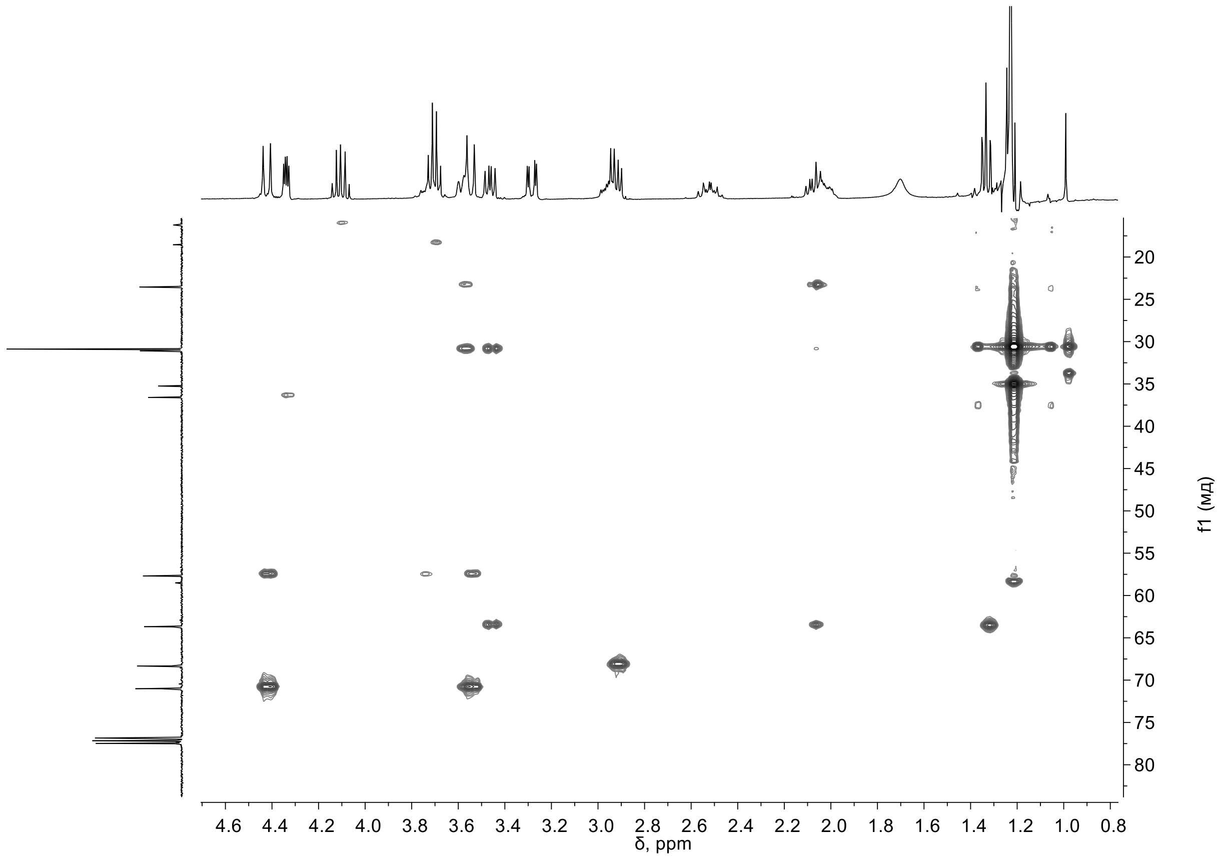

3.31. COSY spectrum of complex **(^{oBr}CysNi)$_{L7}$**

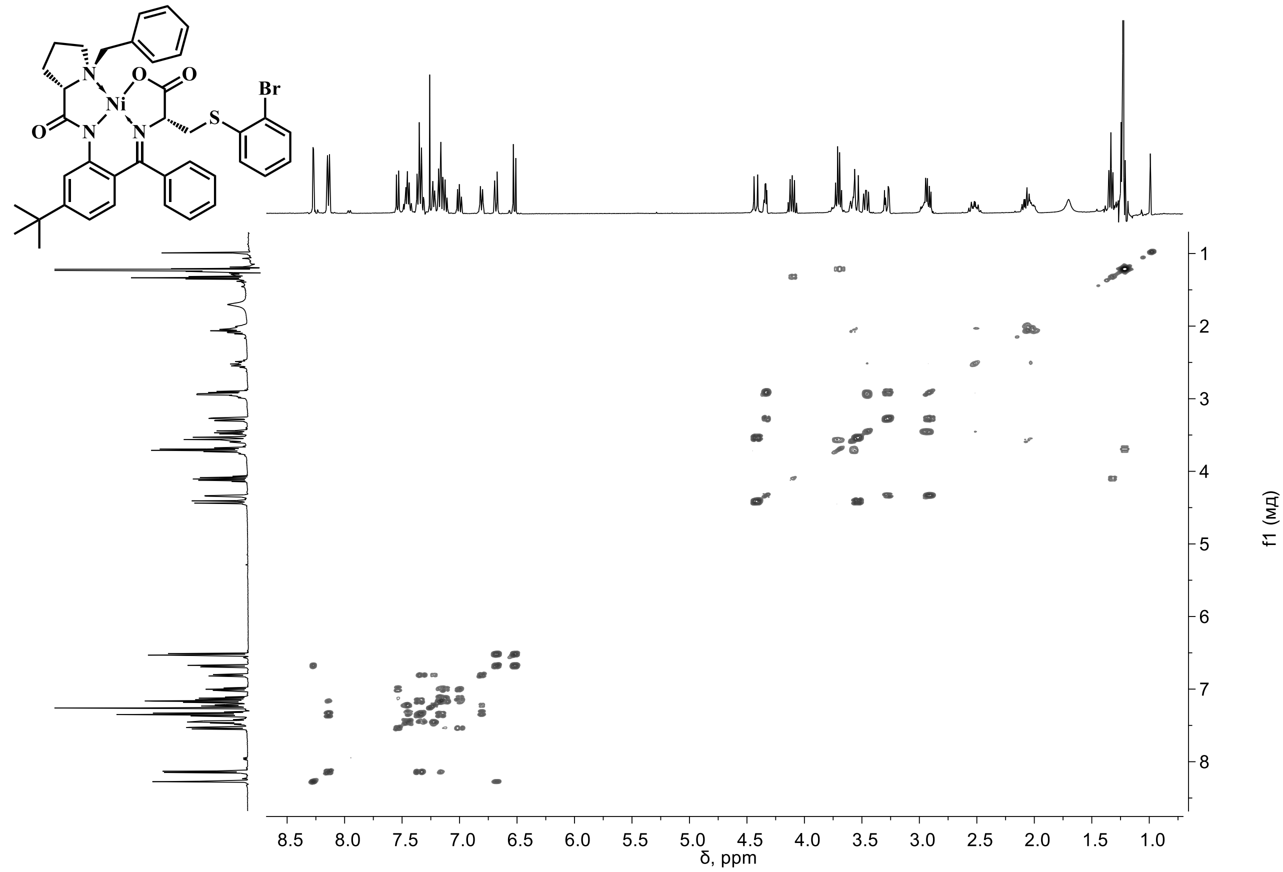

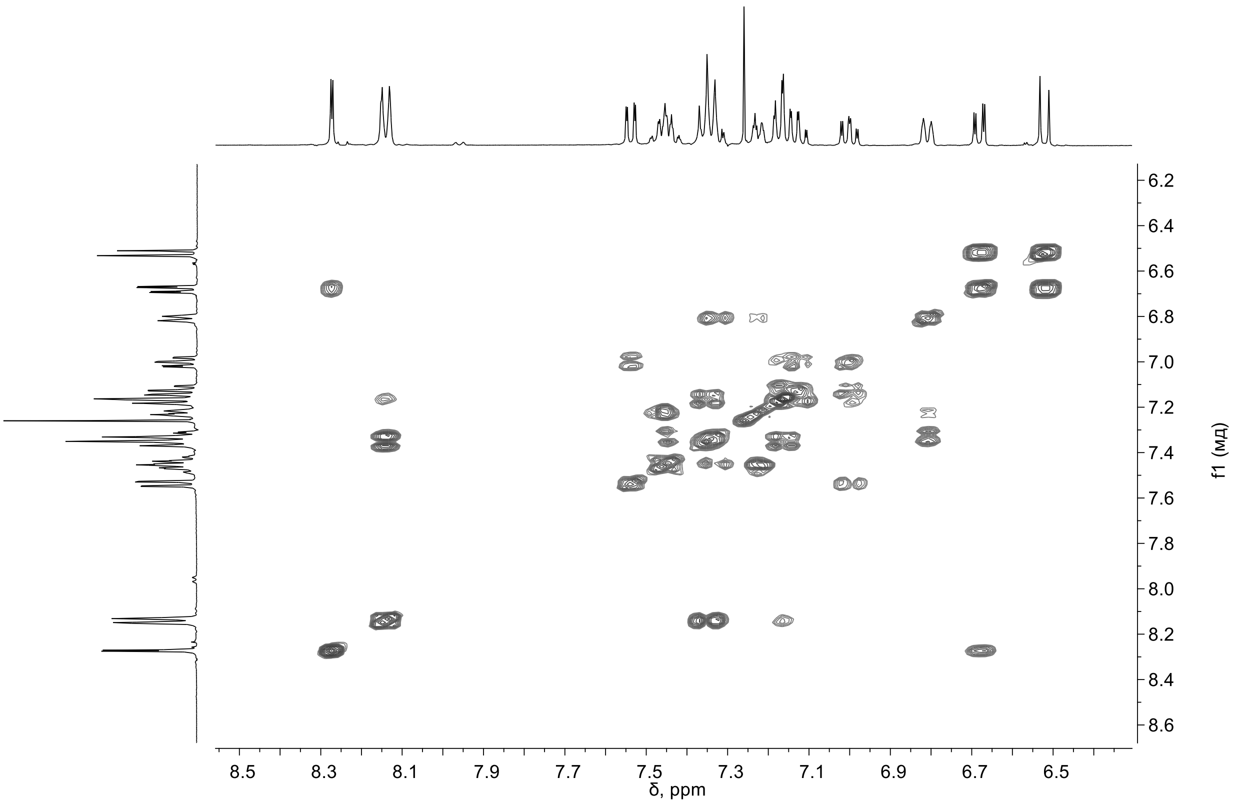
8.5
8.3
8.1
7.9
7.7
7.5
7.3
7.1
6.9
6.7
6.5
δ, ppm
6.2
6.4
6.6
6.8
7.0
7.2
7.4
7.6
7.8
8.0
8.2
8.4
8.6
f1 (мд)

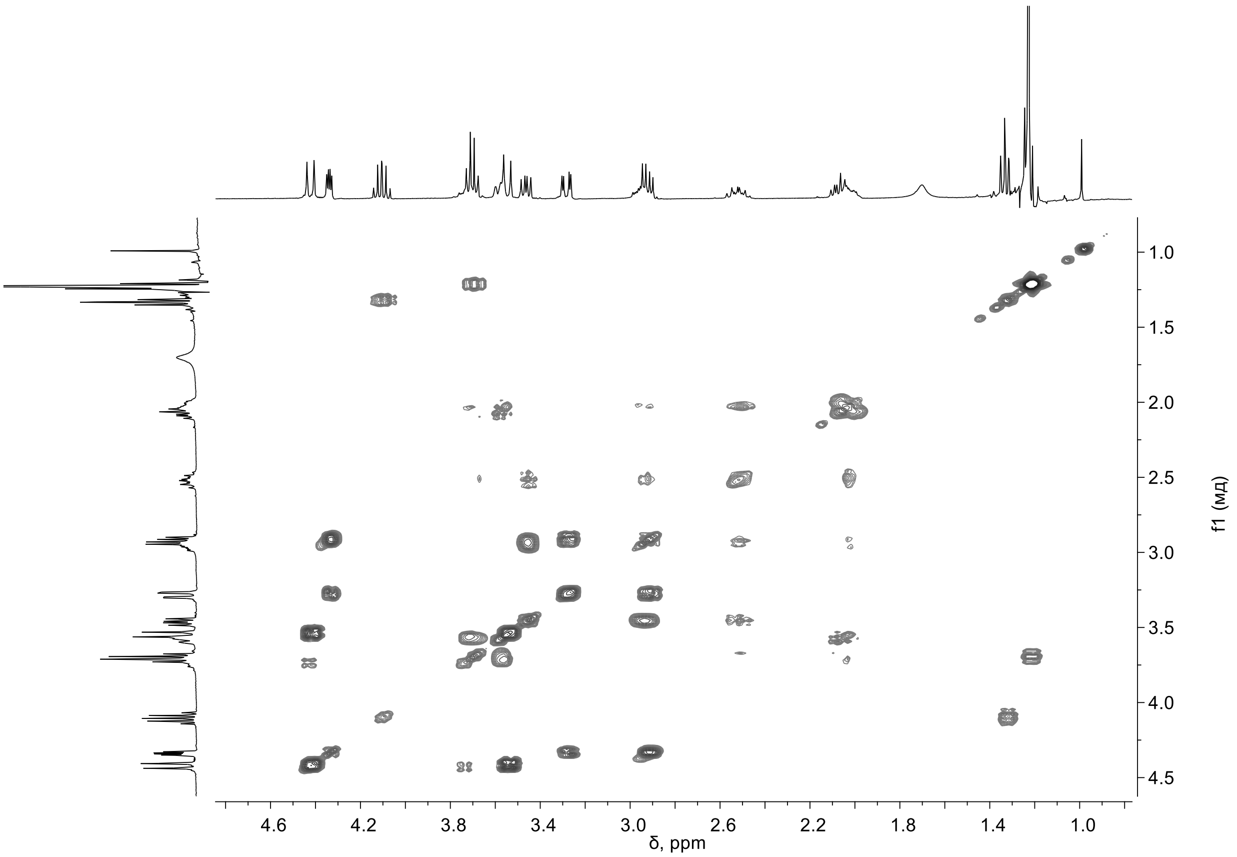
4.6
4.2
3.8
3.4
3.0
2.6
2.2
1.8
1.4
1.0
δ, ppm
1.0
1.5
2.0
2.5
3.0
3.5
4.0
4.5
f1 (мд)

3.32. *NOESY spectrum of complex **(^{oBr}CysNi)$_{L7}$***

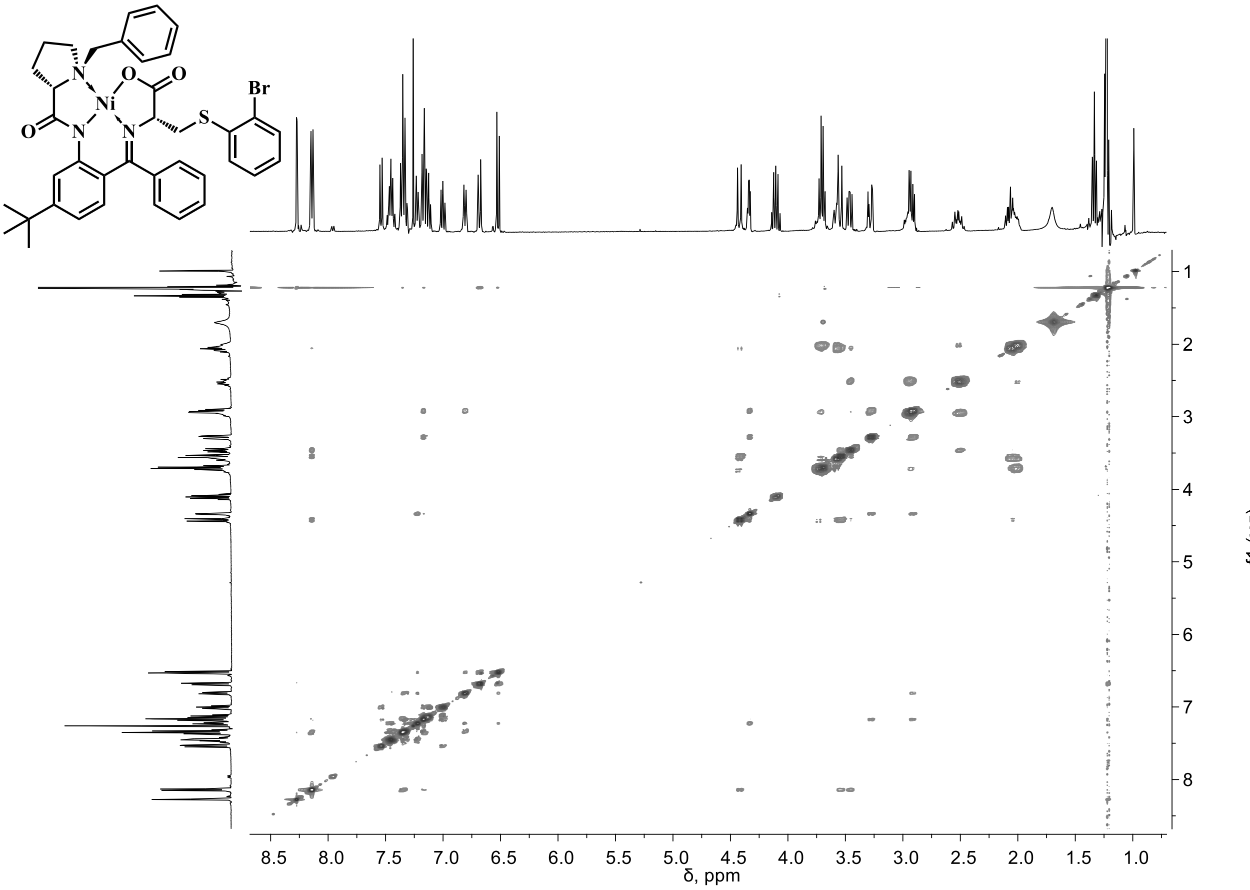

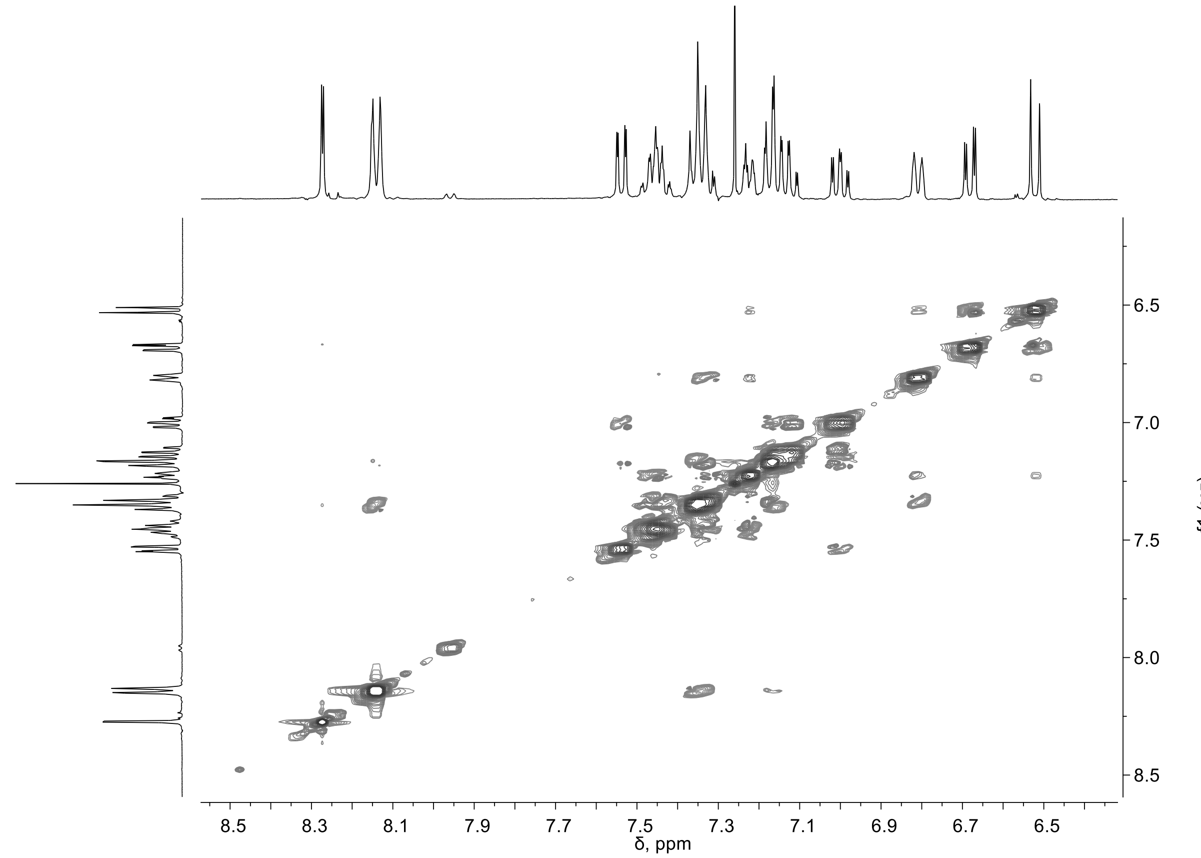

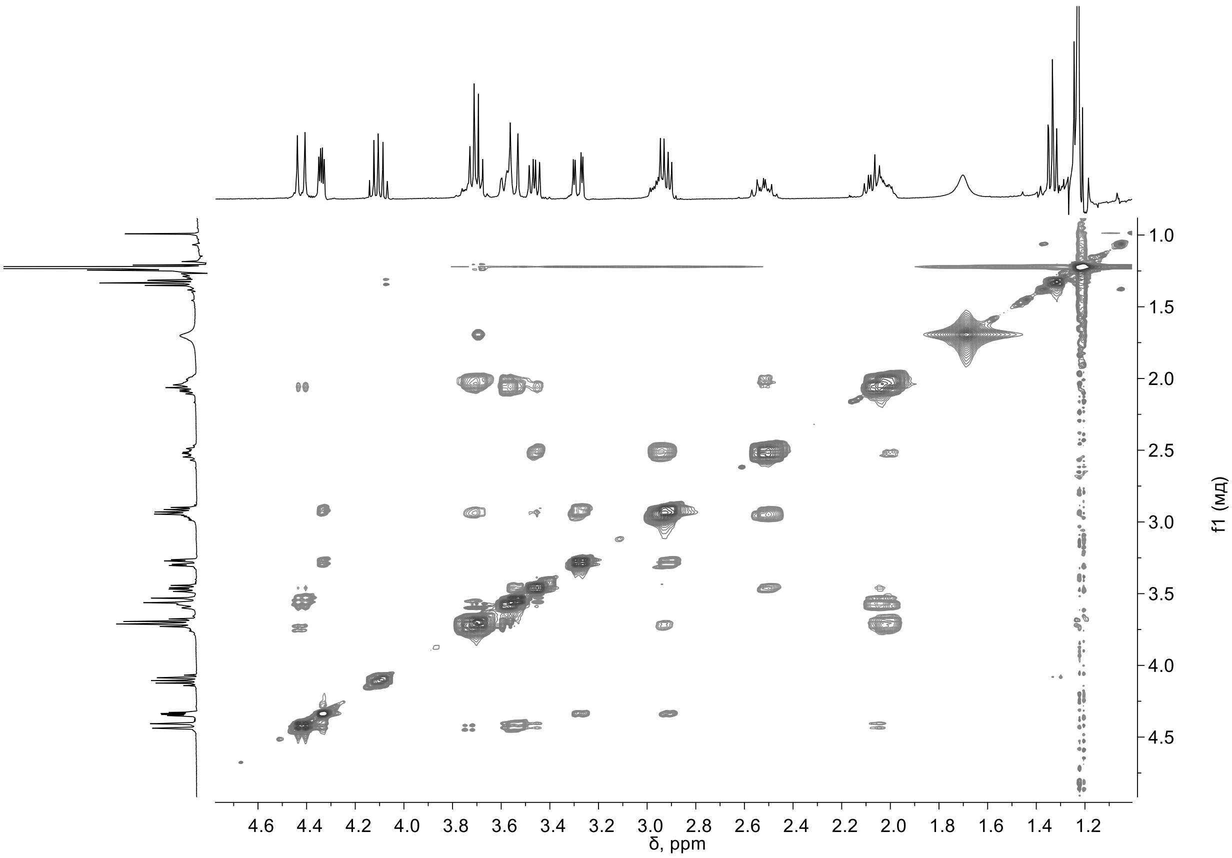

f1 (мд)
δ, ppm

3.33. ^{1}H NMR spectrum of complex **(^{pBr}CysNi)$_{L7}$**

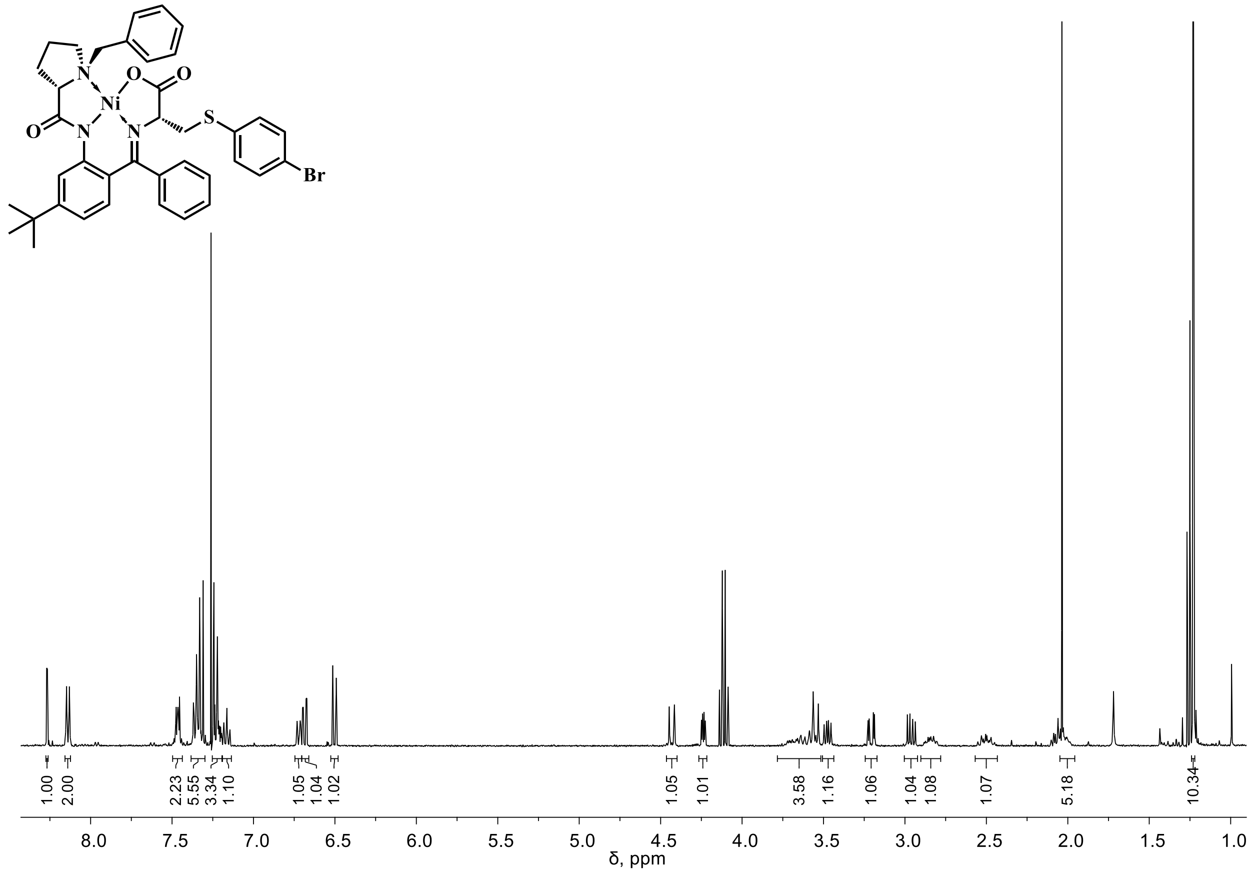

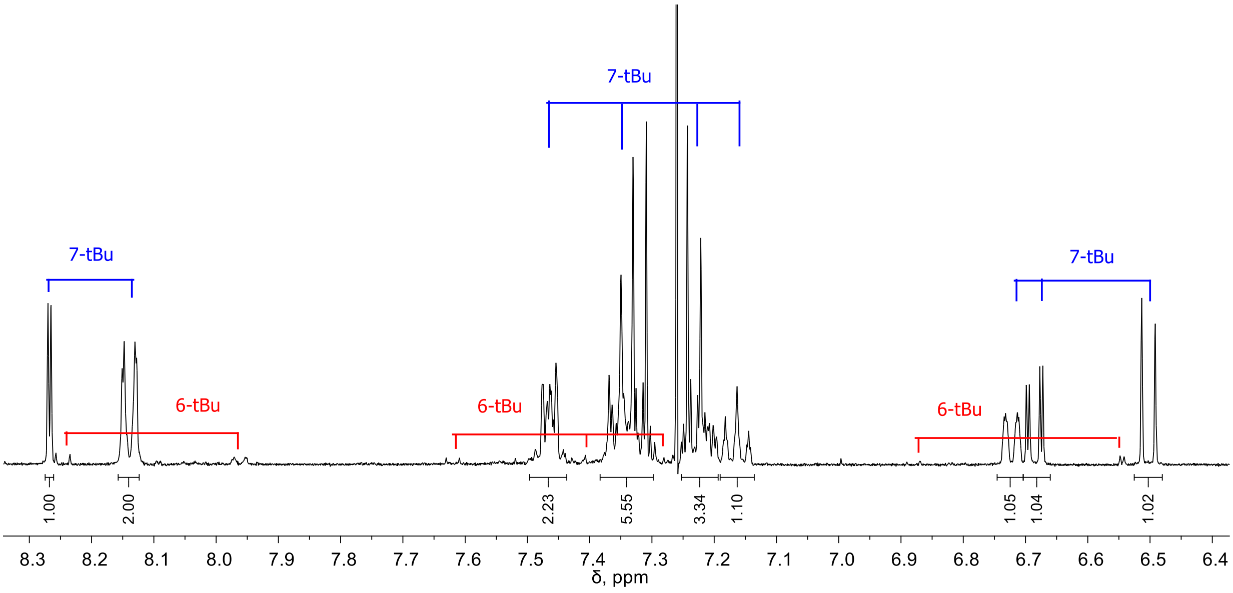

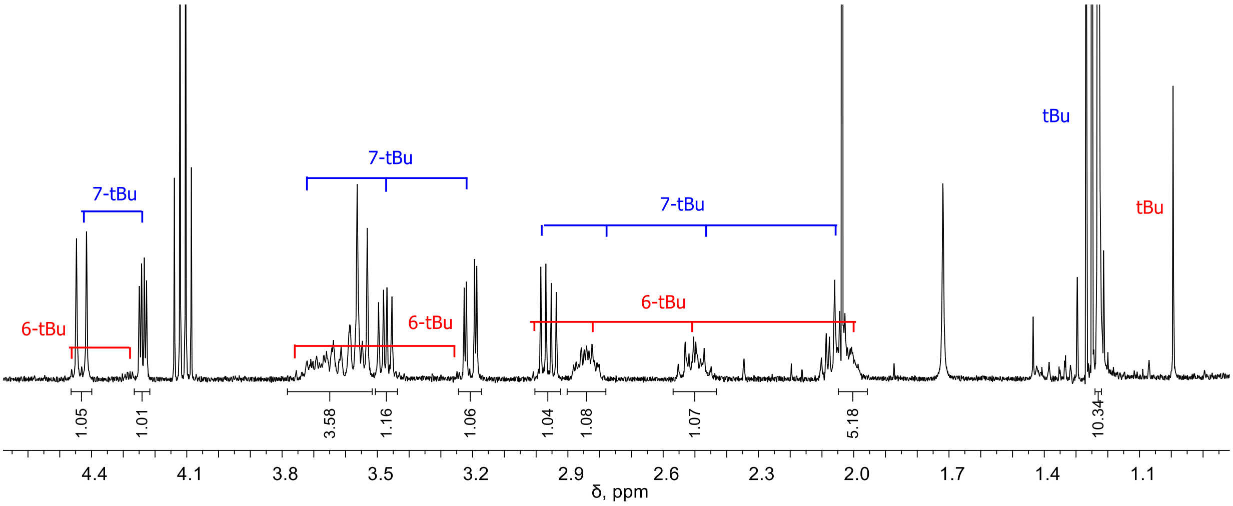

3.34. ^{13}C NMR spectrum of complex **(^{pBr}CysNi)$_{L7}$**

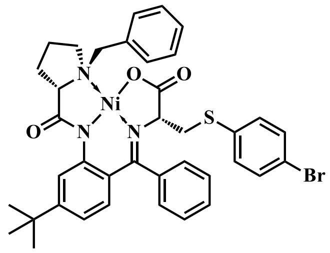

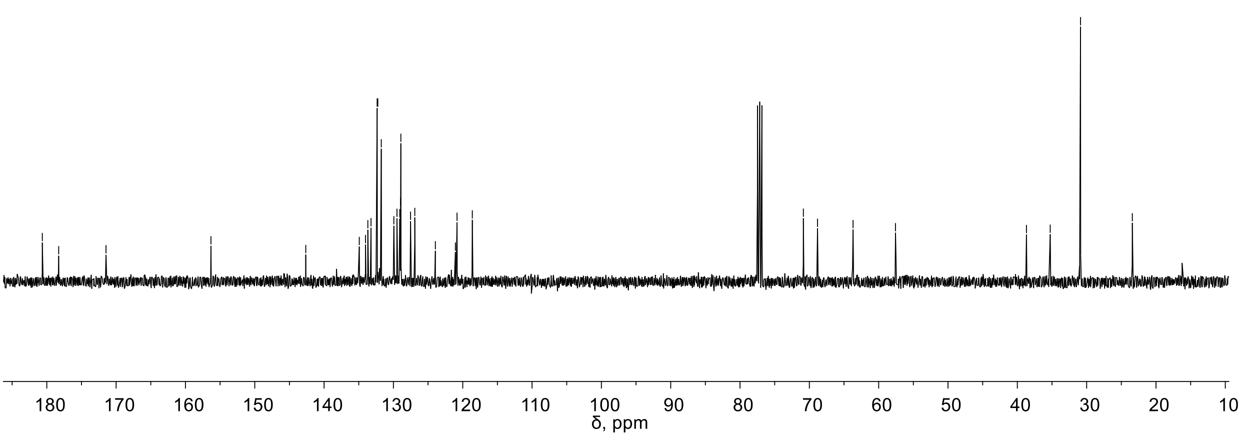

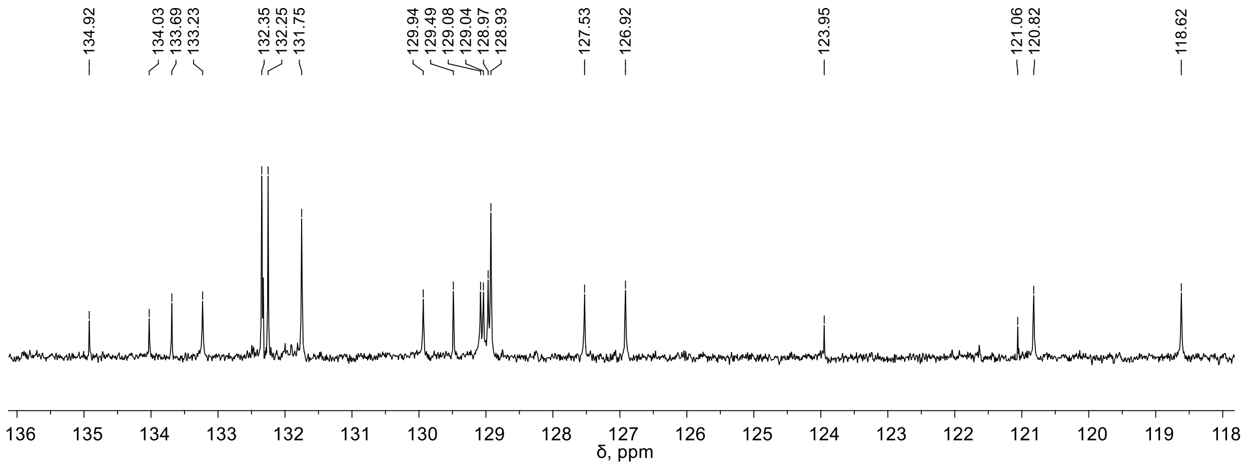

3.35. ^{1}H NMR spectrum of L-S-tolylcysteine

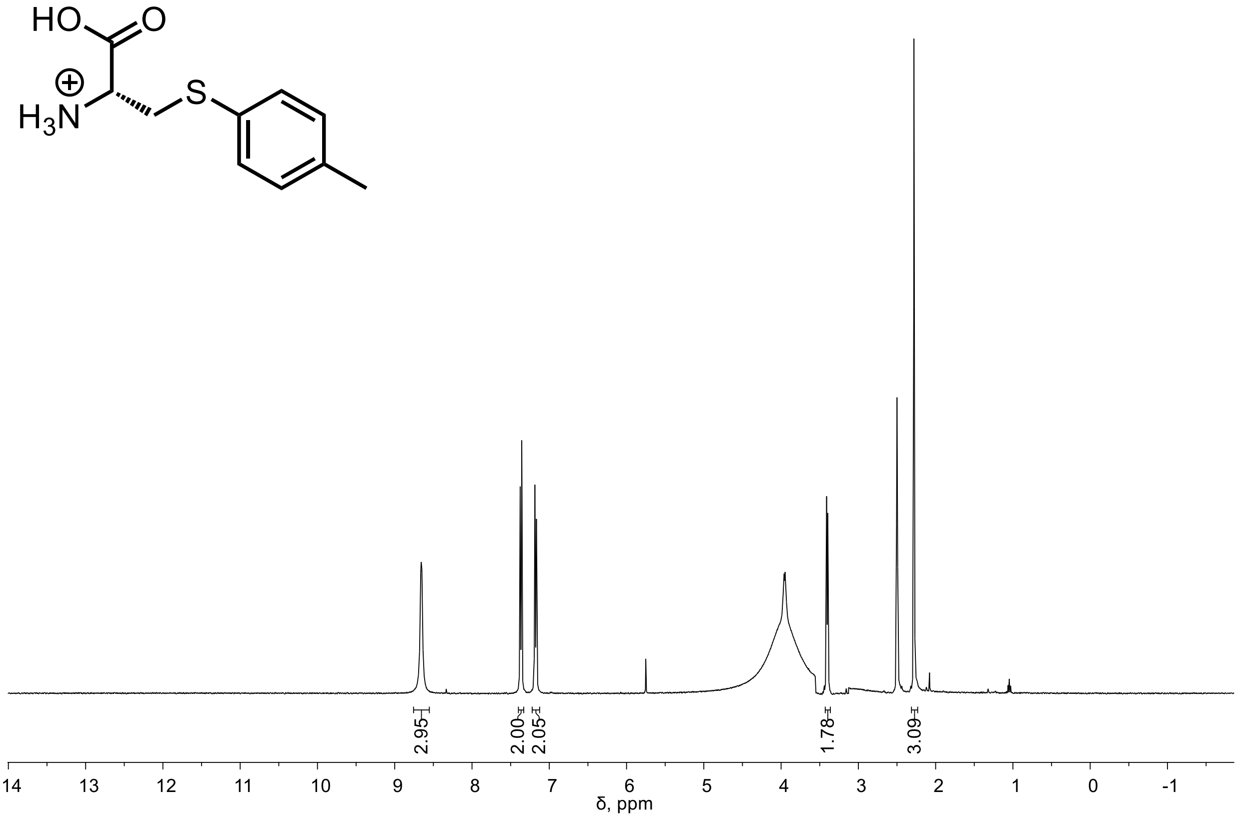

3.36. ^{13}C NMR spectrum of L-S-tolylcysteine

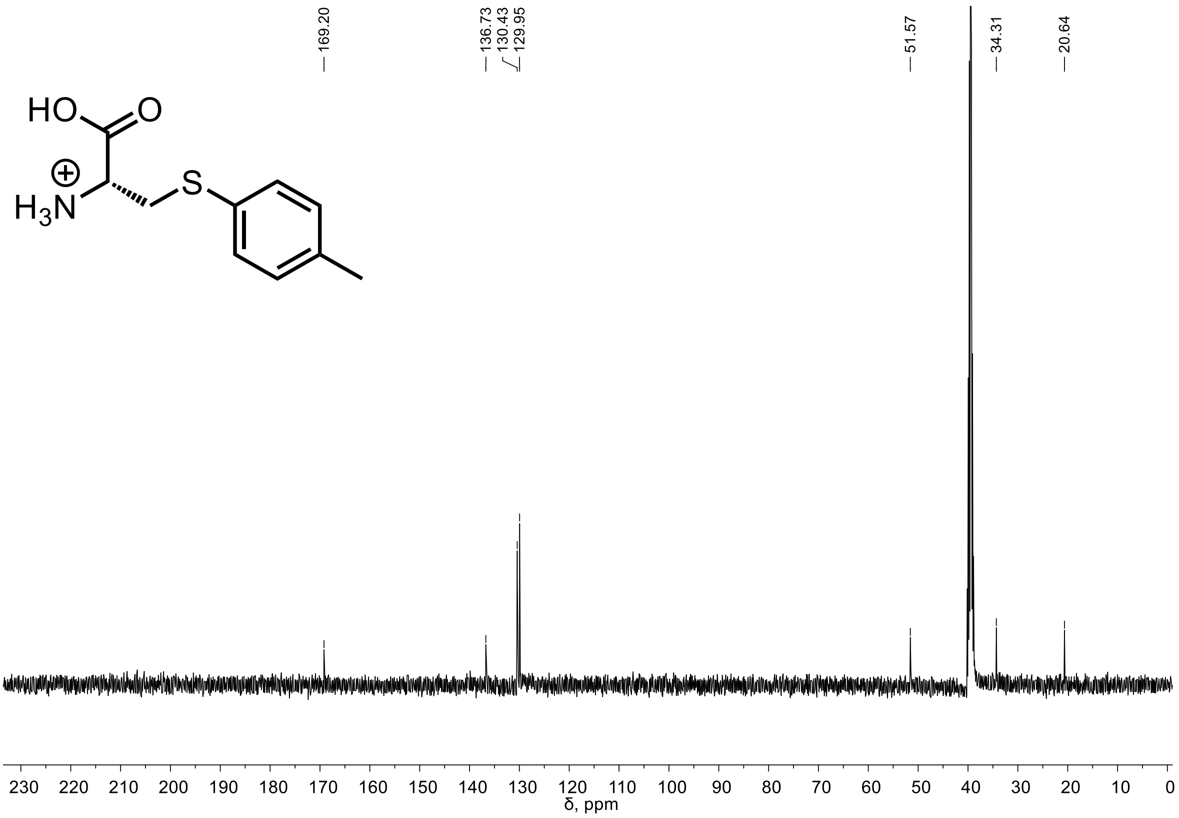

4. Mass spectra for all compounds

4.1. GC–MS data for 2-benzoyl-5-tert-butylaniline

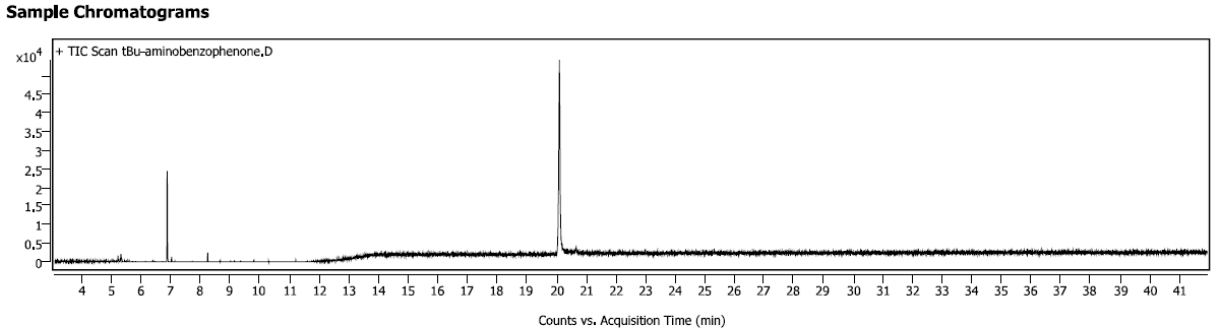

+ Scan (rt: 20.102 min) Sub

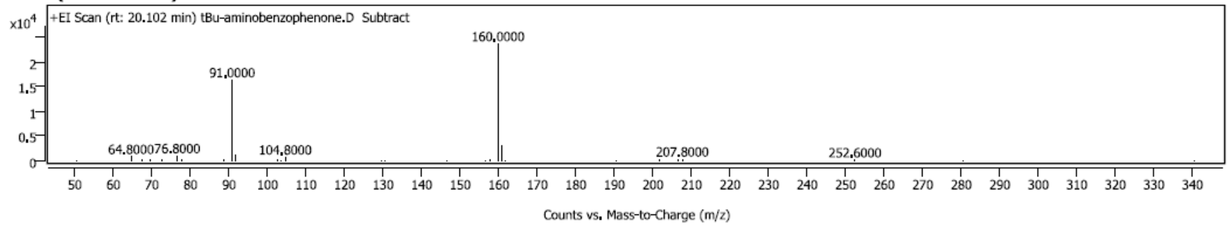

Spectrum Peaks

m/z	Z	Abund	Abund %	m/z (Calc)	Diff (ppm)	Ion Species	Formula	Ion Type
64,8000		976	4,13					
67,7000	2	296	1,25					
69,8000		295	1,24					
72,8000		283	1,20					
76,8000		1143	4,83					
77,8000		258	1,09					
88,8000		305	1,29					
91,0000	1	16386	69,23					
91,9000	1	1306	5,52					
102,7000	2	270	1,14					
104,8000		858	3,62					
157,9000		416	1,76					
160,0000	1	23669	100,00					
161,0000	1	3128	13,22					
201,8000		268	1,13					
206,8000		315	1,33					
207,8000		477	2,02					
252,6000	2	296	1,25					

+ Scan (rt: 6.884 min) Benzoic acid, methyl ester; C8H8O2

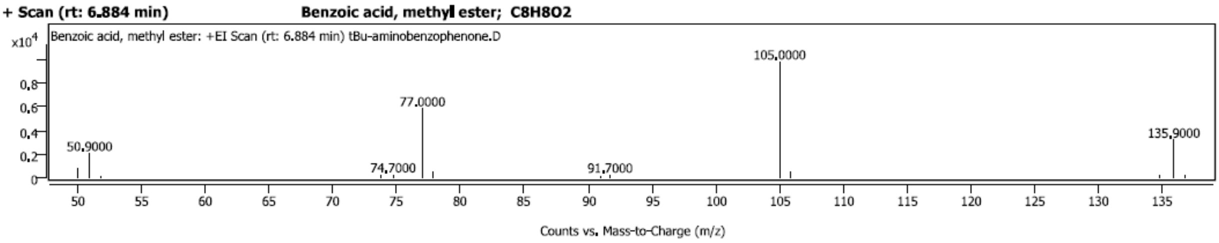

Spectrum Peaks

m/z	Z	Abund	Abund %	m/z (Calc)	Diff (ppm)	Ion Species	Formula	Ion Type
50,0000		856	8,72					
50,9000		2079	21,18					
51,8000		191	1,95					
73,7000	2	261	2,66					
74,7000	2	271	2,76					
77,0000		5854	59,65					
77,8000		506	5,16					
91,0000		187	1,91					
91,7000	2	248	2,53					
105,0000		9815	100,00					
105,8000		520	5,30					
134,8000		208	2,12					
135,9000	1	3210	32,71					
136,8000	1	217	2,21					

4.2. ESI-HRMS data for compound **L7**

Acquisition Parameter

Source Type	ESI	Ion Polarity	Positive	Set Nebulizer	1.0 Bar
Focus	Not active			Set Dry Heater	200 °C
Scan Begin	50 m/z	Set Capillary	4500 V	Set Dry Gas	4.0 l/min
Scan End	1600 m/z	Set End Plate Offset	-500 V	Set Divert Valve	Waste

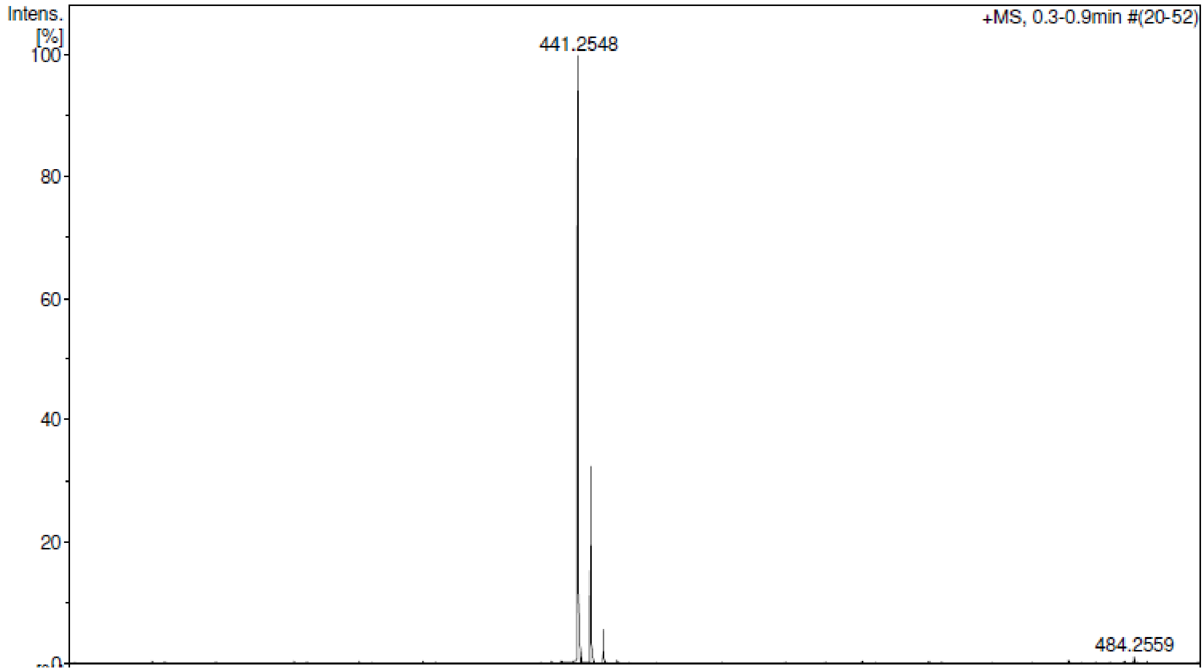

4.3. ESI-HRMS data for complex **(GlyNi)$_{L7}$**

Acquisition Parameter

Source Type	ESI	Ion Polarity	Positive	Set Nebulizer	1.0 Bar
Focus	Not active			Set Dry Heater	200 °C
Scan Begin	50 m/z	Set Capillary	4500 V	Set Dry Gas	4.0 l/min
Scan End	1600 m/z	Set End Plate Offset	-500 V	Set Divert Valve	Waste

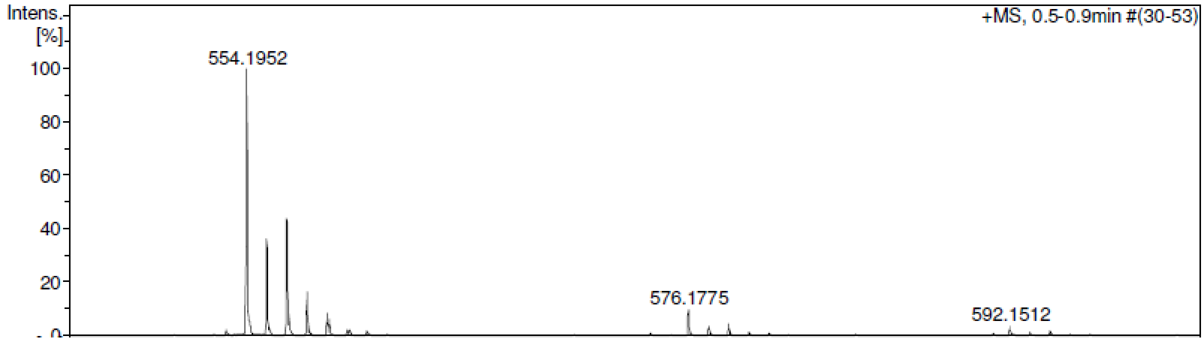

4.4. *ESI-HRMS data for complex* ***(2R)-(SerNi)**$_{L7}$*

Acquisition Parameter

Source Type	ESI	Ion Polarity	Positive	Set Nebulizer	1.0 Bar
Focus	Not active			Set Dry Heater	200 °C
Scan Begin	50 m/z	Set Capillary	4500 V	Set Dry Gas	4.0 l/min
Scan End	1600 m/z	Set End Plate Offset	-500 V	Set Divert Valve	Waste

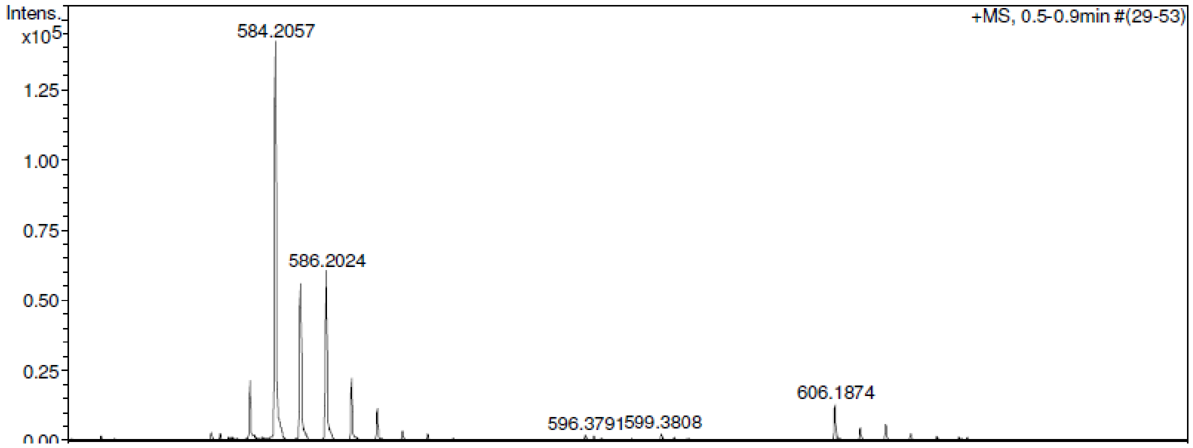

4.5. *ESI-HRMS data for complex* ***(2S)-(SerNi)**$_{L7}$*

Acquisition Parameter

Source Type	ESI	Ion Polarity	Positive	Set Nebulizer	1.0 Bar
Focus	Not active			Set Dry Heater	200 °C
Scan Begin	50 m/z	Set Capillary	4500 V	Set Dry Gas	4.0 l/min
Scan End	1600 m/z	Set End Plate Offset	-500 V	Set Divert Valve	Waste

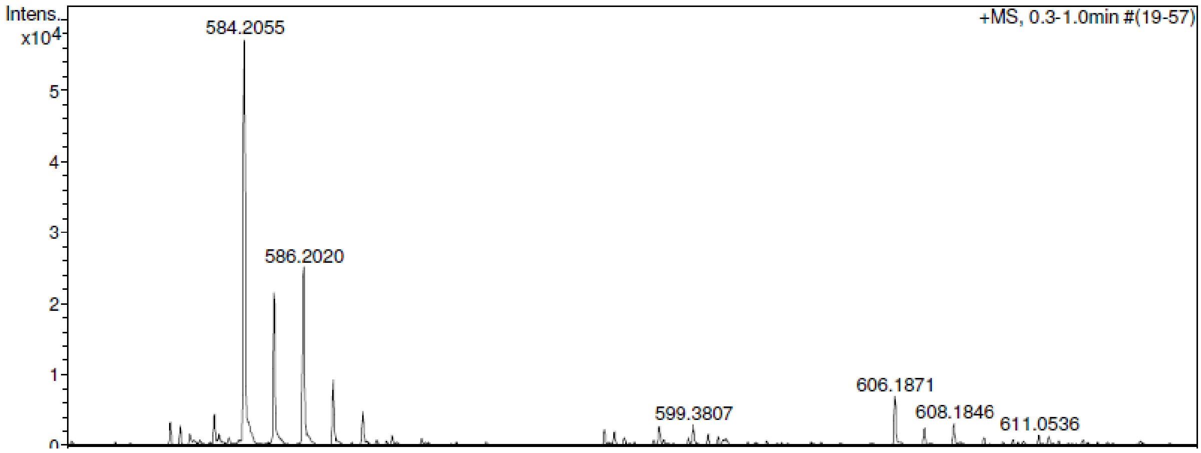

4.6. ESI-HRMS data for complex **(ΔAlaNi)$_{L7}$**

Acquisition Parameter

Source Type	ESI	Ion Polarity	Positive	Set Nebulizer	1.0 Bar
Focus	Not active			Set Dry Heater	200 °C
Scan Begin	50 m/z	Set Capillary	4500 V	Set Dry Gas	4.0 l/min
Scan End	1600 m/z	Set End Plate Offset	-500 V	Set Divert Valve	Waste

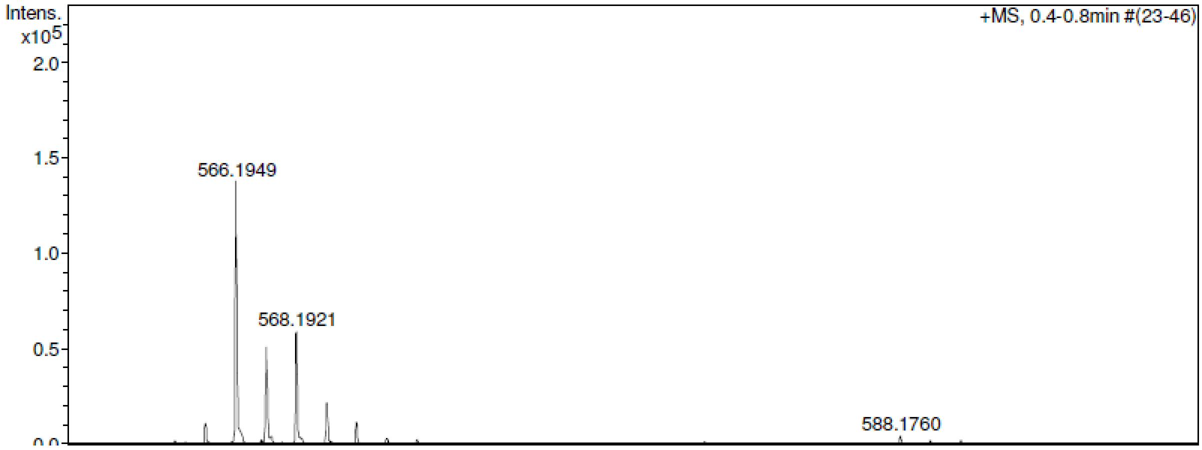

4.7. ESI-HRMS data for complex **(^{Bn}CysNi)$_{L7}$**

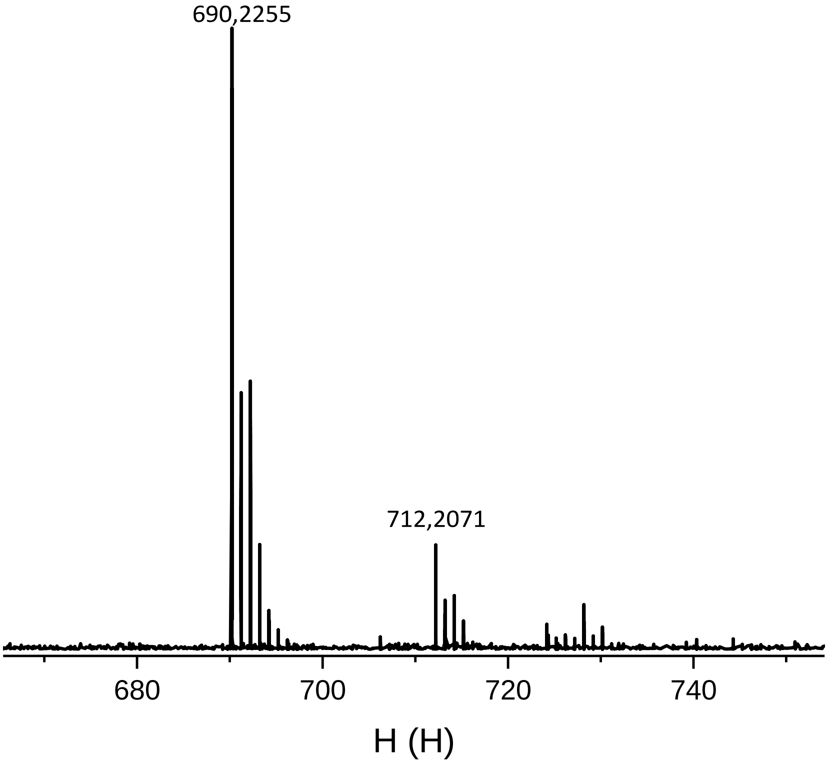

*4.8. ESI-HRMS data for complex (**^{Ph}CysNi)$_{L7}$***

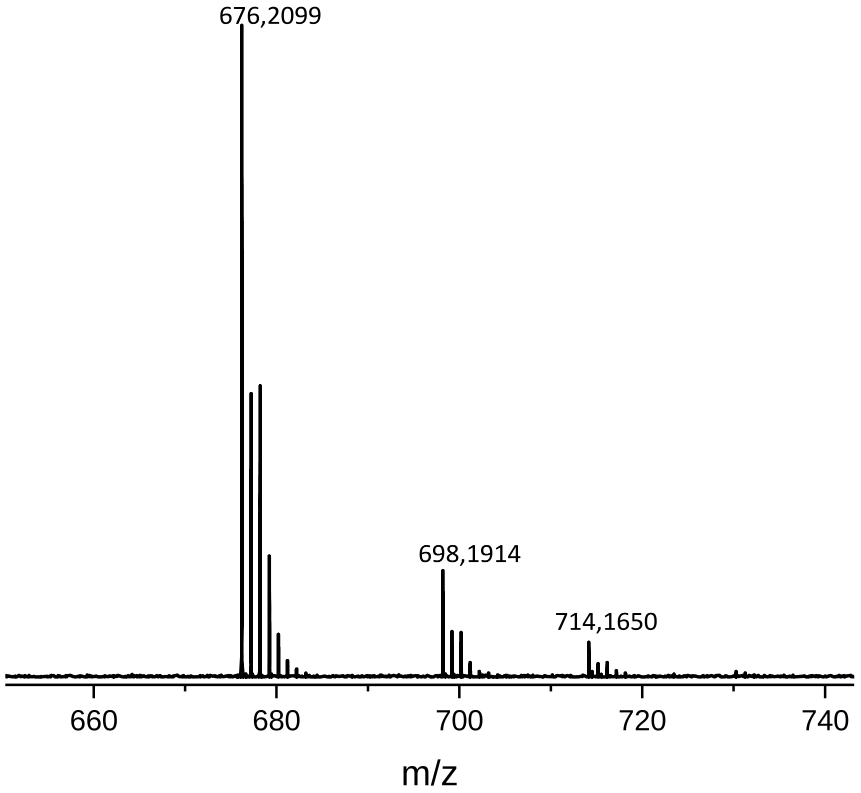

*4.9. ESI-HRMS data for complex (**^{pMe}CysNi)$_{L7}$***

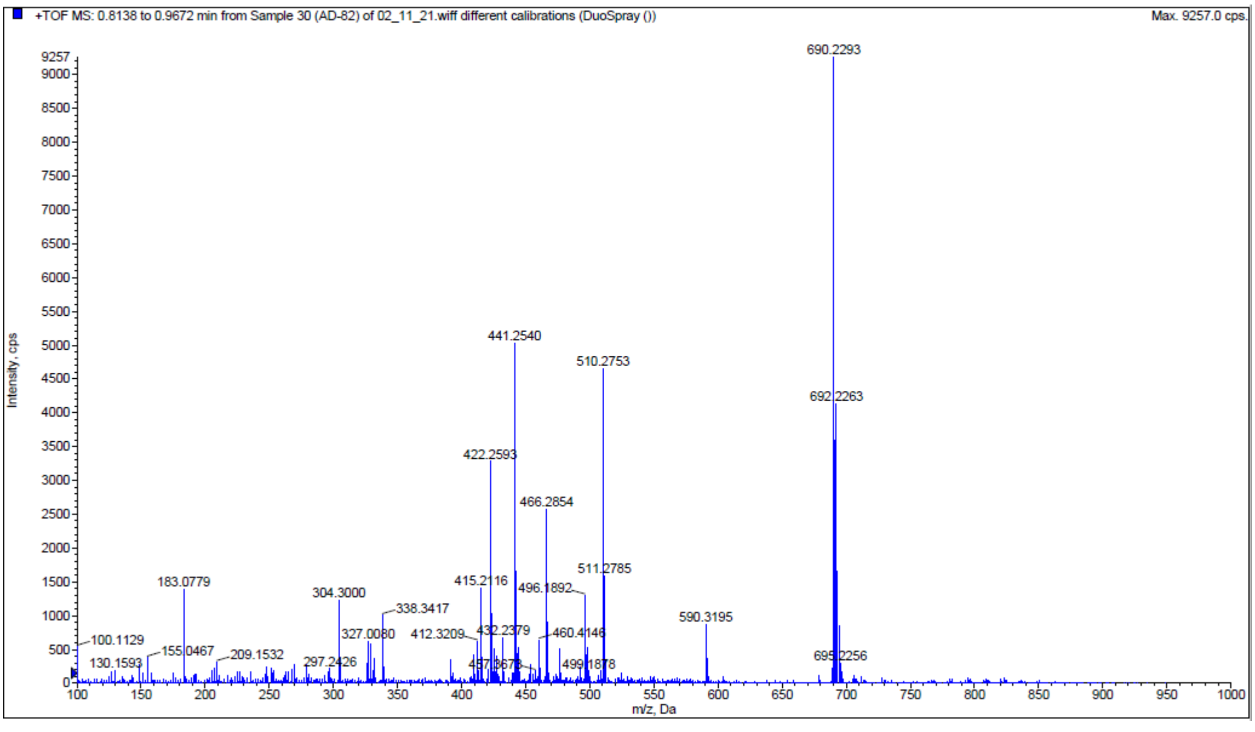

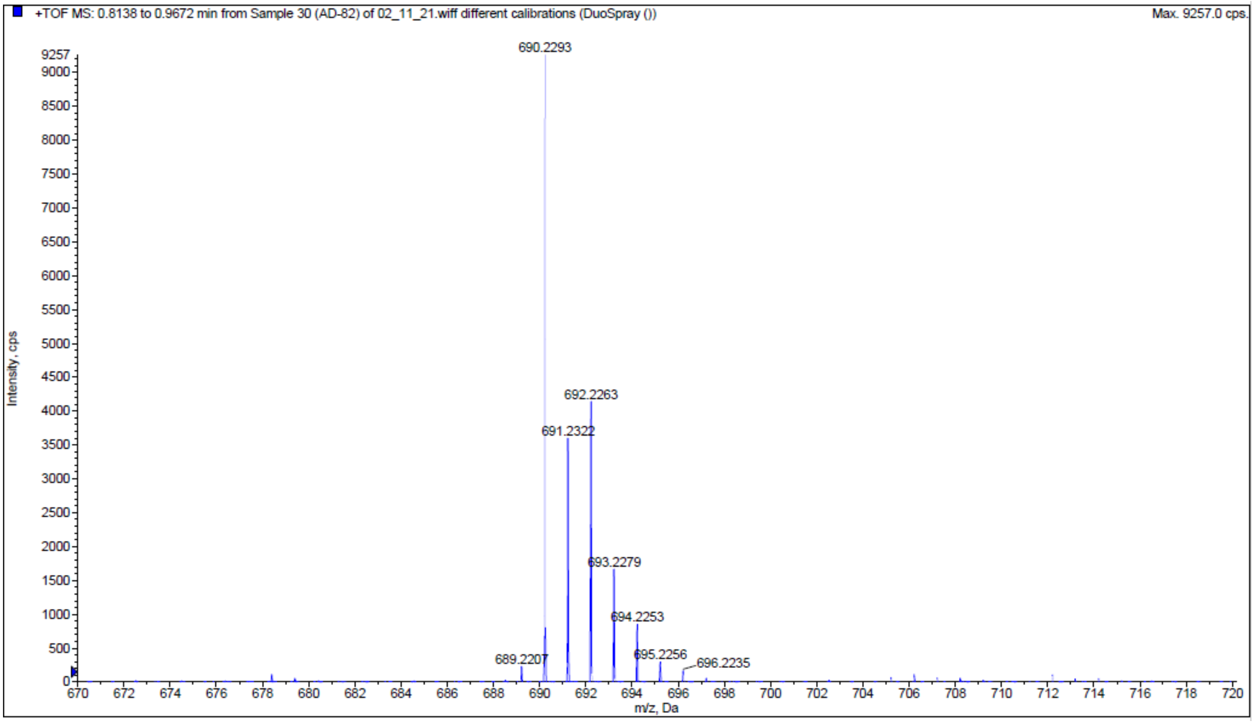

4.10. *ESI-HRMS data for complex* **(^{oBr}CysNi)$_{L7}$**

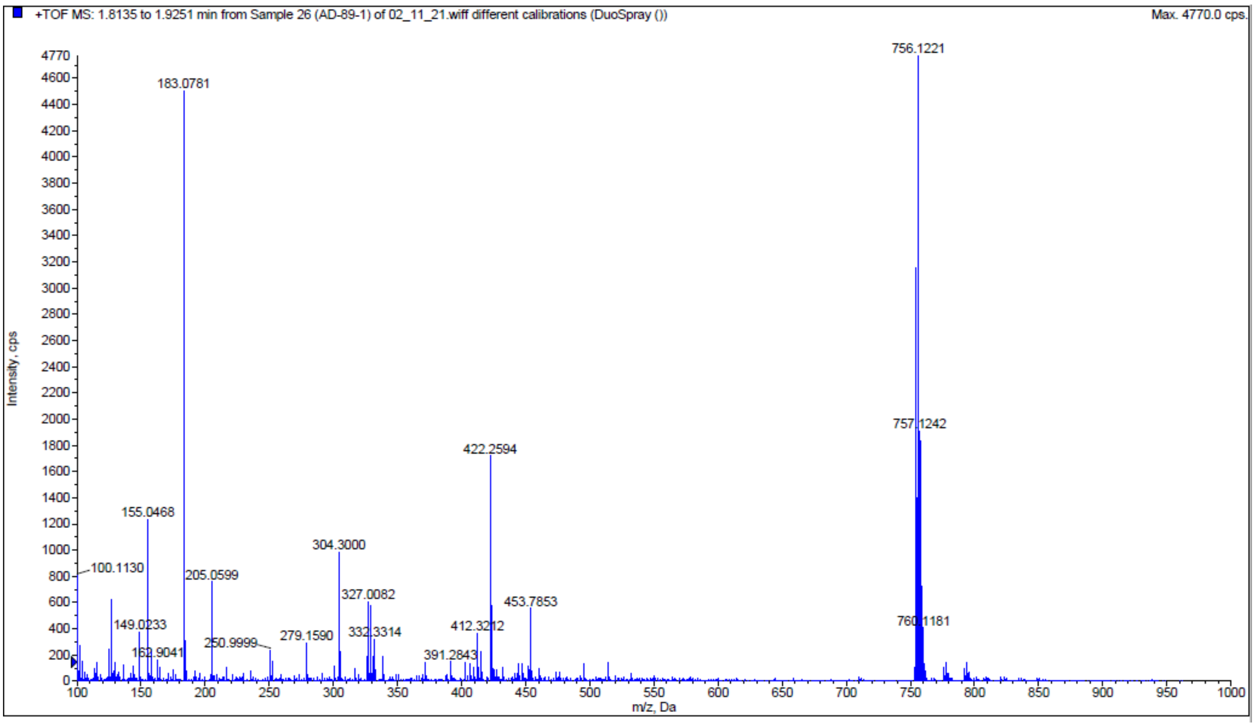

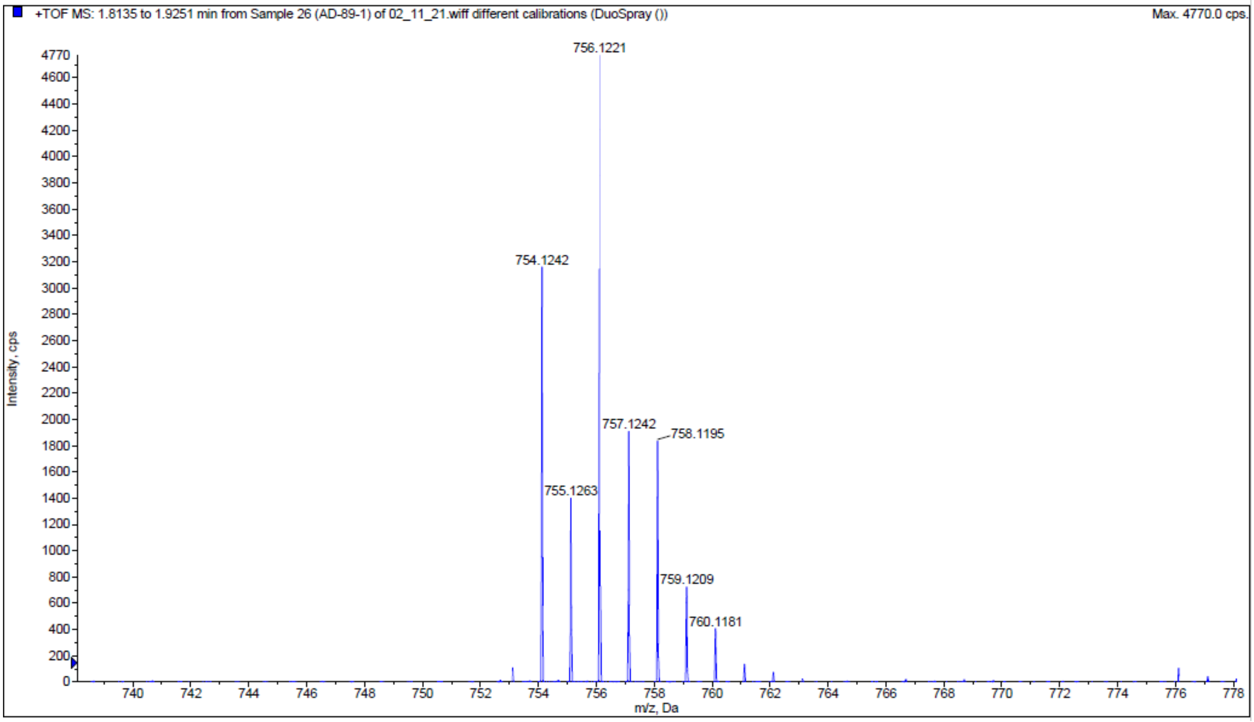

4.11. ESI-HRMS data for complex (^{pBr}**CysNi**)$_{L7}$

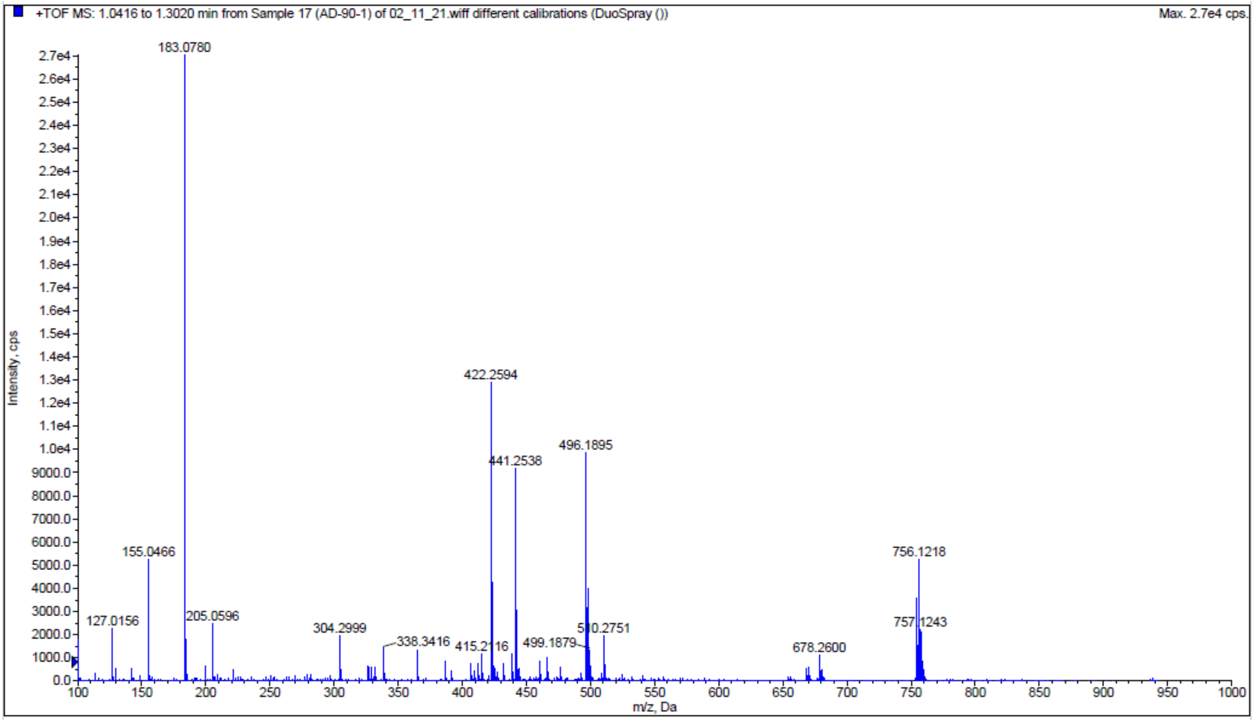

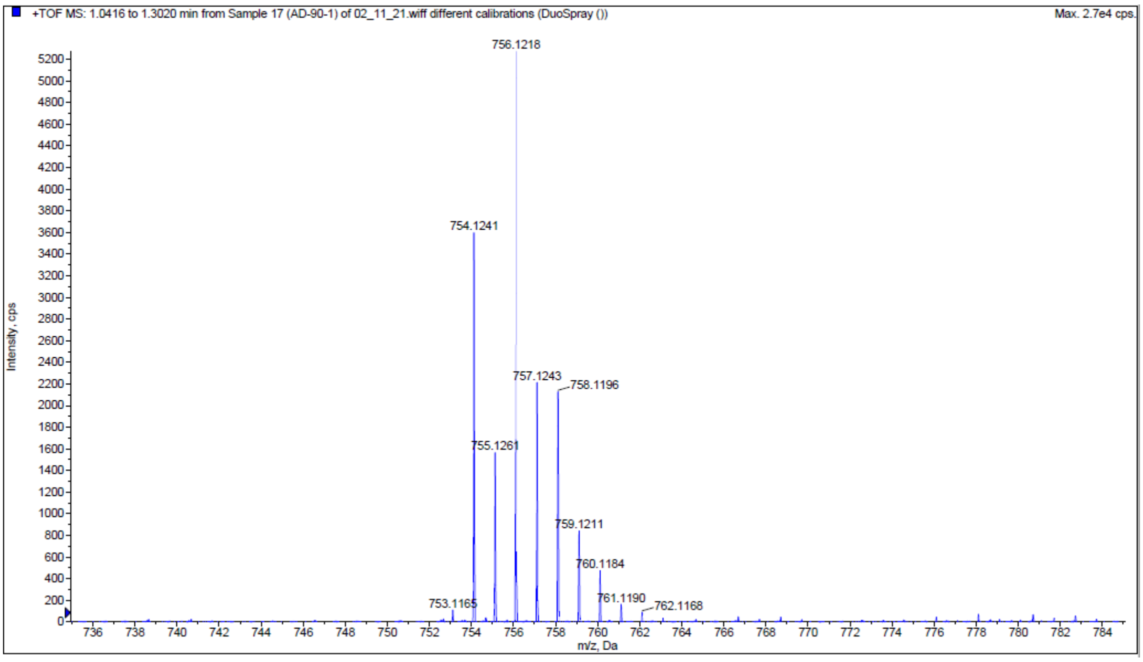

5. IR spectra

5.1. IR spectrum of 2-benzoyl-5-tert-butylaniline

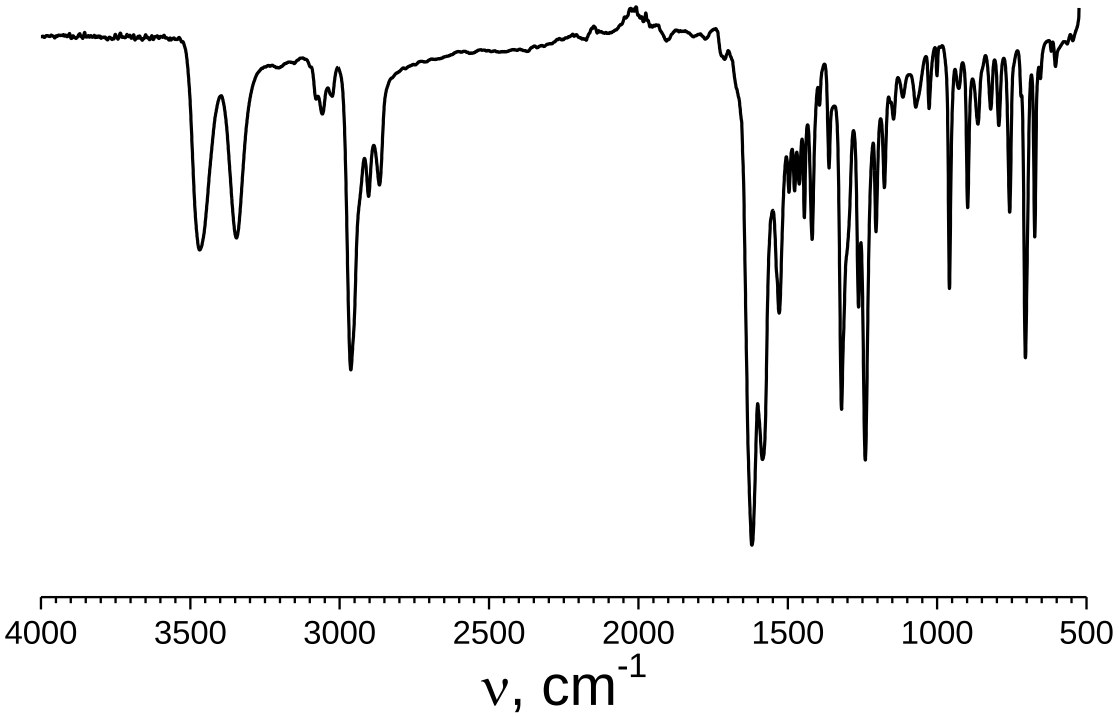

*5.2. IR spectrum of compound **L7***

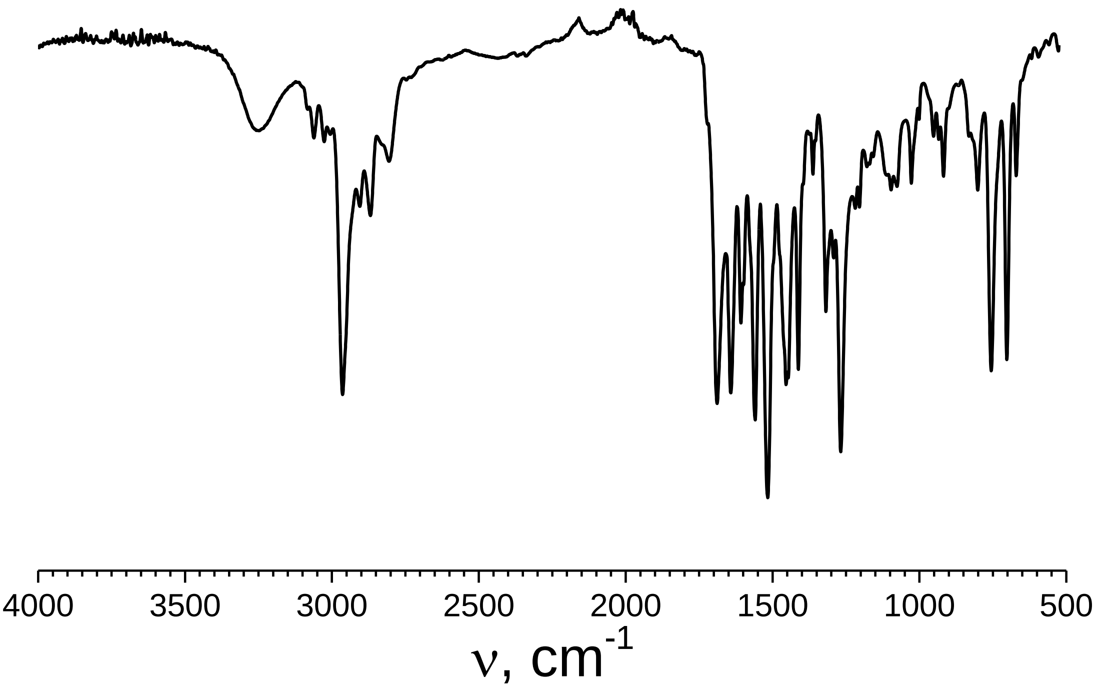

6. HPLC traces

2-[*N*-(*N*'-Benzylprolyl)amino]-5-*tert*-butylbenzophenone (L7) - racemate

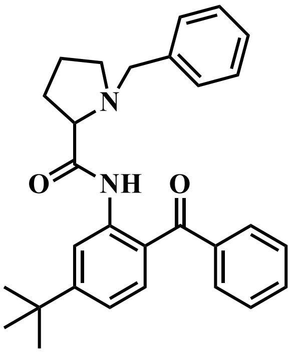

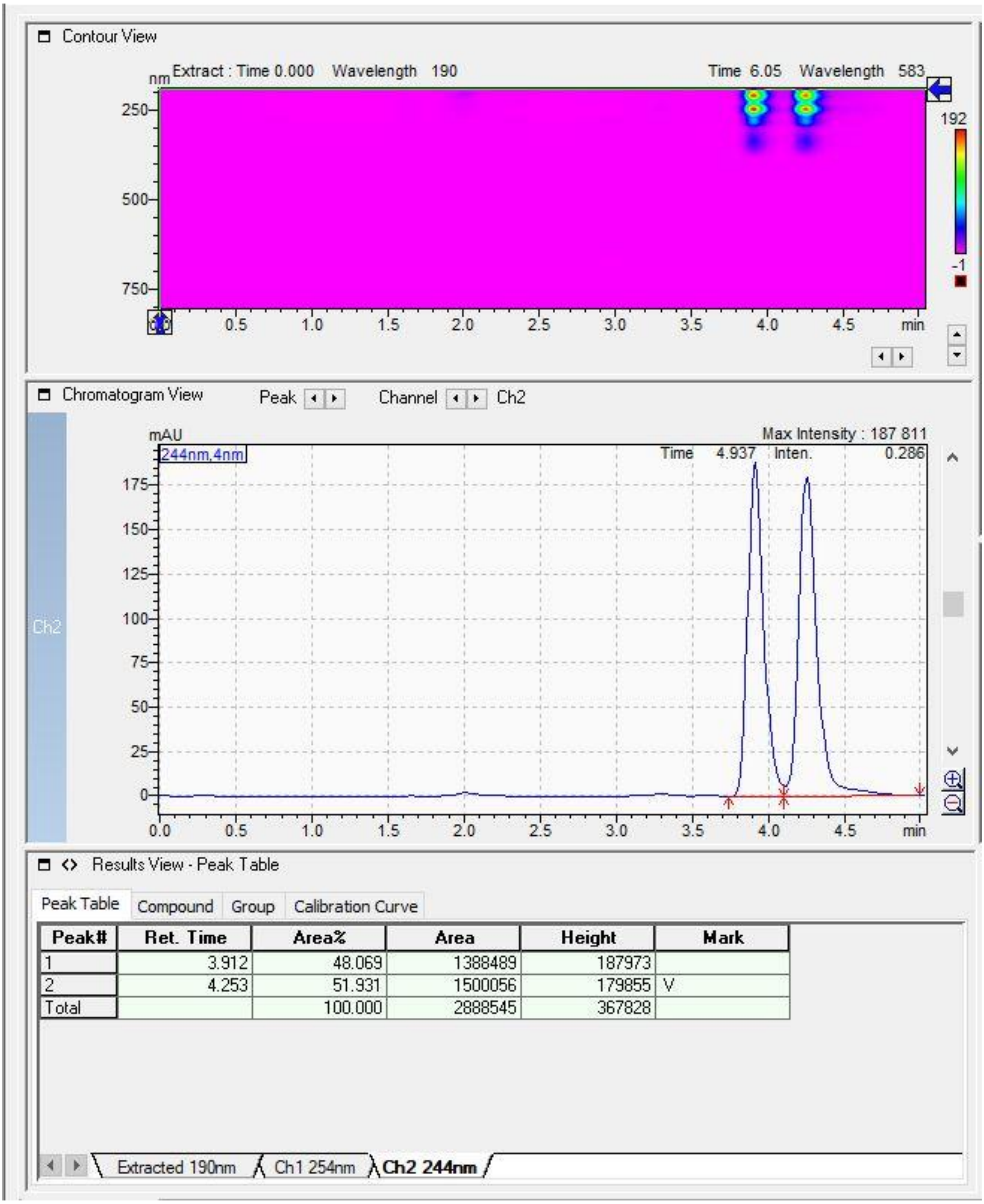

Peak#	Ret. Time	Area%	Area	Height	Mark
1	3.912	48.069	1388489	187973	
2	4.253	51.931	1500056	179855	V
Total		100.000	2888545	367828	

(*S*)-2-[*N*-(*N'*-Benzylprolyl)amino]-5-*tert*-butylbenzophenone (L7) - chiral

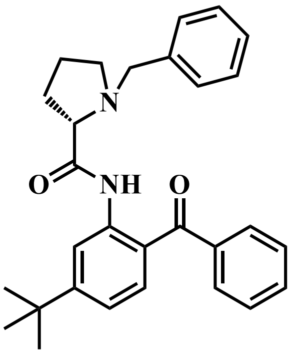

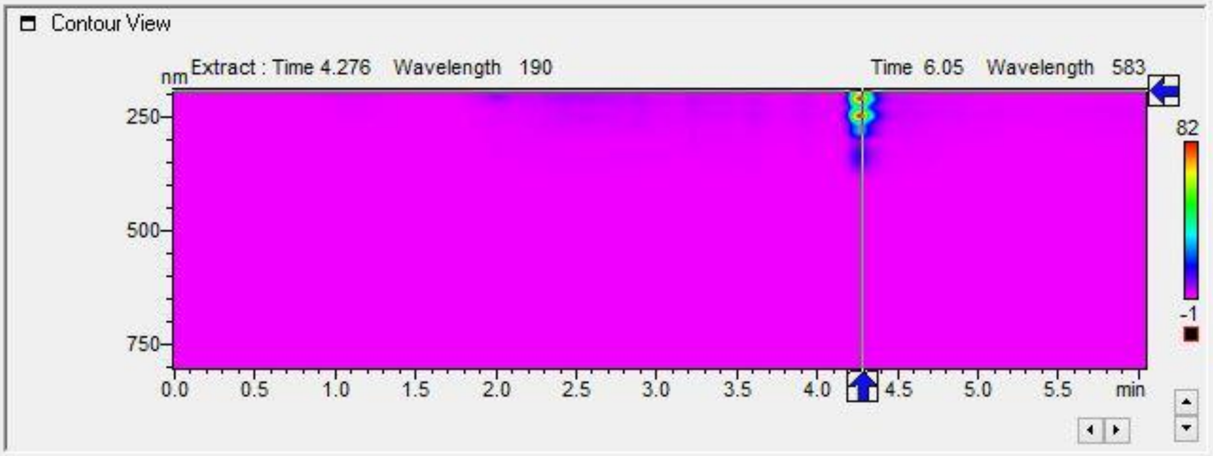

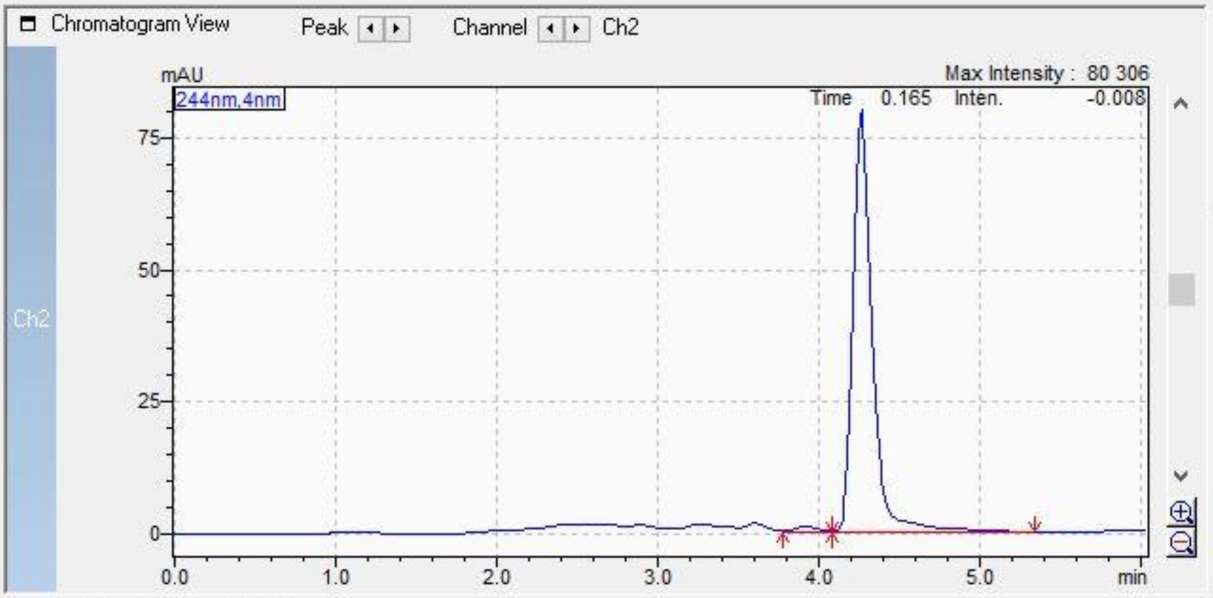

Results View - Peak Table

Peak Table | Compound | Group | Calibration Curve

Peak#	Ret. Time	Area%	Area	Height	Mark
1	3.917	1.859	12648	1252	
2	4.264	98.141	667865	80014	SV
Total		100.000	680513	81266	

Extracted 190nm | Ch1 254nm | **Ch2 244nm** | Ch3 334nm

7. Computational data (energies and Cartesian coordinates of the optimized structures)

(*L*)-(AlaNi)$_{tBu}$	E_e = −3135.407030761794 Hartree

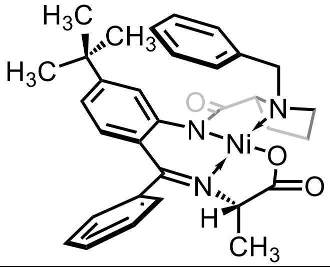

Element	x, Å	y, Å	z, Å
C	0.049346000000	-1.850929000000	0.547212000000
C	0.334662000000	-3.015496000000	1.312603000000
C	-0.636667000000	-3.686261000000	2.039667000000
C	-1.979356000000	-3.236794000000	2.007961000000
C	-2.283090000000	-2.110882000000	1.240249000000
C	-1.304032000000	-1.360751000000	0.528062000000
N	-1.626641000000	-0.172053000000	-0.117515000000
C	1.144695000000	-1.237282000000	-0.185342000000
H	1.369073000000	-3.384122000000	1.337116000000
H	-0.350634000000	-4.569946000000	2.628661000000
C	-3.053874000000	-3.970792000000	2.825212000000
H	-3.311668000000	-1.751868000000	1.173877000000
C	4.732390000000	-2.407747000000	0.367120000000
C	4.827270000000	-3.462099000000	-0.554871000000
C	3.720031000000	-3.795495000000	-1.350561000000
C	2.522133000000	-3.076160000000	-1.228173000000
C	2.424494000000	-2.009466000000	-0.313336000000
C	3.537648000000	-1.683257000000	0.487411000000
H	3.460039000000	-0.859354000000	1.213723000000
H	5.595058000000	-2.146467000000	0.998543000000
H	5.766114000000	-4.028244000000	-0.650530000000
H	3.787337000000	-4.624958000000	-2.070807000000
H	1.646841000000	-3.344176000000	-1.839203000000
N	1.059081000000	-0.044047000000	-0.731661000000
C	2.149047000000	0.536371000000	-1.530815000000
C	2.043386000000	2.070952000000	-1.428264000000
C	2.064221000000	0.112960000000	-3.002513000000
H	3.137214000000	0.234139000000	-1.130747000000
O	2.950510000000	2.807036000000	-1.786331000000
O	0.873365000000	2.474825000000	-0.974315000000
C	-2.932007000000	0.262711000000	-0.268563000000
C	-3.018781000000	1.703955000000	-0.764334000000
O	-3.978192000000	-0.365036000000	-0.090018000000
C	-3.214173000000	1.803880000000	-2.306643000000
N	-1.769401000000	2.465939000000	-0.476007000000
H	-3.878862000000	2.174912000000	-0.239958000000
H	-4.206778000000	2.241679000000	-2.527168000000
H	-3.189940000000	0.803521000000	-2.779615000000
C	-1.692202000000	3.078006000000	0.884068000000
C	-1.677274000000	3.493649000000	-1.550542000000

H	-0.835085000000	3.778112000000	0.850317000000
C	-1.480667000000	2.059631000000	1.978855000000
H	-2.620392000000	3.668766000000	1.059349000000
H	-0.653047000000	3.907584000000	-1.569393000000
H	-2.411384000000	4.303724000000	-1.330264000000
C	-2.072467000000	2.717223000000	-2.798346000000
C	-0.170100000000	1.645486000000	2.298628000000
C	-2.566839000000	1.452834000000	2.633006000000
C	0.042641000000	0.621804000000	3.231786000000
H	0.684583000000	2.135336000000	1.807149000000
C	-2.353140000000	0.427980000000	3.568812000000
H	-3.594894000000	1.766533000000	2.393230000000
H	-3.213401000000	-0.049807000000	4.061641000000
H	1.068313000000	0.299801000000	3.467499000000
C	-1.049368000000	0.005783000000	3.864053000000
H	-0.882795000000	-0.811354000000	4.581871000000
H	-2.377103000000	3.381725000000	-3.629807000000
H	-1.202932000000	2.122112000000	-3.144832000000
Ni	-0.349944000000	1.115731000000	-0.595626000000
H	1.069680000000	0.369977000000	-3.420336000000
H	2.227419000000	-0.976597000000	-3.116020000000
C	-4.448598000000	-3.346606000000	2.648079000000
C	-3.116645000000	-5.448164000000	2.375573000000
C	-2.670545000000	-3.900279000000	4.321722000000
H	-3.422972000000	-4.431207000000	4.941238000000
H	-1.682869000000	-4.362640000000	4.519549000000
H	-2.624479000000	-2.846995000000	4.666306000000
H	-5.191391000000	-3.908564000000	3.249725000000
H	-4.472533000000	-2.290575000000	2.983622000000
H	-4.780425000000	-3.370377000000	1.591171000000
H	-3.878202000000	-5.999684000000	2.965180000000
H	-3.391137000000	-5.523385000000	1.303922000000
H	-2.146381000000	-5.965887000000	2.512199000000
H	2.841767000000	0.656206000000	-3.573654000000

(*D*)-(AlaNi)$_{\mathrm{tBu}}$

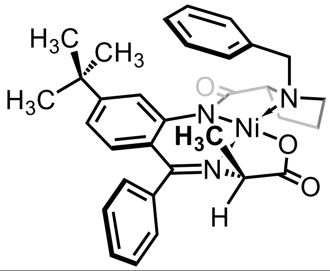

E_e = −3135.401216672444 Hartree

Element	x, Å	y, Å	z, Å
C	0.646555000000	-1.296476000000	-0.125270000000
C	0.826772000000	-2.630007000000	0.344776000000
C	-0.135514000000	-3.297632000000	1.085526000000
C	-1.351356000000	-2.649885000000	1.418103000000
C	-1.574313000000	-1.371379000000	0.905839000000
C	-0.634566000000	-0.677505000000	0.092379000000
N	-0.946832000000	0.544724000000	-0.487176000000
C	1.782355000000	-0.619717000000	-0.727420000000
H	1.774041000000	-3.139526000000	0.127513000000

H	0.067359000000	-4.323554000000	1.426252000000
C	-2.386641000000	-3.358100000000	2.304197000000
H	-2.522708000000	-0.868109000000	1.096406000000
C	5.052960000000	-2.546297000000	-0.228239000000
C	5.338844000000	-2.937146000000	-1.545619000000
C	4.464647000000	-2.580110000000	-2.583993000000
C	3.313083000000	-1.828800000000	-2.307790000000
C	3.023971000000	-1.424509000000	-0.988953000000
C	3.901027000000	-1.796053000000	0.049989000000
H	3.668686000000	-1.501215000000	1.084578000000
H	5.731112000000	-2.828837000000	0.591255000000
H	6.243352000000	-3.525085000000	-1.763020000000
H	4.679022000000	-2.889702000000	-3.618071000000
H	2.622823000000	-1.552175000000	-3.119507000000
N	1.781675000000	0.666194000000	-1.029342000000
C	3.033894000000	1.351326000000	-1.411275000000
C	2.685084000000	2.707306000000	-2.043657000000
C	3.942461000000	1.626544000000	-0.203809000000
O	3.513544000000	3.382364000000	-2.636395000000
O	1.446468000000	3.082088000000	-1.797912000000
C	-2.262779000000	0.955771000000	-0.634571000000
C	-2.384166000000	2.308055000000	-1.325127000000
O	-3.295174000000	0.343818000000	-0.353351000000
C	-2.622593000000	2.163852000000	-2.858394000000
N	-1.140400000000	3.115621000000	-1.193065000000
H	-3.236449000000	2.845988000000	-0.856173000000
H	-3.632464000000	2.539556000000	-3.111947000000
H	-2.583425000000	1.103080000000	-3.171178000000
C	-1.056043000000	3.930527000000	0.056941000000
C	-1.100622000000	3.973404000000	-2.411741000000
H	-0.211722000000	4.631423000000	-0.090897000000
C	-0.818932000000	3.082927000000	1.282504000000
H	-1.992799000000	4.526031000000	0.152264000000
H	-0.086307000000	4.394132000000	-2.527896000000
H	-1.845134000000	4.793560000000	-2.283945000000
C	-1.518155000000	3.015332000000	-3.517205000000
C	0.502457000000	2.760010000000	1.654127000000
C	-1.886833000000	2.509904000000	1.994364000000
C	0.748541000000	1.848378000000	2.688934000000
H	1.339806000000	3.230804000000	1.116890000000
C	-1.642511000000	1.592503000000	3.028713000000
H	-2.924280000000	2.756989000000	1.720699000000
H	-2.487754000000	1.135556000000	3.565258000000
H	1.784599000000	1.596147000000	2.961607000000
C	-0.325645000000	1.251101000000	3.368589000000
H	-0.134802000000	0.519429000000	4.168274000000
H	-1.865672000000	3.546521000000	-4.424270000000
H	-0.646694000000	2.393397000000	-3.806155000000
Ni	0.315475000000	1.780486000000	-1.104755000000
C	-3.622119000000	-2.481035000000	2.570322000000
C	-2.847791000000	-4.654391000000	1.598389000000
C	-1.730914000000	-3.711496000000	3.658942000000
H	-2.457572000000	-4.231701000000	4.317174000000

H	-0.854577000000	-4.377785000000	3.532393000000
H	-1.386211000000	-2.796264000000	4.182321000000
H	-4.341716000000	-3.030274000000	3.210615000000
H	-3.353341000000	-1.542212000000	3.095710000000
H	-4.143925000000	-2.205547000000	1.632411000000
H	-3.588574000000	-5.193305000000	2.225072000000
H	-3.323377000000	-4.424087000000	0.623774000000
H	-2.002944000000	-5.345854000000	1.407210000000
H	3.598945000000	0.753857000000	-2.155210000000
H	4.794182000000	2.244614000000	-0.547694000000
H	3.390156000000	2.188818000000	0.575280000000
H	4.333415000000	0.693343000000	0.241166000000

**(*L*)-(AlaNi)$_H$** 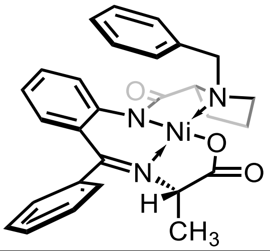		E_e = −2978.473653449693 Hartree	
Element	x, Å	y, Å	z, Å
C	0.656029000000	-1.143152000000	0.013563000000
C	0.949246000000	-2.312686000000	0.769558000000
C	-0.029793000000	-3.017935000000	1.459226000000
C	-1.361707000000	-2.573005000000	1.372224000000
C	-1.691288000000	-1.436015000000	0.639610000000
C	-0.702097000000	-0.655157000000	-0.029019000000
N	-1.012421000000	0.544310000000	-0.658288000000
C	1.757699000000	-0.523956000000	-0.709977000000
H	1.990492000000	-2.659773000000	0.809266000000
H	0.234689000000	-3.911106000000	2.043873000000
H	-2.732307000000	-1.108603000000	0.566560000000
C	5.347849000000	-1.671841000000	-0.129624000000
C	5.455889000000	-2.727585000000	-1.048598000000
C	4.356411000000	-3.069990000000	-1.851186000000
C	3.152925000000	-2.358463000000	-1.738622000000
C	3.042072000000	-1.290652000000	-0.826583000000
C	4.147632000000	-0.954891000000	-0.019195000000
H	4.059810000000	-0.129258000000	0.703981000000
H	6.204497000000	-1.403287000000	0.506893000000
H	6.399052000000	-3.287740000000	-1.136567000000
H	4.434181000000	-3.900538000000	-2.569097000000
H	2.283663000000	-2.633962000000	-2.354895000000
N	1.671835000000	0.664308000000	-1.265282000000
C	2.762445000000	1.236640000000	-2.070228000000
C	2.664509000000	2.771875000000	-1.973859000000
C	2.667502000000	0.807384000000	-3.539575000000
H	3.750376000000	0.930748000000	-1.672802000000
O	3.574693000000	3.502116000000	-2.335742000000
O	1.496785000000	3.182962000000	-1.520448000000
C	-2.316423000000	0.989120000000	-0.806229000000
C	-2.397221000000	2.431860000000	-1.298370000000

O	-3.365543000000	0.367595000000	-0.629632000000
C	-2.603543000000	2.530798000000	-2.839642000000
N	-1.143317000000	3.189462000000	-1.019549000000
H	-3.251986000000	2.905761000000	-0.768179000000
H	-3.592926000000	2.979333000000	-3.053128000000
H	-2.594882000000	1.529480000000	-3.311039000000
C	-1.055916000000	3.807227000000	0.337926000000
C	-1.049907000000	4.210940000000	-2.099983000000
H	-0.207835000000	4.517333000000	0.291366000000
C	-0.815530000000	2.790587000000	1.427846000000
H	-1.989059000000	4.386450000000	0.524403000000
H	-0.023403000000	4.618793000000	-2.125307000000
H	-1.778013000000	5.026599000000	-1.880474000000
C	-1.456316000000	3.430903000000	-3.341719000000
C	0.505757000000	2.397019000000	1.729690000000
C	-1.884018000000	2.157672000000	2.086001000000
C	0.745870000000	1.367369000000	2.649506000000
H	1.346698000000	2.907811000000	1.235815000000
C	-1.643048000000	1.123640000000	3.004820000000
H	-2.919806000000	2.455570000000	1.859720000000
H	-2.489421000000	0.622242000000	3.497303000000
H	1.779669000000	1.063082000000	2.873490000000
C	-0.328832000000	0.721932000000	3.282199000000
H	-0.140642000000	-0.100563000000	3.988266000000
H	-1.758638000000	4.092835000000	-4.176052000000
H	-0.593309000000	2.826271000000	-3.688278000000
Ni	0.268516000000	1.829900000000	-1.134646000000
H	1.673013000000	1.069962000000	-3.953983000000
H	2.821709000000	-0.283860000000	-3.649381000000
H	3.446560000000	1.342286000000	-4.116446000000
H	-2.164445000000	-3.122541000000	1.888650000000

(*D*)-(AlaNi)$_H$

E_e = −2978.468341307136 Hartree

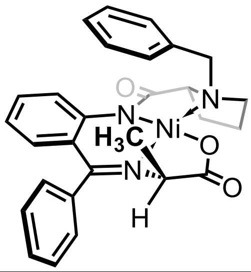

Element	x, Å	y, Å	z, Å
C	0.538818000000	-1.396620000000	-0.370147000000
C	0.712912000000	-2.782816000000	-0.086014000000
C	-0.260224000000	-3.544847000000	0.547250000000
C	-1.455361000000	-2.913824000000	0.936806000000
C	-1.689413000000	-1.576167000000	0.629945000000
C	-0.738549000000	-0.783423000000	-0.081158000000
N	-1.030150000000	0.501230000000	-0.526077000000
C	1.696629000000	-0.669495000000	-0.867978000000
H	1.661879000000	-3.257309000000	-0.365563000000
H	-0.084908000000	-4.610793000000	0.753505000000
H	-2.641482000000	-1.110629000000	0.896646000000
C	4.963736000000	-2.602136000000	-0.380159000000
C	5.307351000000	-2.892879000000	-1.709733000000
C	4.471672000000	-2.468849000000	-2.754548000000
C	3.301116000000	-1.749605000000	-2.472275000000
C	2.954408000000	-1.446024000000	-1.140438000000
C	3.792693000000	-1.884786000000	-0.095773000000
H	3.513523000000	-1.669772000000	0.946852000000
H	5.611264000000	-2.938524000000	0.443738000000
H	6.226727000000	-3.455421000000	-1.931869000000
H	4.731293000000	-2.700063000000	-3.798696000000
H	2.641729000000	-1.420440000000	-3.290080000000
N	1.706099000000	0.635061000000	-1.063671000000
C	2.971598000000	1.346175000000	-1.340983000000
C	2.646417000000	2.727759000000	-1.928289000000
C	3.816281000000	1.552737000000	-0.075755000000
O	3.504831000000	3.443967000000	-2.421759000000
O	1.383530000000	3.066706000000	-1.771145000000
C	-2.332962000000	0.988294000000	-0.528189000000
C	-2.475549000000	2.290337000000	-1.306504000000
O	-3.352808000000	0.465297000000	-0.077019000000
C	-2.821040000000	2.031051000000	-2.804212000000
N	-1.210297000000	3.071696000000	-1.318796000000
H	-3.283489000000	2.879471000000	-0.823947000000
H	-3.800125000000	2.490874000000	-3.041150000000
H	-2.910559000000	0.948887000000	-3.017814000000
C	-1.058181000000	4.018083000000	-0.172845000000
C	-1.180021000000	3.770924000000	-2.635263000000
H	-0.188464000000	4.656171000000	-0.419339000000
C	-0.822498000000	3.292584000000	1.130036000000
H	-1.967842000000	4.659264000000	-0.123014000000
H	-0.154933000000	4.133692000000	-2.829499000000
H	-1.883632000000	4.635102000000	-2.591541000000

C	-1.684341000000	2.704442000000	-3.595931000000
C	0.498347000000	2.999995000000	1.528909000000
C	-1.891398000000	2.809133000000	1.905139000000
C	0.742021000000	2.205342000000	2.656251000000
H	1.334496000000	3.402146000000	0.937808000000
C	-1.649276000000	2.004483000000	3.029512000000
H	-2.928587000000	3.038333000000	1.616434000000
H	-2.496198000000	1.614260000000	3.613440000000
H	1.777324000000	1.979363000000	2.954380000000
C	-0.333458000000	1.692267000000	3.399889000000
H	-0.144167000000	1.054185000000	4.276423000000
H	-2.021590000000	3.129492000000	-4.560973000000
H	-0.866691000000	1.986034000000	-3.810588000000
Ni	0.238306000000	1.738989000000	-1.141153000000
H	3.575834000000	0.789988000000	-2.085973000000
H	4.667402000000	2.212445000000	-0.333233000000
H	3.214869000000	2.040738000000	0.716054000000
H	4.210337000000	0.597637000000	0.317666000000
H	-2.230281000000	-3.478132000000	1.479002000000

**(*L*)-(AlaNi)$_{Cl}$** 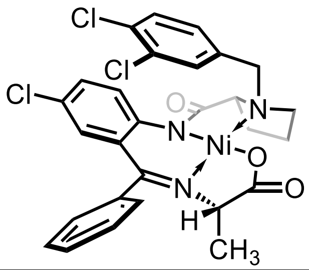	E_e = −4356.300111603811 Hartree

Element	x, Å	y, Å	z, Å
C	0.069273000000	-1.835293000000	0.624953000000
C	0.397688000000	-2.968772000000	1.417846000000
C	-0.571186000000	-3.658691000000	2.134649000000
C	-1.916548000000	-3.257141000000	2.049401000000
C	-2.264656000000	-2.150717000000	1.281287000000
C	-1.295447000000	-1.369566000000	0.582829000000
N	-1.631155000000	-0.185448000000	-0.065497000000
C	1.158520000000	-1.226793000000	-0.131178000000
H	1.442153000000	-3.297701000000	1.478116000000
H	-2.689690000000	-3.812403000000	2.599332000000
H	-3.312579000000	-1.844558000000	1.207577000000
C	4.769958000000	-2.332719000000	0.398303000000
C	4.860533000000	-3.420198000000	-0.485007000000
C	3.743121000000	-3.796878000000	-1.246754000000
C	2.538671000000	-3.087698000000	-1.128257000000
C	2.446198000000	-1.988198000000	-0.251420000000
C	3.569496000000	-1.616953000000	0.515473000000
H	3.487874000000	-0.770328000000	1.213260000000
H	5.640359000000	-2.038802000000	1.003993000000
H	5.803879000000	-3.979370000000	-0.575936000000
H	3.806597000000	-4.652741000000	-1.935505000000
H	1.655409000000	-3.389592000000	-1.711372000000
N	1.047805000000	-0.055986000000	-0.717332000000
C	2.119407000000	0.511073000000	-1.551915000000

C	2.008827000000	2.046920000000	-1.483122000000
C	2.004123000000	0.048749000000	-3.009603000000
H	3.115375000000	0.219616000000	-1.164771000000
O	2.901098000000	2.780043000000	-1.879555000000
O	0.846832000000	2.456481000000	-1.008942000000
C	-2.940357000000	0.252881000000	-0.183055000000
C	-3.039433000000	1.686357000000	-0.700641000000
O	-3.981654000000	-0.366299000000	0.043962000000
C	-3.275731000000	1.762993000000	-2.238722000000
N	-1.783772000000	2.455106000000	-0.457557000000
H	-3.885732000000	2.164151000000	-0.160763000000
H	-4.270991000000	2.204203000000	-2.438430000000
H	-3.270163000000	0.755448000000	-2.696231000000
C	-1.673839000000	3.090040000000	0.889969000000
C	-1.718222000000	3.463292000000	-1.553018000000
H	-0.825083000000	3.797550000000	0.822825000000
C	-1.421365000000	2.082418000000	1.985445000000
H	-2.602488000000	3.671875000000	1.087459000000
H	-0.694512000000	3.875679000000	-1.603946000000
H	-2.446954000000	4.276694000000	-1.328802000000
C	-2.142980000000	2.661948000000	-2.774752000000
C	-0.100417000000	1.653835000000	2.227379000000
C	-2.475246000000	1.473296000000	2.685891000000
C	0.155818000000	0.612074000000	3.129156000000
H	0.743385000000	2.132357000000	1.710121000000
C	-2.224610000000	0.431885000000	3.590233000000
H	-3.513893000000	1.788445000000	2.505748000000
C	-0.912683000000	-0.013889000000	3.810890000000
H	-2.463185000000	3.308397000000	-3.614153000000
H	-1.282261000000	2.056335000000	-3.125183000000
Ni	-0.365317000000	1.099287000000	-0.586991000000
H	1.002377000000	0.295847000000	-3.416141000000
H	2.163115000000	-1.044005000000	-3.093958000000
H	2.771829000000	0.574602000000	-3.609294000000
Cl	-0.121796000000	-5.012764000000	3.139745000000
Cl	-0.623014000000	-1.331119000000	4.898040000000
H	-3.047557000000	-0.063128000000	4.123342000000
Cl	1.792724000000	0.084561000000	3.384574000000

(*D*)-(AlaNi)$_{Cl}$

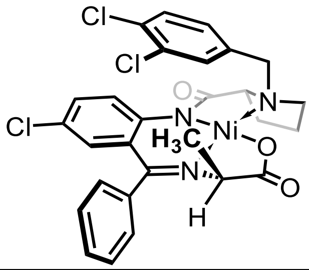

E_e = −4356.294154683548 Hartree

Element	x, Å	y, Å	z, Å
C	0.025143000000	-1.946741000000	0.415901000000
C	0.337951000000	-3.185206000000	1.047545000000
C	-0.579235000000	-3.853440000000	1.846167000000
C	-1.866525000000	-3.316559000000	2.029058000000
C	-2.217202000000	-2.137634000000	1.382946000000

C	-1.307803000000	-1.413255000000	0.554257000000
N	-1.681411000000	-0.237273000000	-0.083114000000
C	1.105078000000	-1.285327000000	-0.310934000000
H	1.335628000000	-3.619744000000	0.922364000000
H	-2.592152000000	-3.833049000000	2.673193000000
H	-3.233148000000	-1.747592000000	1.488818000000
C	4.484347000000	-2.989108000000	0.257933000000
C	4.710425000000	-3.567646000000	-1.001245000000
C	3.763999000000	-3.403665000000	-2.024449000000
C	2.597494000000	-2.659338000000	-1.792533000000
C	2.370025000000	-2.066640000000	-0.534661000000
C	3.320500000000	-2.242686000000	0.492472000000
H	3.132501000000	-1.794773000000	1.479819000000
H	5.218651000000	-3.120945000000	1.066788000000
H	5.624254000000	-4.152833000000	-1.183575000000
H	3.930708000000	-3.862172000000	-3.010747000000
H	1.848037000000	-2.540321000000	-2.590077000000
N	1.021779000000	-0.054524000000	-0.777596000000
C	2.199571000000	0.604749000000	-1.390488000000
C	1.734324000000	1.894499000000	-2.084666000000
C	3.277437000000	1.012675000000	-0.374764000000
O	2.448571000000	2.518145000000	-2.854246000000
O	0.541747000000	2.288833000000	-1.683653000000
C	-3.011000000000	0.149951000000	-0.175581000000
C	-3.191893000000	1.517431000000	-0.821760000000
O	-4.018412000000	-0.486541000000	0.139119000000
C	-3.531346000000	1.419739000000	-2.339185000000
N	-1.953116000000	2.341309000000	-0.746939000000
H	-4.018728000000	2.027717000000	-0.281058000000
H	-4.565000000000	1.776360000000	-2.509911000000
H	-3.488087000000	0.371593000000	-2.691157000000
C	-1.787918000000	3.113279000000	0.524241000000
C	-2.018780000000	3.246886000000	-1.930530000000
H	-0.963473000000	3.829540000000	0.342379000000
C	-1.460338000000	2.220428000000	1.695316000000
H	-2.719963000000	3.693650000000	0.709968000000
H	-1.023983000000	3.691560000000	-2.106675000000
H	-2.768627000000	4.043582000000	-1.716693000000
C	-2.493998000000	2.320814000000	-3.039624000000
C	-0.119521000000	1.844966000000	1.910324000000
C	-2.467205000000	1.627252000000	2.474198000000
C	0.201520000000	0.850989000000	2.844514000000
H	0.686987000000	2.322402000000	1.336780000000
C	-2.153730000000	0.631791000000	3.410052000000
H	-3.520268000000	1.907295000000	2.322538000000
H	-2.941644000000	0.139562000000	3.995988000000
C	-0.824150000000	0.218965000000	3.584092000000
H	-2.914341000000	2.876396000000	-3.899610000000
H	-1.630046000000	1.732136000000	-3.407963000000
Ni	-0.482804000000	1.015512000000	-0.807823000000
H	2.655647000000	-0.061374000000	-2.150657000000
H	3.776624000000	0.139134000000	0.079921000000
H	2.841111000000	1.627160000000	0.436333000000

H	4.031951000000	1.617740000000	-0.913251000000
Cl	-0.133154000000	-5.349623000000	2.628590000000
Cl	-0.460214000000	-1.077078000000	4.673124000000
Cl	1.861596000000	0.380045000000	3.059972000000

8. Frontier orbitals of the $(GlyNi)_{L7}$ and its radical anion

DFT calculated frontier orbitals for $(GlyNi)_{L7}$

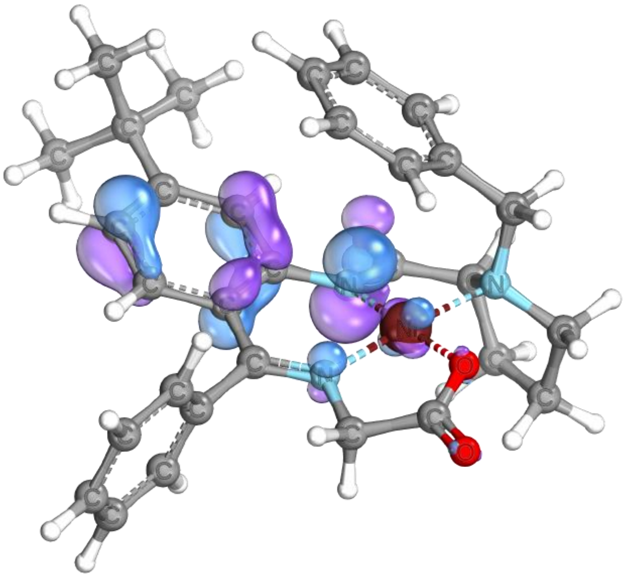

HOMO

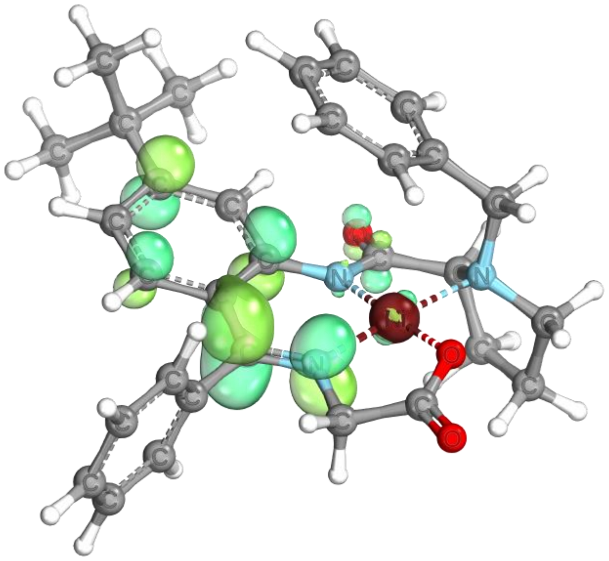

LUMO

(note that SOMO and spin density in the radical anion has the other localization)

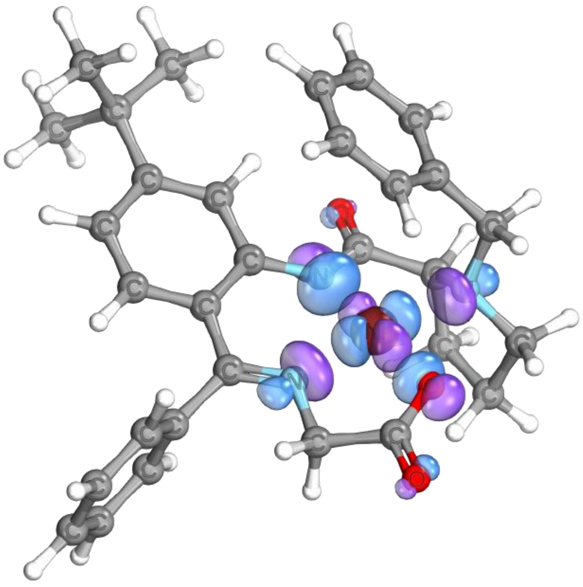

SOMO of the radical anion of (GlyNi)$_{L7}$

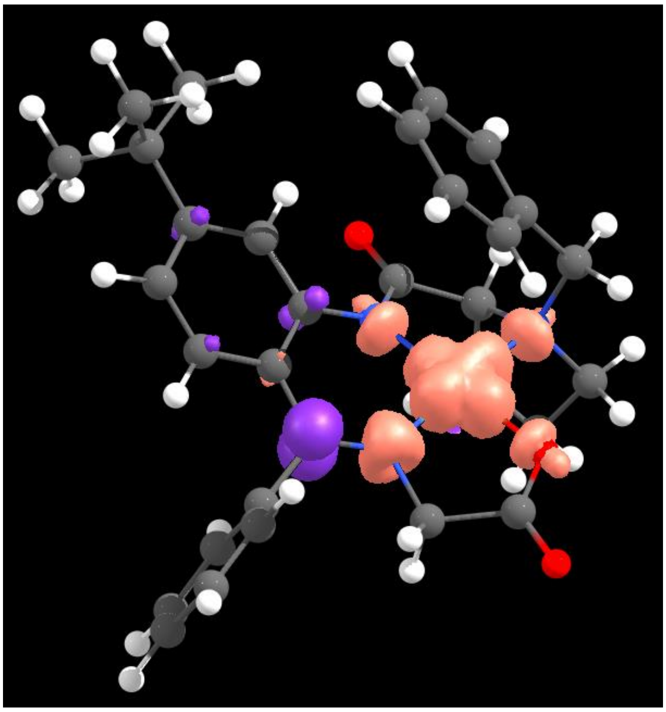

Spin density localization in the radical anion of (GlyNi)$_{L7}$

9. References

1. Neese, F. *Wiley Interdiscip. Rev.: Comput. Mol. Sci.* **2018,** *8,* e1327.

2. Perdew, J. P.; Burke, K.; Ernzerhof, M. *Phys. Rev. Lett.* **1997,** *78,* 1396–1396. doi:10.1103/PhysRevLett.78.1396

3. Caldeweyher, E.; Ehlert, S.; Hansen, A.; Neugebauer, H.; Spicher, S.; Bannwarth, C.; Grimme, S. *J. Chem. Phys.* **2019,** *150,* 154122. doi:10.1063/1.5090222

4. Weigend, F.; Ahlrichs, R. *Phys. Chem. Chem. Phys.* **2005,** *7,* 3297. doi:10.1039/b508541a

5. Weigend, F. *Phys. Chem. Chem. Phys.* **2006,** *8,* 1057. doi:10.1039/b515623h

6. Lu, T.; Chen, F. *J. Comput. Chem.* **2012,** *33,* 580–592. doi:10.1002/jcc.2288

7. Ayyangar, N. R.; Lahoti, R. J.; Srinivasan, K. V.; Daniel, T. *Synthesis (Stuttg).* **1991**, *1991*, 322–324. doi:10.1055/s-1991-26457

8. Belokon, Y. N.; Tararov, V. I.; Maleev, V. I.; Savel'eva, T. F.; Ryzhov, M. G. *Tetrahedron: Asymmetry* **1998**, *9*, 4249–4252

9. Levitskiy, O. A.; Grishin, Y. K.; Magdesieva, T. V. *European J. Org. Chem.* **2019**, *2019*, 3174–3182. doi:10.1002/ejoc.201900466

10. Belokon, Y. N.; Sagyan, A. S.; Djamgaryan, S. M.; Bakhmutov, V. I.; Belikov, V. M. *Tetrahedron* **1988**, *44*, 5507–5514. doi:https://doi.org/10.1016/S0040-4020(01)86056-7

11. Yamamoto, J.; Kawashima, A.; Kawamura, A.; Abe, H.; Moriwaki, H.; Shibata, N.; Soloshonok, V. A. *European J. Org. Chem.* **2017**, *2017*, 1931–1939. doi:10.1002/ejoc.201700018
